# Cardiomyocytes disrupt pyrimidine biosynthesis in nonmyocytes to regulate heart repair

**DOI:** 10.1172/JCI149711

**Published:** 2022-01-18

**Authors:** Shen Li, Tomohiro Yokota, Ping Wang, Johanna ten Hoeve, Feiyang Ma, Thuc M. Le, Evan R. Abt, Yonggang Zhou, Rimao Wu, Maxine Nanthavongdouangsy, Abraham Rodriguez, Yijie Wang, Yen-Ju Lin, Hayato Muranaka, Mark Sharpley, Demetrios T. Braddock, Vicky E. MacRae, Utpal Banerjee, Pei-Yu Chiou, Marcus Seldin, Dian Huang, Michael Teitell, Ilya Gertsman, Michael Jung, Steven J. Bensinger, Robert Damoiseaux, Kym Faull, Matteo Pellegrini, Aldons J. Lusis, Thomas G. Graeber, Caius G. Radu, Arjun Deb

**Affiliations:** 1 Division of Cardiology, Department of Medicine and; 2 UCLA Cardiovascular Theme, David Geffen School of Medicine, UCLA, Los Angeles, California, USA.; 3 Department of Molecular, Cell and Developmental Biology, College of Life Sciences,; 4 Eli and Edythe Broad Center of Regenerative Medicine and Stem Cell Research,; 5 Molecular Biology Institute,; 6 California Nanosystems Institute, and; 7 UCLA Metabolomics Center, Crump Institute of Molecular Imaging, California Nanosystems Institute, UCLA, Los Angeles, California, USA.; 8 Jonsson Comprehensive Cancer Center and; 9 Department of Molecular and Medical Pharmacology, David Geffen School of Medicine, UCLA, Los Angeles, California, USA.; 10 Department of Bioengineering, Samueli School of Engineering at UCLA, Los Angeles, California, USA.; 11 Department of Mechanical and Aerospace Engineering and; 12 Department of Microbiology, Immunology and Molecular Genetics, UCLA, Los Angeles, California, USA.; 13 Department of Pathology, Yale University, New Haven, Connecticut, USA.; 14 Division of Functional Genetics and Development, The Roslin Institute and R(D)VS, University of Edinburgh, Edinburgh, United Kingdom.; 15 Department of Biological Chemistry, David Geffen School of Medicine, UCLA, Los Angeles, California, USA.; 16 Department of Biological Chemistry and Center for Epigenetics and Metabolism, University of California, Irvine, Irvine, California, USA.; 17 Department of Pathology, David Geffen School of Medicine, UCLA, Los Angeles, California, USA.; 18 Clarus Analytical, San Diego, California, USA.; 19 Department of Chemistry, College of Physical Sciences, UCLA, Los Angeles, California, USA.; 20 Pasarow Mass Spectrometry Laboratory, Jane and Terry Semel Institute for Neuroscience and Human Behavior and Department of Psychiatry and Biobehavioral Sciences, David Geffen School of Medicine at UCLA, Los Angeles, California, USA.

**Keywords:** Cardiology, Cardiovascular disease

## Abstract

Various populations of cells are recruited to the heart after cardiac injury, but little is known about whether cardiomyocytes directly regulate heart repair. Using a murine model of ischemic cardiac injury, we demonstrate that cardiomyocytes play a pivotal role in heart repair by regulating nucleotide metabolism and fates of nonmyocytes. Cardiac injury induced the expression of the ectonucleotidase ectonucleotide pyrophosphatase/phosphodiesterase 1 (ENPP1), which hydrolyzes extracellular ATP to form AMP. In response to AMP, cardiomyocytes released adenine and specific ribonucleosides that disrupted pyrimidine biosynthesis at the orotidine monophosphate (OMP) synthesis step and induced genotoxic stress and p53-mediated cell death of cycling nonmyocytes. As nonmyocytes are critical for heart repair, we showed that rescue of pyrimidine biosynthesis by administration of uridine or by genetic targeting of the ENPP1/AMP pathway enhanced repair after cardiac injury. We identified ENPP1 inhibitors using small molecule screening and showed that systemic administration of an ENPP1 inhibitor after heart injury rescued pyrimidine biosynthesis in nonmyocyte cells and augmented cardiac repair and postinfarct heart function. These observations demonstrate that the cardiac muscle cell regulates pyrimidine metabolism in nonmuscle cells by releasing adenine and specific nucleosides after heart injury and provide insight into how intercellular regulation of pyrimidine biosynthesis can be targeted and monitored for augmenting tissue repair.

## Graphical abstract

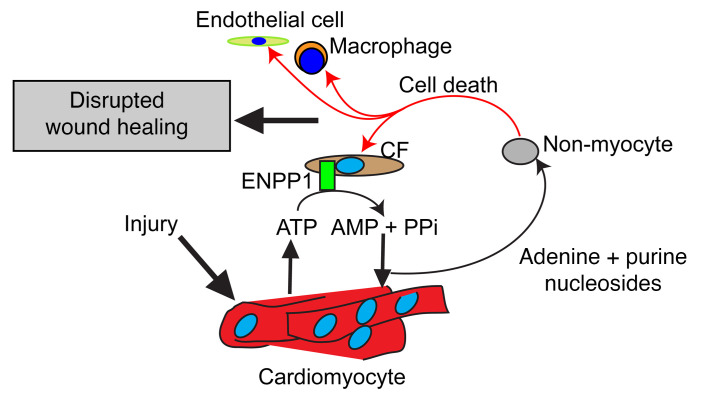

## Introduction

Myocardial infarction (MI) is a leading cause of systolic heart failure (1). After MI, different cell types are recruited to the heart in a spatiotemporally regulated manner to contribute to wound healing. An initial polymorphonuclear infiltrate is replaced by macrophages, and vital cues provided by inflammatory cells initiate fibroblast and endothelial cell proliferation to form granulation tissue (2, 3) that subsequently matures to form a scar (4). The presence of different types of cells within the injured heart enables cell-cell crosstalk and regulation of different phases of wound healing (3, 5). Although the cellular events in cardiac repair have been well studied, the role of the cardiac muscle cell itself in regulating cardiac wound healing is unclear. The cardiac muscle cell does not possess any ontogenetic memory of acute injury, and whether it can directly regulate organ repair remains an unanswered question.

In this report, we demonstrate that the cardiac muscle cell plays a pivotal role in regulating the cardiac repair response by releasing metabolites that disrupt cellular function of nonmyocyte cells, such as macrophages, fibroblasts, and endothelial and smooth muscle cells, and worsen wound healing. Following ischemic cardiac injury, extracellular ATP levels increase in the injured region from extravasation of intracellular ATP from necrotic myocytes, increased membrane permeability, and upregulated activity of nucleotide transporters (6–8). ATP is a damage-associated molecular pattern (DAMP) signal associated with acute injury (9). Increased accumulation of extracellular ATP is limited by tissue ectonucleotidases, which are membrane-bound enzymes with an extracellular catalytic domain that hydrolyzes extracellular ATP. We show that the ectonucleotidase ectonucleotide pyrophosphatase/phosphodiesterase 1 (ENPP1) (10) is induced after cardiac injury and is the principal nucleotidase that hydrolyzes extracellular ATP in the injured heart. AMP generated from ENPP1 activity induces the cardiomyocyte to release adenine base and specific purine nucleoside that in combination disrupt pyrimidine biosynthesis in nonmyocytes. Pyrimidine biosynthetic defects adversely alter purine/pyrimidine ratios in nonmyocytes during cell proliferation and induce a DNA damage response that leads to cell death of nonmyocytes. Macrophages, endothelial cells, and fibroblasts are critical components of cardiac repair, and depletion or functional impairment of nonmyocyte cells is known to worsen cardiac wound healing (11). We demonstrate that genetic or pharmacologic targeting of the ENPP1/AMP cascade in the injured heart attenuates cardiomyocyte-induced defects of pyrimidine biosynthesis in nonmyocytes and leads to superior postinfarct cardiac function. Taken together, these observations shed insight into the role of the cardiac muscle cell in regulating nucleotide metabolism and cellular function of nonmyocytes and into how such myocyte-induced defects of pyrimidine biosynthesis in cycling nonmyocytes can be specifically targeted to enhance repair and postinjury heart function.

## Results

### Cardiac injury induces expression of ENPP1, which is the principal ectonucleotidase that hydrolyzes extracellular ATP.

**Figure 1:**
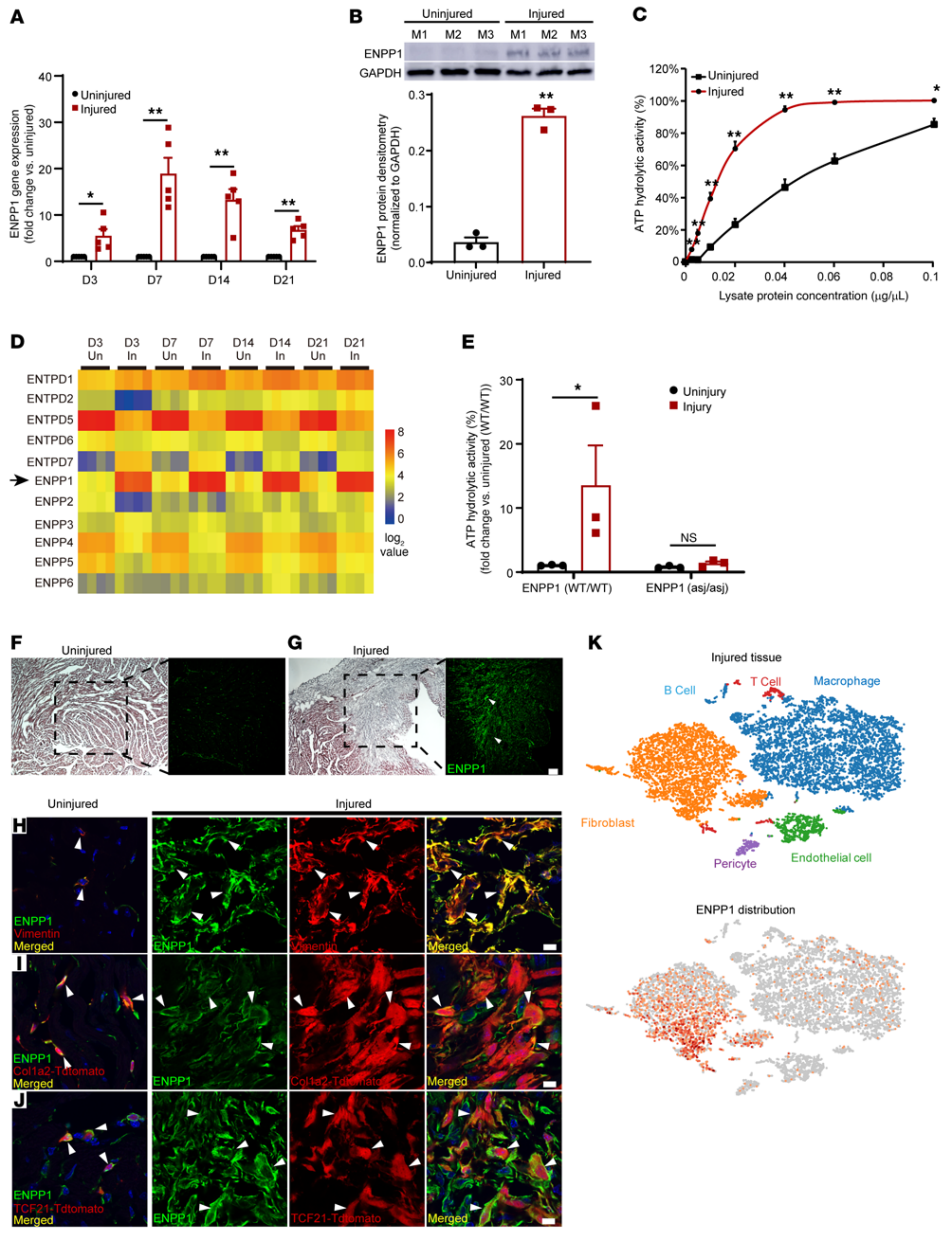
ENPP1 is expressed in the infarcted heart by nonmyocytes and is the principal ectonucleotidase that hydrolyzes extracellular ATP. (**A**) qPCR demonstrating ENPP1 gene expression in the injured region of the heart compared with uninjured regions at 3, 7, 14, and 21 days after MI (*n =* 5). (**B**) Western blotting and quantitative densitometry demonstrating ENPP1 protein levels in injured and uninjured regions of the heart at 7 days following MI (*n =* 3). (**C**) ATP hydrolytic activity at various concentrations of the injured heart tissue homogenate compared with that in uninjured tissue homogenate 7 days after MI (*n =* 3). (**D**) Heatmap with gene expression patterns of ENPP1 (arrow) and other members of the ENPP and ectonucleotidase family in the injured versus uninjured regions of the heart (*n =* 4/time point). (**E**) ATP hydrolytic activity at 7 days after MI in WT mice and ENPP1^asj/asj^ mutant mice (*n =* 3). (**F** and **G**) H&E staining and immunostaining for ENPP1 (green, arrowheads) in the uninjured (**F**) and injured (**G**) regions at day 7 after MI. Scale bar: 100 μm (high magnification). Low magnification: ×4. (**H**) Immunostaining for ENPP1 and vimentin in the uninjured and injured regions at 7 days after MI (arrowheads indicate ENPP1 and vimentin colocalization in merged images). (**I** and **J**) Immunostaining of ENNP1 expression in genetically labeled CFs in (**I**) Col1a2CreERT:R26R^tdtomato^ or (**J**) TCF21MCM:R26R^tdtomato^ mice at 7 days after MI (arrowheads indicate where ENPP1-expressing cells coexpress the fibroblast tdTomato label, representative images; *n =* 3). Scale bars: 10 μm. (**K**) Single-cell RNA-Seq of nonmyocytes at 7 days after MI demonstrating cell phenotypes in clusters and ENPP1 distribution (*n =* 3). Data are represented as mean ± SEM. **P < 0.01; *P < 0.05, 2-tailed Student’s *t* test (**A**–**C** and **E**).

We subjected adult mice to ischemic cardiac injury by permanent ligation of the left anterior descending coronary artery and performed quantitative PCR (qPCR) on injured and uninjured regions of hearts at 3, 7, 14, and 21 days after injury. ENPP1 expression increased by 5-fold on day 3 and was 15- to 20-fold higher at day 7 compared with that of uninjured regions (Figure 1A). Western blotting confirmed increased ENPP1 protein expression (Figure 1B). We next measured ENPP1 enzymatic activity and observed that ATP hydrolytic activity was significantly increased in the injured tissue homogenates, suggesting that increased ENPP1 protein was associated with increased ectonucleotidase activity (Figure 1C). As there are several members of the ectonucleotidase family (12, 13) that hydrolyze extracellular ATP, we analyzed RNA-Seq data sets of acute cardiac injury (14) and observed that, of the known mammalian ectonucleotidases, ENPP1 was the only one that demonstrated the most early, robust, and consistent increase in expression (Figure 1D). To confirm that increased ectonucleotidase activity in injured cardiac tissue is predominantly due to increased ENPP1, we subjected ENPP1 mutant mice (ENPP1^asj/asj^ mice) to ischemic cardiac injury. The ENPP1^asj/asj^ mice have an amino acid substitution in the catalytic domain that renders the catalytic domain devoid of ATP hydrolytic activity (15). In contrast with that of WT mice, injured heart tissue from ENPP1^asj/asj^ animals did not show increased ATP hydrolytic activity (Figure 1E). Taken together, these observations demonstrate that following cardiac injury, ENPP1 expression and activity increase significantly and that ENPP1 is the principal nucleotidase that hydrolyzes extracellular ATP.

Immunostaining demonstrated a robust increase in ENPP1 expression that was predominantly restricted to the injury region (Figure 1, F and G). To identify the phenotype of the cell expressing ENPP1, we isolated myocytes and nonmyocytes from the injured heart and observed that ENPP1 expression was restricted to the nonmyocyte fraction of cells (Supplemental Figure 1A; supplemental material available online with this article; https://doi.org/10.1172/JCI149711DS1). Expression of ENPP1 was almost 100-fold higher in nonmyocytes (Supplemental Figure 1B), and immunostaining demonstrated that ENPP1-expressing cells resided in the myocardial interstitium (Supplemental Figure 1C) and costained for the cardiac fibroblast (CF) marker vimentin (Figure 1H). To confirm this observation, we performed immunostaining in mice harboring genetically labeled CFs. We crossed Col1a2CreERT2 (16) mice or TCF21MerCreMer (MCM; ref. 17) (TCF21 and Col1a2 being fibroblast Cre drivers) mice with the lineage reporter Rosa26tdTomato mice. Mice were administered tamoxifen to label fibroblasts and immunostaining of injured heart sections demonstrated ENPP1 to be expressed by genetically labeled CFs (Figure 1, I and J). Flow cytometry on the injured heart demonstrated a 2-fold increase in the number of ENPP1-expressing cells (Supplemental Figure 1D), and 68%, 72%, and 86% of ENPP1-expressing cells coexpressed the fibroblast markers CD90.2, TCF21MCM-induced tdTomato label, and MEFSK4, respectively (Supplemental Figure 1D). Macrophages and endothelial cells made up the remaining fraction of nonmyocyte cells expressing ENPP1 (Supplemental Figure 1, E and F). We also analyzed single-cell RNA-Seq data sets of the nonmyocyte fraction (14) isolated at 7 days after ischemic cardiac injury and, consistent with flow cytometry and immunostaining, observed that ENPP1 was primarily expressed by CFs and, to a lesser degree, by macrophages and endothelial cells (Figure 1K).

### Increased ENPP1 expression in nonmyocytes induces cardiomyocytes to secrete proapoptotic molecules that cause cell death of nonmyocytes.

**Figure 2:**
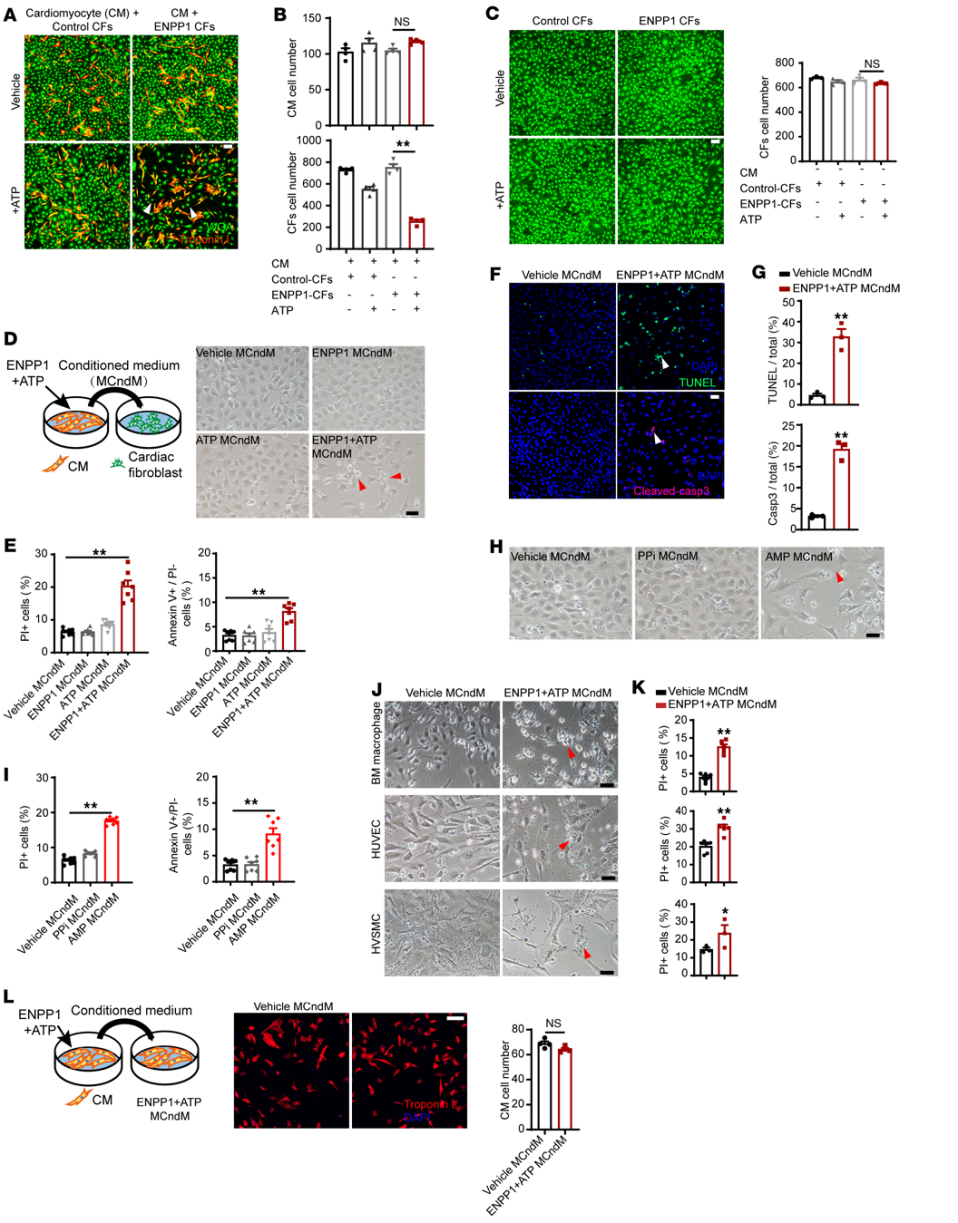
ENPP1 in the presence of ATP induces cardiomyocytes to release proapoptotic molecules. (**A**) Coculture of rat ventricular cardiomyocytes (CMs, red) with control CF or ENPP1-CF (green) with or without ATP (arrowheads show decrease in ENPP1 CF). Scale bar: 100 μm. (**B**) Number of CMs and CFs following 48 hours of coculture (*n =* 4). (**C**) Control CFs or ENPP1 CFs (green) in the presence or absence of added ATP but without any cardiomyocytes, and quantitation of cell numbers after 48 hours of ATP/vehicle addition (*n =* 3). Scale bar: 100 μm. (**D**) Transfer of control or ENPP1+ATP MCndM to CFs and images 48 hours later demonstrating decreased numbers of CFs treated with ENPP1+ATP MCndM (arrowheads). Scale bar: 50 μm. (**E**) Flow cytometry to demonstrate the fraction of dead (PI^+^) or apoptotic cells (annexin V^+^, PI^–^) following treatment with ENPP1+ATP MCndM or control MCndM (*n =* 7). (**F**) TUNEL and caspase staining (arrowheads) of CFs treated with vehicle MCndM or ENPP1+ATP MCndM and (**G**) quantitation (*n =* 3). Scale bar: 50 μm. (**H**) CFs treated with vehicle MCndM, PPi MCndM, or AMP MCndM for 48 hours showing loss of cells with treatment with AMP MCndM (arrowheads) and (**I**) quantitation of dead cells (*n =* 7). Scale bar: 50 μm. (**J**) Treatment of macrophages, HUVECs, and human vascular smooth muscle cells (hVSMCs) with vehicle MCndM or ENPP1+ATP MCndM and (**K**) corresponding flow cytometry to determine cell death (arrowheads). (*n =* 6, BM macrophages; *n =* 6, HUVECs, *n =* 3 hVSMCs). Scale bars: 50 μm. (**L**) ENPP1+ATP MCndM does not cause cell death when added to myocytes (*n =* 4). Scale bar: 100 μm. Data are represented as mean ± SEM. **P < 0.01; *P < 0.05, ordinary 1-way ANOVA with Tukey’s multiple comparison test (**B**, **C**, **E**, and **I**) or 2-tailed Student’s *t* test (**G**, **K**, and **L**).

Extracellular ATP is a DAMP signal and is known to increase by several orders of magnitude after tissue injury (18). We hypothesized that ENPP1 via its ATP hydrolytic activity regulates intercellular communication between myocytes and nonmyocytes in the injured region. We first overexpressed the mouse ENPP1 gene in CFs using a lentivirus and immortalized ENPP1-overexpressing CFs with the SV40 antigen (ENPP1-CFs) to avoid culture-induced senescence and variation associated with primary cell isolation (Supplemental Figure 2, A and B). Control CFs infected with an empty lentivirus were also immortalized in a similar manner. To determine the role of ENPP1 in mediating myocyte/nonmyocyte crosstalk, we cocultured ENPP1 CFs and control CFs with neonatal rat ventricular cardiomyocytes (NRVM) and then added ATP. Within 48 hours of incubation, we observed there was a 75% reduction in the number of ENPP1 CFs with no effect on the number of cardiomyocytes (Figure 2, A and B). In the absence of ATP, there was no reduction in ENPP1 CFs, and ATP did not significantly affect the cell numbers of control CFs cocultured with cardiomyocytes (Figure 2B). In the absence of coculture with cardiomyocytes, addition of ATP did not result in reduction of ENPP1 CFs (Figure 2C). These observations strongly suggest that an interaction among ENPP CFs, ATP, and cardiomyocytes was causing cell death of CFs.

Considering these observations, we hypothesized that the combination of ENPP1 and ATP was inducing the cardiomyocytes to secrete molecules that were causing the death of CFs. To investigate this hypothesis, we added ATP and recombinant ENPP1 protein to NRVM. Following 24 hours of incubation, we collected the conditioned medium and then treated CFs (grown in a separate plate in the absence of any cardiomyocytes) with the myocyte conditioned medium (MCndM) so collected (Figure 2D). Control MCndM included conditioned medium collected from NRVM in an identical manner after treatment with either vehicle, ENPP1, or ATP. Within 48 hours of addition of ENPP1+ATP MCndM, CFs underwent cell death (Figure 2D). Propidium iodide (PI) and annexin V staining with flow cytometry confirmed a significant increase in cell death of CFs treated with ENPP1+ATP MCndM compared with those treated with control MCndM (Figure 2E). TUNEL staining and cleaved caspase-3 activity confirmed the apoptotic cell death of CFs (Figure 2, F and G). To make sure that the immortalization process itself did not make the CFs sensitive to MCndM, we isolated primary CFs, treated them with ENPP1+ATP MCndM, and observed a similar degree of cell death (Supplemental Figure 2, C and D). These observations strongly suggest that in response to ENPP1 protein and ATP, the cardiomyocytes secrete proapoptotic molecules that induce cell death of CFs, but not of myocytes themselves.

To determine whether the ability to secrete proapoptotic molecules was a specific property of cardiomyocytes, we added ENPP1 recombinant protein and ATP to CFs. Transfer of ENPP1+ATP CF–conditioned medium to CFs grown separately in another plate did not cause cell death (Supplemental Figure 2E). These observations thus show that the ability to secrete proapoptotic small molecules in the presence of ENPP1 and ATP is specific to cardiomyocytes. Next, we investigated whether human cardiomyocytes exhibited this property of secreting proapoptotic molecules. We induced cardiomyogenic differentiation of human pluripotent stem cells (hPSCs), treated hPSC-derived cardiomyocytes with ENPP1 and ATP (Supplemental Figure 2F), collected the conditioned medium, added it to human CFs grown in a separate dish, and observed a similar degree of cell death (Supplemental Figure 2, G and H). These observations confirm the ability of human cardiomyocytes to respond to ENPP1 and ATP in a manner similar to that of rodent cardiomyocytes.

We next investigated whether the ectonucleotidase activity of ENPP1 was required for this interaction with cardiomyocytes to generate proapoptotic molecules. We created an expression construct for a mutant ENPP1 (containing a single amino acid substitution in the catalytic domain; ref. 15) that was devoid of nucleotidase activity (Supplemental Figure 2I). We lentivirally overexpressed the mutant ENPP1 construct in CFs and subsequently immortalized them (mutant ENPP1 CFs). When mutant ENPP1 CFs or control ENPP1 CFs were cocultured with NRVM, the addition of ATP induced cell death of ENPP1 CFs, whereas minimal cell death was observed in mutant ENPP1 CFs (Supplemental Figure 2, J and K). These experiments thus demonstrate that the catalytic domain of ENPP1 is necessary for the cardiomyocytes to generate proapoptotic molecules. ENPP1 hydrolyzes extracellular ATP directly into AMP and pyrophosphate (PPi), so if the catalytic domain of ENPP1 is necessary for the myocytes to generate proapoptotic molecules, it follows that either AMP or PPi alone should be able to induce cardiomyocytes to secrete proapoptotic molecules. We added AMP or PPi to cardiomyocytes, collected MCndM after 24 hours, and then added MCndM to CFs grown separately. AMP MCndM caused cell death, but PPi MCndM did not (Figure 2, H and I). However, the addition of AMP alone to CFs did not induce cell death, thus excluding a potential toxic effect of AMP alone on CFs (Supplemental Figure 2L). These observations thus demonstrate that AMP, a product generated by ENPP1-mediated hydrolysis of ATP, induces the myocyte to generate proapoptotic molecules that cause cell death of CFs.

We next investigated the dynamics of cell death and performed quantitative phase microscopy (QPM) to determine the biomass of numerous individual CFs following treatment with MCndM. As cells undergo apoptosis, the surface area and cell biomass of the affected cells decreases (19). We observed that CFs treated with ENPP1+ATP MCndM exhibited an increasing cell biomass with stable cell surface area for the first 12 hours, similarly to CFs treated with control MCndM (Supplemental Figure 2, M and N). After 12 hours, CFs treated with ENPP1+ATP MCndM exhibited a rapid and significant decline in cell biomass and cell surface area due to apoptosis (Supplemental Figure 2, M and N, and Supplemental Video 1). Plots of single-cell surface area versus individual biomass clearly demonstrated significant mean differences in cell size and biomass of CFs after 24 hours of treatment with ENPP1+ATP MCndM versus control MCndM (Supplemental Figure 2, O and P).

In addition to CFs, macrophages and endothelial cells also expressed ENPP1 after cardiac injury. We added ENPP1+ATP MCndM to macrophages, endothelial cells, or smooth muscle cells grown separately and observed that conditioned medium was able to induce cell death in these nonmyocyte populations as well (Figure 2, J and K). However, ENPP1 and ATP MCndM did not induce cell death of cardiomyocytes grown separately (Figure 2L), and neither did it affect myocyte contractility (Supplemental Figure 3, A and B). These observations demonstrate that, in response to ENPP1+ATP, cardiomyocyte-secreted proapoptotic molecules can cause cell death of a wide variety of nonmyocyte cells, but the myocyte itself remains immune to such death-inducing molecules.

### Genetic deletion of ENPP1 leads to enhanced cardiac wound healing.

Before determining the identity of the proapoptotic molecules secreted by myocytes, we investigated the physiological role of ENPP1 in regulating cardiac wound healing in vivo. CFs, macrophages, endothelial cells, and smooth muscle cells play a vital role in cardiac repair after ischemic cardiac injury. We hypothesized that if increased ENPP1 expression in the heart promotes the cardiac muscle cell to secrete molecules that exert adverse effects on nonmyocyte cells, then cardiac repair should be augmented by inhibiting ENPP1. Better preservation of postinjury cardiac function, increased angiogenesis, decreased inflammation, and decreased postinjury scar size were defined by us as quantifiable metrics to characterize cardiac wound healing.

**Figure 3:**
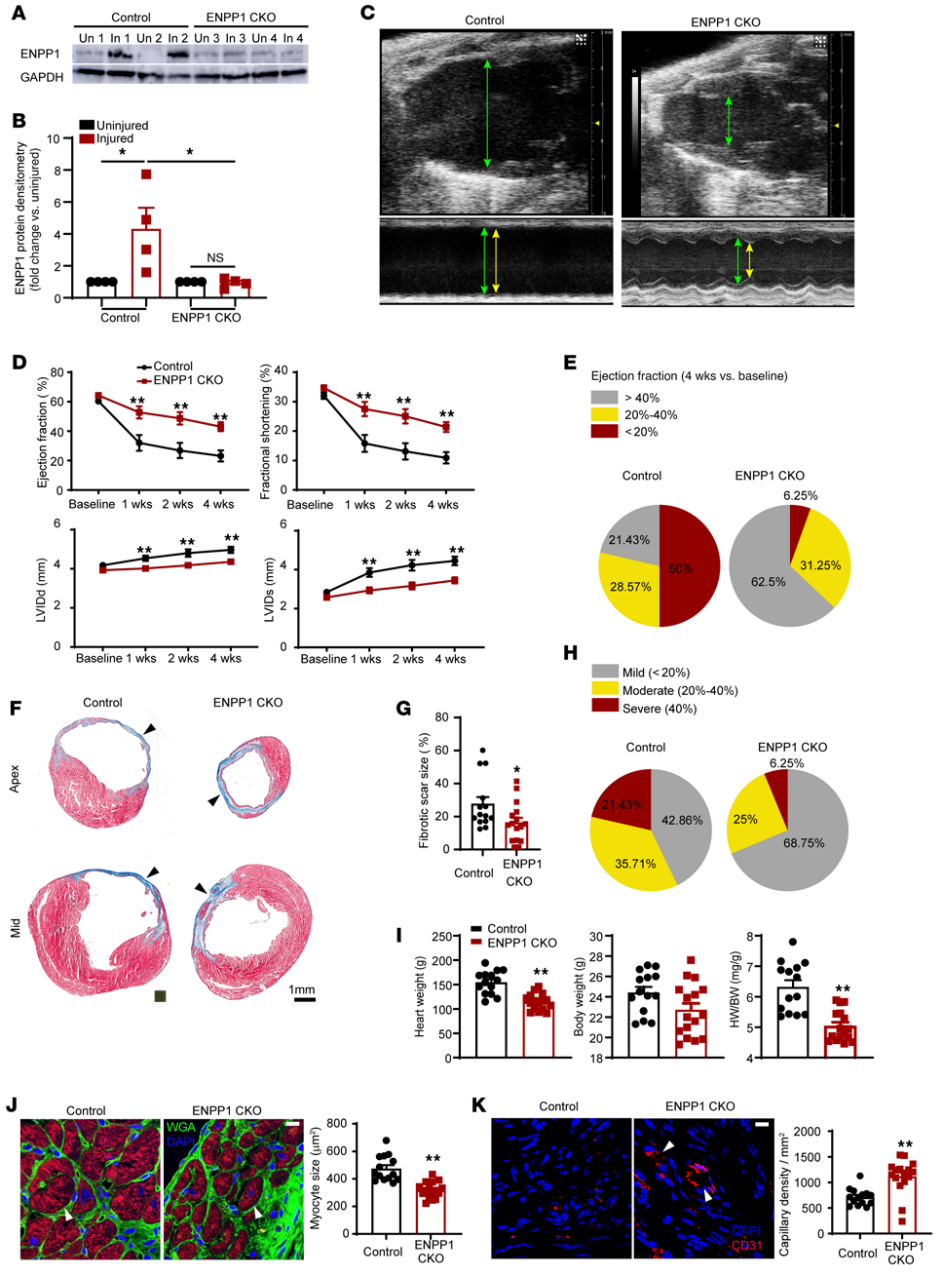
Genetic deletion of ENPP1 leads to enhanced cardiac repair and better preservation of postinjury heart function. (**A**) Western blotting for ENPP1 expression in the hearts of ENPP1CKO animals at 7 days following cardiac injury and (**B**) quantitative densitometry of ENPP1 expression (*n =* 4). (**C**) B mode and M mode echocardiogram demonstrating better contractile function with decreased chamber dilatation at 4 weeks following cardiac injury (green arrows, diastole; yellow arrows, systole). (**D**) EF and fractional shortening as well as LV chamber size (LVID) in systole and diastole over 4 weeks after cardiac injury in control and ENPP1CKO animals. (**E**) Pie chart demonstrating fraction of animals with mild, moderate, and severe reductions in EF. (**F**) Masson trichrome staining demonstrating scar size (blue) measured at the apex and midventricle and (**G**) quantitation of differences in scar size as a fraction of the LV surface area. (**H**) Pie chart showing animals (%) with mild, moderate, and severe fibrosis. (**I**) Heart weight (HW), body weight (BW), and HW/BW ratios measured at 4 weeks following cardiac injury and (**J**) cardiac troponin T immunostaining to determine myocyte surface area (arrowheads) at the border zone and quantitation of myocyte surface area. Scale bar: 10 μm. (**K**) Number of capillaries (CD31 staining, arrowheads) in ENPP1CKO and control animals at 4 weeks after heart injury and quantitation of capillary density. Scale bar: 10 μm. Data are represented as mean ± SEM. **P < 0.01; *P < 0.05, ordinary 1-way ANOVA with Tukey’s multiple comparison test (**B**), ordinary 2-way ANOVA with Šidák’s multiple comparisons test (**D**), or 2-tailed Student’s *t* test (**G**, **I**–**K**). *n =* 14 in control and *n =* 16 in ENPP1CKO animals (**D**, **E**, and **G**– **K**).

As ENPP1 expression significantly overlapped with the expression of Col1a2 in on single-cell RNA-Seq (Supplemental Figure 1G), we used the Col1a2CreERT driver to conditionally delete ENPP1 in CFs. We crossed the Col1a2CreERT animals with animals that had both ENPP1 alleles floxed (ENPP1^fl/fl^; ref. 20), and progeny mice were administered tamoxifen from 5 days prior to cardiac injury to 7 days after to maximize ENPP1 deletion (ENPP1 conditional knockout [ENPP1CKO]). Western blotting confirmed decreased ENPP1 expression in the injured area (Figure 3, A and B). On serial echocardiography, ENPP1CKO animals demonstrated significantly better cardiac function and decreased chamber size within 7 days of injury compared with control littermates (Figure 3, C and D). We defined mild, moderate, or severe depression in postinjury cardiac contractile function as ejection fraction (EF) greater than 40%, between 20% and 40%, and less than 20%, respectively. Only 6% of the animals in the ENPP1CKO group exhibited severe depression in EF compared with almost 50% in control littermates (Figure 3E and Supplemental Table 1A). The degree of fibrosis or scar size measured at 4 weeks after injury was significantly lower in the ENPP1CKO animals (Figure 3, F and G). We again classified the scar size as mild (<20%), moderate (20%–40%), and severe (>40% of left ventricular [LV] surface area) and observed that approximately 21% of the control animals exhibited severe fibrosis at 4 weeks after MI in contrast to less than 6.2% in the ENPP1CKO animals (Figure 3H and Supplemental Table 1B). Postinfarct hypertrophy is an adverse outcome of wound healing (21), and we observed that the heart weight/body weight ratio was significantly lower in the ENPP1CKO animals (Figure 3I). Histology of the peri-infarct area confirmed significantly decreased myocyte surface area in ENPP1CKO hearts (Figure 3J). Capillary density was significantly increased in the ENPP1CKO animals at 4 weeks after injury (Figure 3K). These observations demonstrate that cardiac wound healing is significantly enhanced in ENPP1CKO animals, with better preservation of postinjury function, greater angiogenesis, and significantly decreased scarring and postinjury cardiac hypertrophy.

### Genetic variation of ENPP1 in heart predicts adverse cardiac outcomes across 100 diverse strains of mice.

**Figure 4:**
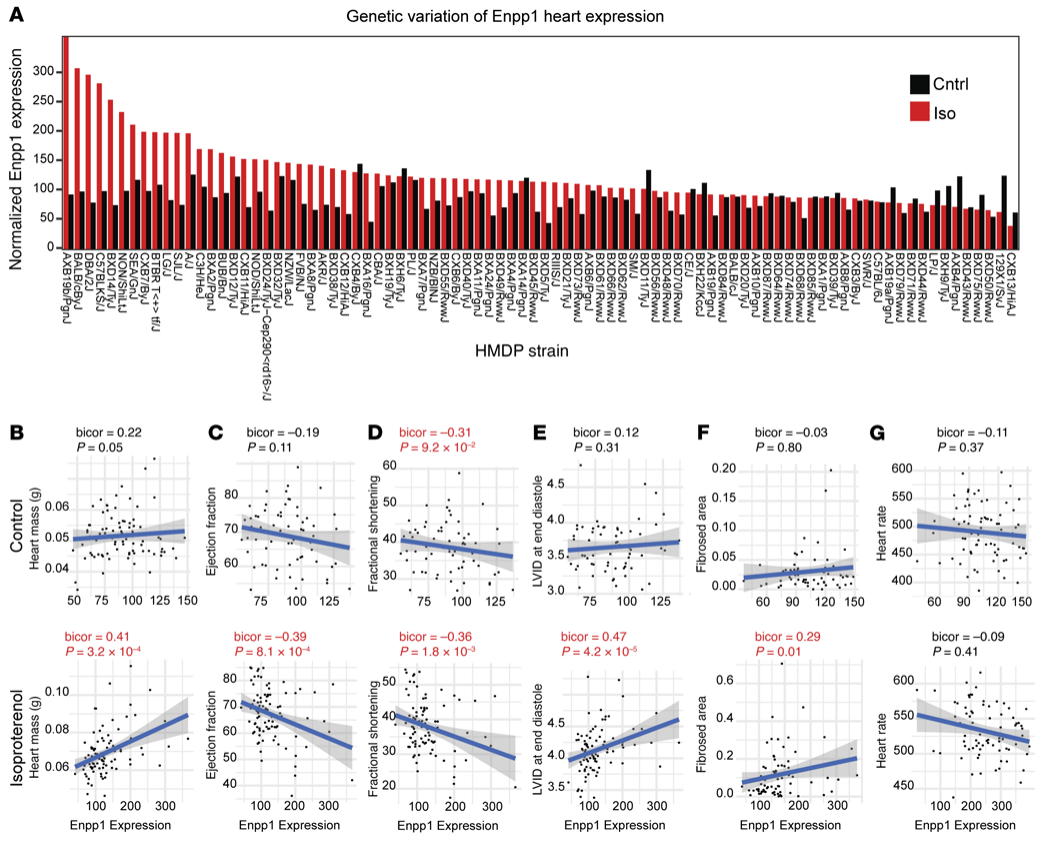
ENPP1 expression across 100 strains of mice correlates with cardiac function in an isoproterenol-induced cardiac injury model. (**A**) Genetic variation of ENPP1 expression across 100 strains of mice following 3 weeks of isoproterenol or saline (control) infusion. (**B**–**F**) Cardiac traits of (**B**) cardiac mass, (**C**) EF, (**D**) fractional shortening, (**E**) LVID (diastole), and (**F**) fibrosis strongly correlating with ENPP1 expression across 100 strains of mice while (**G**) there is an absence of correlation with a trait such as heart rate. Data are shown as scatterplots. bicor, midweight bicorrelation coefficient and corresponding regression Student’s *P* value.

To further strengthen our observations on the role of ENPP1 in regulating cardiac wound healing, we employed a systems genetics approach using alternative models of cardiac injury. The Hybrid Mouse Diversity Panel (HMDP) comprises 100 diverse classical and recombinant inbred strains of mice that can be subjected to cardiac injury to identify genetic determinants of postinjury cardiac traits (22, 23). The mouse strains in the HMDP were treated with 3 weeks of continuous isoproterenol infusion that resulted in cardiomyocyte hypertrophy and interstitial fibrosis. Animals were followed by serial echocardiograms to determine EF and hearts harvested to determine LV gene expression changes. Gene expression signatures were statistically correlated with clinical traits to identify significant relationships across all the strains. Using this system, we initially observed a large degree of genetic variation in ENPP1 expression, particularly following isoproterenol infusion (Figure 4A). Next, we analyzed the association of genes versus traits and observed that ENPP1 expression significantly correlated with the development of adverse postinjury traits such as LV hypertrophy (cardiac mass), increased chamber size, decreased cardiac contractility, and degree of fibrosis following isoproterenol infusion (Figure 4, B–F). As a control, we examined a cardiac phenotype unrelated to wound healing, such as heart rate, and did not see any significant correlation with ENPP1 expression (Figure 4G). These observations using systems genetics approaches provide compelling evidence that ENPP1 is a strong driver of cardiac repair.

### Single-cell RNA-Seq of injured ENPP1CKO hearts demonstrates downregulation of proinflammatory, apoptotic, and fibrotic pathways.

**Figure 5:**
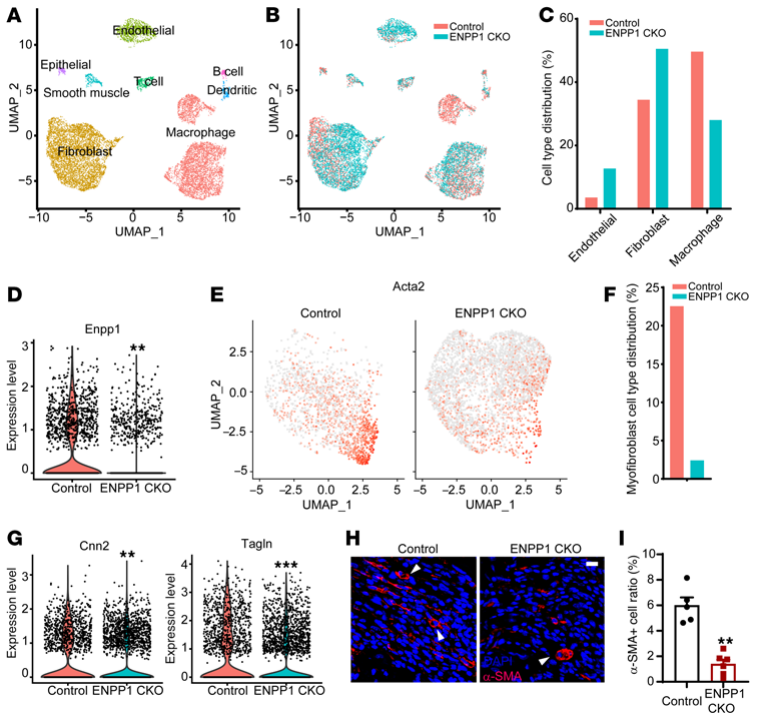
Single-cell RNA-Seq of nonmyocytes in control and ENPP1CKO animals at 7 days following cardiac injury. (**A**) Uniform manifold approximation and projection (UMAP) demonstrating different phenotypes of nonmyocyte cell clusters in the injured heart and (**B**) distribution of WT and ENPP1CKO cells across these clusters. (**C**) Fraction of endothelial cell, fibroblasts, and macrophages at 7 days following injury. Violin plot demonstrating (**D**) ENPP1 expression (**P = 5.29 × 10^–132^) and (**E**) UMAP demonstrating significantly reduced distribution of Acta2 (myofibroblast marker) in CFs of ENPP1CKO versus control animals and (**F**) quantitation (%) of myofibroblasts. (**G**) Violin plot demonstrating decreased expression of other myofibroblast genes Cnn2 (**P = 3.63 × 10^–24^) and Tagln (***P = 5.40 × 10^–17^) in ENPP1CKO fibroblasts. (**H**) Immunostaining for myofibroblasts (αSMA expression, arrowheads) in ENPP1CKO and WT animals and (**I**) quantitation of myofibroblast numbers. Data are represented as mean ± SEM. *n =* 5. **P < 0.01, 2-tailed Student’s *t* test. Scale bar: 10 μm.

To investigate mechanisms of enhanced cardiac repair in ENPP1CKO animals, we performed single-cell RNA-Seq of nonmyocytes in control and ENPP1CKO hearts at 7 days following injury to determine changes in cell-specific transcriptional signatures. We observed fibroblasts, macrophages, and endothelial cells as the largest contributors to the nonmyocyte population (Supplemental Figure 4A and Supplemental Figure 5A). Increased numbers of endothelial cells and fibroblasts were observed in ENPP1CKO hearts, consistent with the proapoptotic effect of ENPP1/AMP-mediated metabolic cascade (Figure 5, A and C). Macrophages were decreased, likely reflecting decreased nonmyocyte cell death and attenuation of the inflammatory response (Figure 5, B and C). Next, we specifically examined the CF population and first confirmed decreased ENPP1 expression (Figure 5D). Myofibroblasts are activated fibroblasts that secrete matrix proteins to form scar tissue, and the number of myofibroblasts, identified by α–smooth muscle actin (αSMA) expression, was decreased in ENPP1CKO hearts (Figure 5, E and F). Other markers of activated myofibroblasts, such as calponin (Cnn2) and transgelin (Tagln) (14), were also decreased (Figure 5G). Immunostaining for αSMA confirmed the decrease in myofibroblasts (Figure 5, H and I).

**Figure 6:**
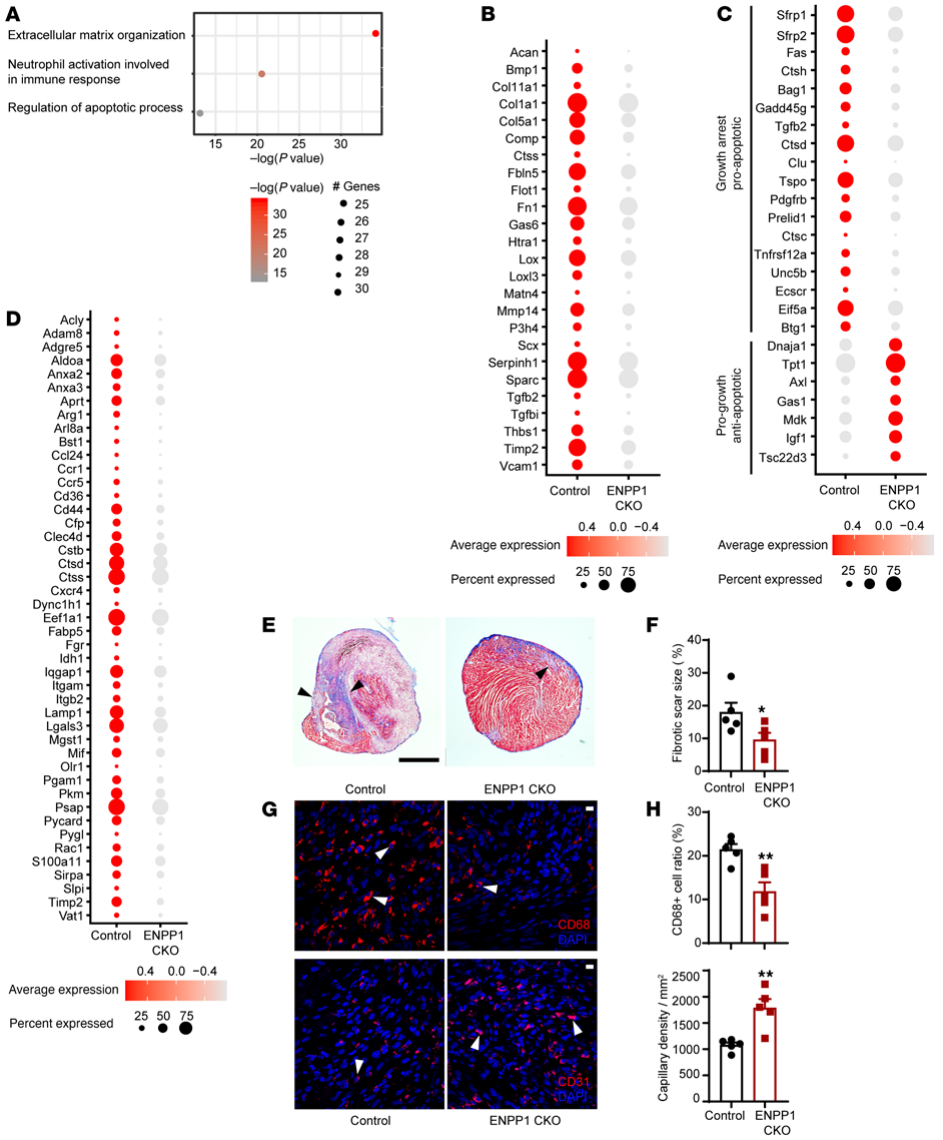
GO analysis of nonmyocyte single-cell RNA-Seq at 7 days following cardiac injury. (**A**) GO analysis demonstrating principal pathways upregulated in CFs of control mice versus ENPP1CKO animals. (**B**) Dot plot demonstrating principal ECM genes upregulated in control versus ENPP1CKO CFs 7 days after cardiac injury. (**C**) Dot plot demonstrating differential expression of pro- and antiapoptotic genes in CFs at 7 days after injury. (**D**) Dot plot demonstrating differential expression of inflammatory genes in macrophages of control and ENPP1CKO hearts at 7 days after injury. (**E**) Masson trichrome staining showing expression of collagen (blue) in the apical region of hearts of mice at 7 days following injury and (**F**) quantitation of fibrotic scar (arrowheads) size at 7 days following injury (*n =* 5). Scale bar: 1 mm. (**G**) Immunofluorescent staining demonstrating CD68 (macrophages, arrowheads) and CD31 (endothelial, arrowheads) staining in control and ENPP1CKO animals at 7 days following injury and (**H**) quantitation of cells by histology (*n =* 5). Scale bars: 10 μm. Data are represented as mean ± SEM. **P < 0.01; *P < 0.05, 2-tailed Student’s *t* test (**F** and **H**).

Gene ontology (GO) demonstrated downregulation of extracellular matrix (ECM) organization and inflammatory and apoptotic pathways in CFs (Figure 6A). Canonical genes known to regulate ECM deposition were significantly downregulated in CFs in ENPP1CKO animals (Figure 6B). Analysis of apoptotic pathways demonstrated downregulation of proapoptotic genes or genes inducing growth arrest and upregulation of antiapoptotic genes in CFs of ENPP1CKO animals (Figure 6C). The average expression of proapoptotic genes (apoptosis module score) was significantly lower in ENPP1CKO CFs (Supplemental Figure 4B), and immunostaining confirmed decreased numbers of apoptotic CFs in ENPP1CKO animals at 3, 7, and 14 days after cardiac injury (Supplemental Figure 4, C and D). The average expression of cell cycling genes (cell-cycle module score) in CFs of ENPP1CKO hearts was higher compared with that in control littermates (Supplemental Figure 4E), and immunostaining confirmed a higher fraction of proliferating CFs in the ENPP1CKO hearts (Supplemental Figure 4, F and G). Taken together, these observations suggest that even though fibroblast numbers are increased with decreased apoptosis of CFs in the ENPP1CKO hearts, myofibroblast activation and expression of canonical ECM genes is significantly decreased. Macrophages in ENPP1CKO hearts also exhibited decreased expression of proinflammatory genes (Figure 6D). Histology at 7 days after injury showed decreased collagen deposition (Figure 6, E and F) along with a significantly decreased number of macrophages and increased number of capillaries (Figure 6, G and H), findings consistent with the RNA-Seq analysis. Immunostaining confirmed significantly decreased numbers of apoptotic endothelial cells in the hearts of ENPP1CKO animals (Supplemental Figure 4, H and I) associated with a higher cell-cycle module score (Supplemental Figure 4J), suggesting that a greater number of endothelial cells in the ENPP1CKO hearts are likely secondary to both increased survival and proliferation. Examination of surviving myocardium with 2,3,5-triphenyltetrazolium chloride (TTC) staining at 24 hours following injury, however, did not demonstrate any differences between the ENPP1CKO and control hearts (Supplemental Figure 4, K and L). Immunostaining confirmed no significant differences in the number of apoptotic myocytes between the control and ENPP1CKO animals (Supplemental Figure 4, M and N), thereby suggesting the beneficial effects on cardiac function are likely secondary to augmented cardiac repair and attenuation of adverse postinfarct remodeling rather than due to a greater amount of surviving myocardium.

Fibrosis after cardiac injury is a complex phenotype regulated by multiple factors, such as the degree of inflammation, the amount of neovascularization or postinfarct angiogenesis, myofibroblast activation, and mechanical properties of the ECM that provide feedback for ECM deposition (14). Even though fibroblast apoptosis was decreased and led to a higher number of cycling fibroblasts in the ENPP1CKO hearts, the degree of myofibroblast activation and overall ECM gene expression was significantly attenuated. ENPP1CKO animals exhibited lesser degrees of endothelial cell apoptosis and greater postinfarct angiogenesis. Decreased inflammation and decreased nonmyocyte cell death could have contributed to decreased activation of fibroblasts and superior cardiac remodeling in ENPP1CKO hearts. Thus, augmented nonmyocyte survival leads to superior tissue repair independently of initial myocyte cell death. Genetic deletion of ENPP1 switches the wound healing transcriptional response after cardiac injury to a more proreparative one with less inflammation, less scarring, and greater angiogenesis.

### Cardiomyocyte-secreted metabolites rather than proteins cause cell death of nonmyocytes.

We next sought to identify the proapoptotic molecules secreted by cardiomyocytes in response to the presence of ATP and ENPP1. We first determined whether the proapoptotic molecules were proteins or metabolites. We collected the ENPP1+ATP MCndM and subjected it to high heat (95°C) for 15 minutes to enable denaturation of proteins (Supplemental Figure 5A). When added to CFs, the heat-treated MCndM retained biological activity and induced CF cell death (Supplemental Figure 5B). To confirm these results, we next passed the ENPP1+ATP MCndM through a protein fractionation column with a filter cutoff of 3 kD (Supplemental Figure 5C) and then treated CFs with the protein-rich (>3 kD) or protein-poor fractions (<3kD) of the MCndM. The protein-rich fraction (MW > 3 kD) did not cause cell death, but the MCndM filtrate at less than 3 kD induced CF cell death (Supplemental Figure 5D). These observations taken together suggest that metabolites rather than proteins secreted by cardiomyocytes cause cell death of nonmyocyte cells.

To determine the identity of the metabolites, we collected MCndM following treatment of the myocytes with ENPP1, ATP, ENPP1+ATP, AMP, or PPi and subjected the conditioned medium to liquid chromatography–mass spectrometry (LC-MS) analysis. We identified metabolites that were differentially present between ENPP1+ATP- or AMP-treated MCndM and control MCndM. These metabolites mainly related to purine or pyrimidine biosynthesis and catabolism pathways and did not include any known proapoptotic factors (Supplemental Figure 5, E and F). We treated CFs with each of the top 7 most differentially upregulated metabolites in the ENPP1+ATP or AMP MCndM, but none of these metabolites caused cell death (Supplemental Figure 5, F and G).

### Death of nonmyocytes is related to cell proliferation.

**Figure 7:**
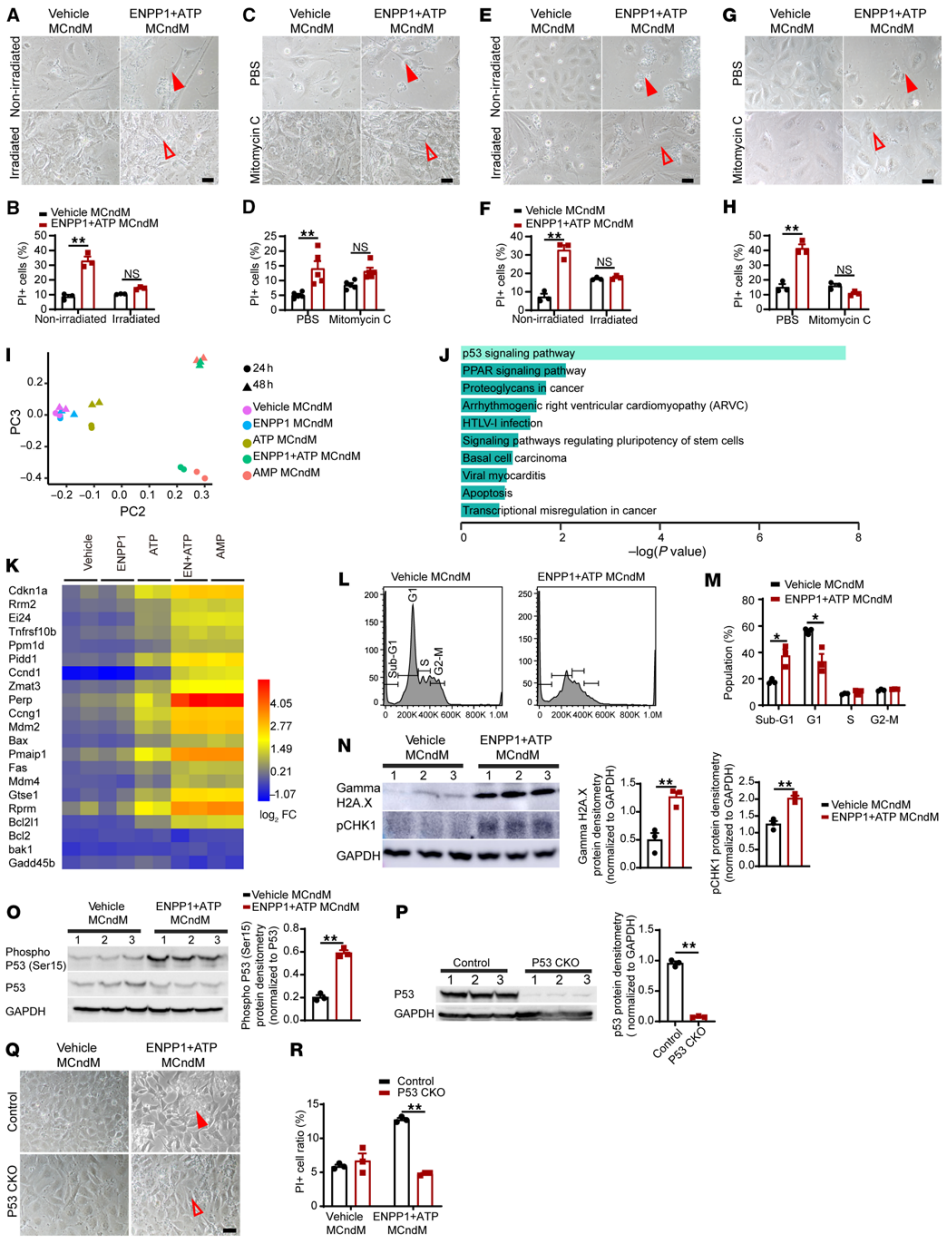
Proapoptotic molecules secreted by cardiomyocytes cause cell death only in cycling cells. (**A**) Irradiated or nonirradiated CFs treated with vehicle or ENPP1+ATP MCndM for 48 hours. Cell death in irradiated but not in irradiated CFs (filled arrow) (**B**) Quantitation of cell death (*n =* 3). (**C**) PBS- or mitomycin C–treated CFs treated with vehicle or ENPP1+ATP MCndM for 48 hours; rescue of cell death with mitomycin (filled and unfilled arrowheads). (**D**) Quantitation of cell death (*n =* 5). Scale bar: 50 μm. (**E**–**H**) MEFs treated with vehicle or ENPP1+ATP MCndM following (**E**) irradiation or (**G**) mitomycin C. Rescue of cell death with irradiation or mitomycin C (filled and unfilled arrowheads) and (**F** and **H**) quantification of cell death following (**F**) irradiation (*n =* 3) or (**H**) mitomycin treatment (*n =* 3). Scale bars: 50 μm. (**I**) Principal component analysis of gene expression changes in CFs treated with ENPP1+ATP MCndM (*n =* 2). (**J**) GO analysis of main differentially expressed pathways in CFs following treatment with ENPP1+ATP MCndM. (**K**) Heatmap demonstrating expression of p53-driven apoptotic genes in CFs treated with ENPP1+ATP MCndM. (**L**) Cell-cycle analysis demonstrating G_1_/S phase arrest in CFs treated with ENPP1+ATP MCndM and (**M**) cells (%) in different phases of cell cycle (*n =* 3). (**N**) Western blot and quantitation for pH2A.X and pCHK-1 in CFs treated with ENPP1+ATP MCndM (*n =* 3). (**O**) Western blot and densitometry of p53 Ser15 phosphorylation in CFs treated with ENPP1+ ATP MCndM (*n =* 3). (**P**) p53 protein levels in p53CKO CFs (*n =* 3) (**Q**) p53CKO CFs treated with vehicle or ENPP1 MCndM demonstrating rescue of cell death in p53CKO CFs (filled and unfilled arrowheads) and (**R**) quantitation of cell death (*n =* 3). Scale bar: 50 μm. Data are represented as mean ± SEM. **P < 0.01; *P < 0.05, 2-tailed Student’s *t* test (**B**, **D**, **F**, **H**, **M**–**P**, and **R**).

Unable to readily identity the proapoptotic metabolites, we sought alternative physiologic principles for approaching the problem. We hypothesized that the ability of myocytes to be immune to the conditioned medium likely reflects an inherent cellular property of myocytes that distinguishes itself from that in nonmyocyte cells. As nonmyocytes are proliferative and myocytes are nonproliferative, we hypothesized that the ability to cycle was making the nonmyocytes susceptible to the metabolites secreted by the myocytes. To determine whether this was true, we irradiated CFs and observed that the ENPP1+ATP MCndM could not induce death of irradiated CFs, in contrast to nonradiated control CFs (Figure 7, A and B). To confirm this finding, we next treated CFs with the cell-cycle inhibitor mitomycin C and again observed cell-cycle–arrested CFs were resistant to ENPP1+ATP MCndM–induced cell death (Figure 7, C and D). To obtain an independent confirmation on the cell-cycle–dependent cell death of CFs treated with ENPP1+ATP MCndM, we repeated the experiments with primary mouse embryonic fibroblasts (mEFs) and confirmed that cell-cycle arrest with irradiation or mitomycin C prevented cell death of mEFs (Figure 7, E–H). These observations suggest that cell cycling of the target nonmyocyte cells is necessary for the myocyte-secreted molecules to cause cell death.

We next examined the mechanisms of cell death secondary to cell cycling. We performed RNA-Seq on CFs treated with ENPP1+ATP MCndM. Principal component analysis demonstrated that the gene expression signatures of CFs treated with ENPP1+ATP or AMP MCndM were similar and clearly distinguishable from those of the other groups (Figure 7I). GO analysis demonstrated significant upregulation of the p53-signaling pathway (Figure 7J) with significant differential expression of p53-regulated proapoptotic genes (Figure 7K), including canonical apoptotic modulators such as Bax, Bak, and BCl2 (Figure 7K). Myocytes remained immune to ENPP1+ATP MCndM and did not exhibit changes in p53-driven apoptosis genes (Supplemental Figure 3C). As cell death was related to cycling, we next examined in detail the phases of cell cycle that were disrupted in nonmyocytes treated with ENPP1+ATP MCndM. CFs demonstrated evidence of G_1_/S phase arrest (Figure 7, L and M). Western blotting demonstrated significant upregulation of DNA damage-response markers (γH2A.X) and the checkpoint kinase 1 (pCHK1) that is known to regulate a DNA damage response and cell-cycle arrest (Figure 7N). Phosphorylation of serine 15 in p53 has been shown to initiate DNA damage response (24, 25), and we observed increased phosphorylation of p53Ser15 in CFs treated with ENPP1+ATP MCndM (Figure 7O). To determine whether a p53-initiated DNA damage response was required for cell death, we deleted the p53 gene in CFs by infecting primary CFs isolated from p53 floxed mice (26) with a lentiviral Cre (Figure 7P). CFs deficient in p53 were resistant to ENPP1+ATP MCndM–induced cell death (Figure 7, Q and R). These experiments thus demonstrate that metabolite or metabolites secreted by the cardiac muscle cell in response to ENPP1 and ATP initiate a p53-dependent DNA damage response and apoptosis in cycling nonmyocyte cells.

### Myocyte-secreted metabolite or metabolites disrupt pyrimidine biosynthesis in cycling nonmyocytes to cause cell death.

**Figure 8:**
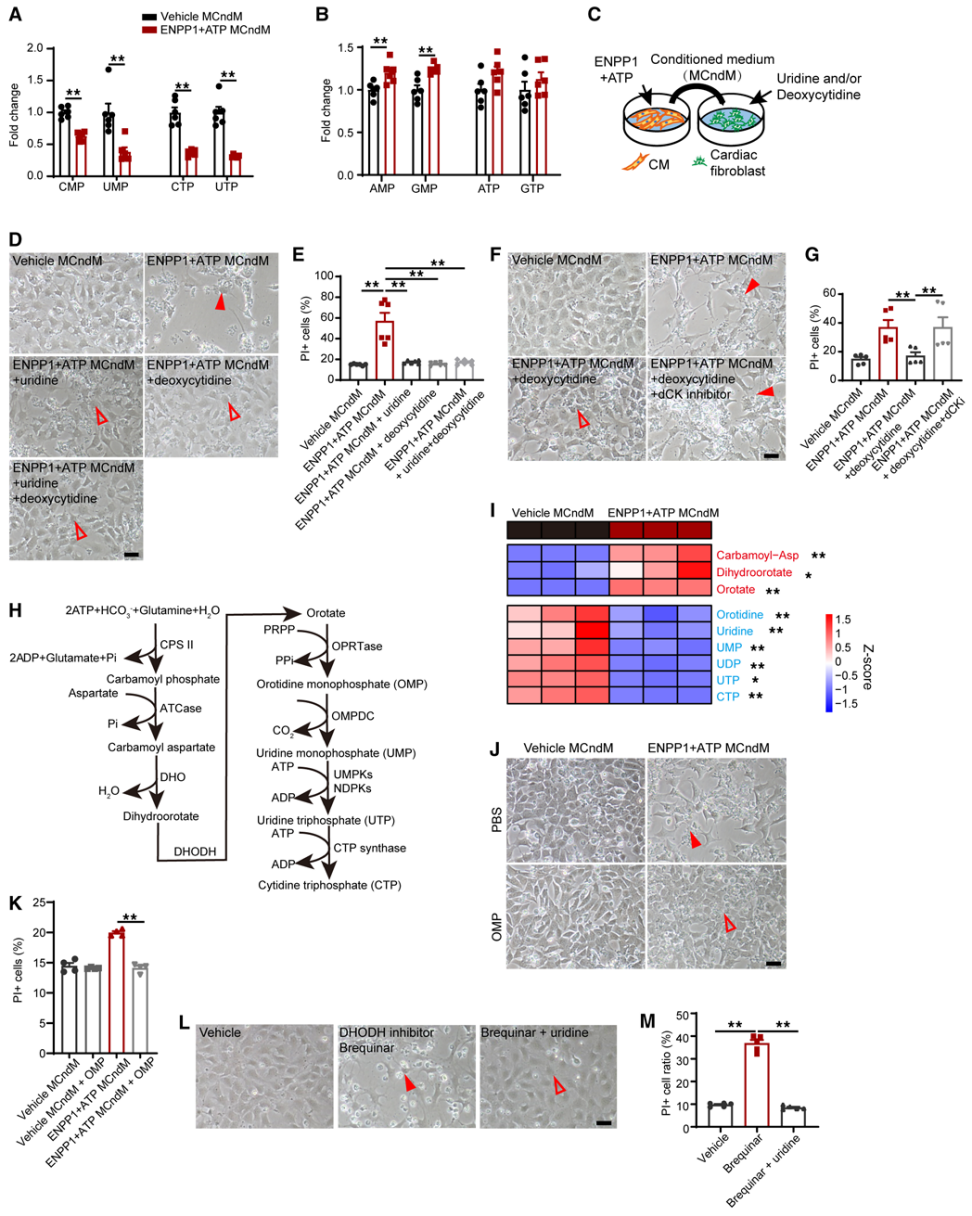
CFs treated with ENPP1+ATP (MCndM) exhibit decreased pyrimidine levels. (**A**) LC/MS-MS demonstrating decreased levels of intracellular pyrimidine nucleotides but not (**B**) purine nucleotides in CFs treated with ENPP1+ATP MCndM (*n =* 6). (**C**) CFs treated with vehicle MCndM or ENPP1+ATP MCndM in the presence of uridine, deoxycytidine, or both demonstrating (**D**) cell death (arrowheads) in CFs treated with ENPP1+ATP MCndM, but rescue of cell death (unfilled arrowheads) following addition of uridine, deoxycytidine, or both. Scale bar: 50 μm. (**E**) Flow cytometry to demonstrate effects on cell death following addition of uridine or deoxycytidine to CFs treated with ENPP1+ATP MCndM (*n =* 6). (**F**) Effect of adding deoxycytidine and deoxycytidine kinase inhibitor (dCKi) to CFs treated with ENPP1+ATP MCndM is a loss of rescue of deoxycytidine in the presence of dCKi (unfilled and filled arrowheads) and (**G**) quantitation of cell death (*n =* 5). Scale bar: 50 μm. (**H**) Outline of critical steps of pyrimidine biosynthesis. (**I**) Heatmap demonstrating differential expression of metabolites in pyrimidine biosynthetic pathway between CFs treated with vehicle MCndM and those treated with ENPP1+ATP MCndM (*n =* 3). (**J**) Rescue of cell death following addition of OMP to CFs treated with ENPP1+ATP MCndM (filled and unfilled arrowheads) and (**K**) quantitation of cell death (*n =* 4). Scale bar: 50 μm. (**L**) Effect on cell death following addition of DHODH inhibitor brequinar (filled arrowheads) to disrupt pyrimidine biosynthesis and rescue with uridine (unfilled arrowheads). (**M**) Flow cytometry to determine effects of brequinar on cell death and rescue by uridine (*n =* 5). Scale bar: 50 μm. Data are represented as mean ± SEM.**P < 0.01; *P < 0.05, 2-tailed Student’s *t* test, (**B**, **I**) or ordinary 1-way ANOVA with Tukey’s multiple comparison test (**E**, **G**, **K**, and **M**).

The experiments related to the ability of ENPP1+ATP MCndM to initiate a DNA damage response in cycling nonmyocyte cells strongly suggested that the metabolite or metabolites interfere with the cell-cycle machinery. A nucleotide balance between the content of purines and pyrimidines available to cycling cells is critical for avoiding genotoxic stress and maintain genomic stability (27). We hypothesized that disruption of the nucleotide biosynthetic pathways in nonmyocyte cells and an imbalance of purine versus pyrimidine nucleotides was inducing genotoxic stress and initiating a DNA damage response in proliferating nonmyocytes. We treated CFs with ENPP1+ATP MCndM for 24 hours and measured the content of nucleoside monophosphate and triphosphates by LC/MS-MS. Consistent with our hypothesis, we observed that the pyrimidines cytidine and uridine mono and triphosphates (CMP, cytidine triphosphate [CTP], uridine monophosphate [UMP], uridine triphosphate [UTP]) were significantly reduced in ENPP1+ATP MCndM–treated CFs (Figure 8A) while purine nucleotide levels were slightly increased or remained unaltered (Figure 8B). These observations suggested that insufficient pyrimidine precursors during cell cycling were likely leading to a DNA damage response. We next attempted to rescue cell death of CFs by adding uridine or deoxycytidine to CFs treated with ENPP1+ATP MCndM (Figure 8C) and observed that addition of uridine or deoxycytidine or both completely rescued cell death (Figure 8, D and E). Deoxycytidine serves as a precursor of dCTP synthesis via the enzyme deoxycytidine kinase. When we added a specific inhibitor of deoxycytidine kinase (DI-87), deoxycytidine was unable to prevent cell death, thereby strongly supporting the hypothesis that pyrimidine deficiency was causing cell death (Figure 8, F and G).

### Inhibition of UMP synthase step is the underlying cause of defects in pyrimidine biosynthesis.

Pyrimidine biosynthesis occurs via a sequence of well-regulated steps in which dihydroorotate formed from carbamoyl phosphate gives rise to orotate and subsequently the important intermediate orotidine monophosphate (OMP) (Figure 8H). To determine which steps in pyrimidine biosynthesis are affected in cycling nonmyocytes, we treated CFs with ENPP1+ATP MCndM for 24 hours and subjected the CFs to mass spectrometry to determine metabolites in the pyrimidine biosynthesis pathway. CFs treated with ENPP1+ATP MCndM showed significantly increased amounts of carbamoyl aspartate, dihydroorotate, and orotate, but decreased orotidine, uridine, UMP, UDP, and UTP as well as CTP (Figure 8I). This suggests that the ENPP1+ATP MCndM inhibits later steps of UMP synthesis in CFs. As orotate levels were increased, but orotidine levels decreased in ENPP1+ATP MCndM–treated CFs, we hypothesized that inhibition was occurring at the OMP synthesis step from orotate and phosphoribosyl pyrophosphate (PRPP). OMP added to CFs completely rescued cell death (Figure 8, J and K), strongly suggesting that ENPP1+ATP MCndM inhibits pyrimidine biosynthesis at the OMP synthesis step in cycling nonmyocytes. Finally, to demonstrate that defects in pyrimidine biosynthesis are sufficient to cause cell death, we treated CFs with a specific inhibitor of dihydroorotate dehydrogenase (DHODH) (brequinar) and observed cell death, thereby demonstrating that disruption in pyrimidine biosynthesis is sufficient to cause cell death (Figure 8, L and M).

### Adenine is a critical proapoptotic metabolite secreted by cardiomyocytes.

**Figure 9:**
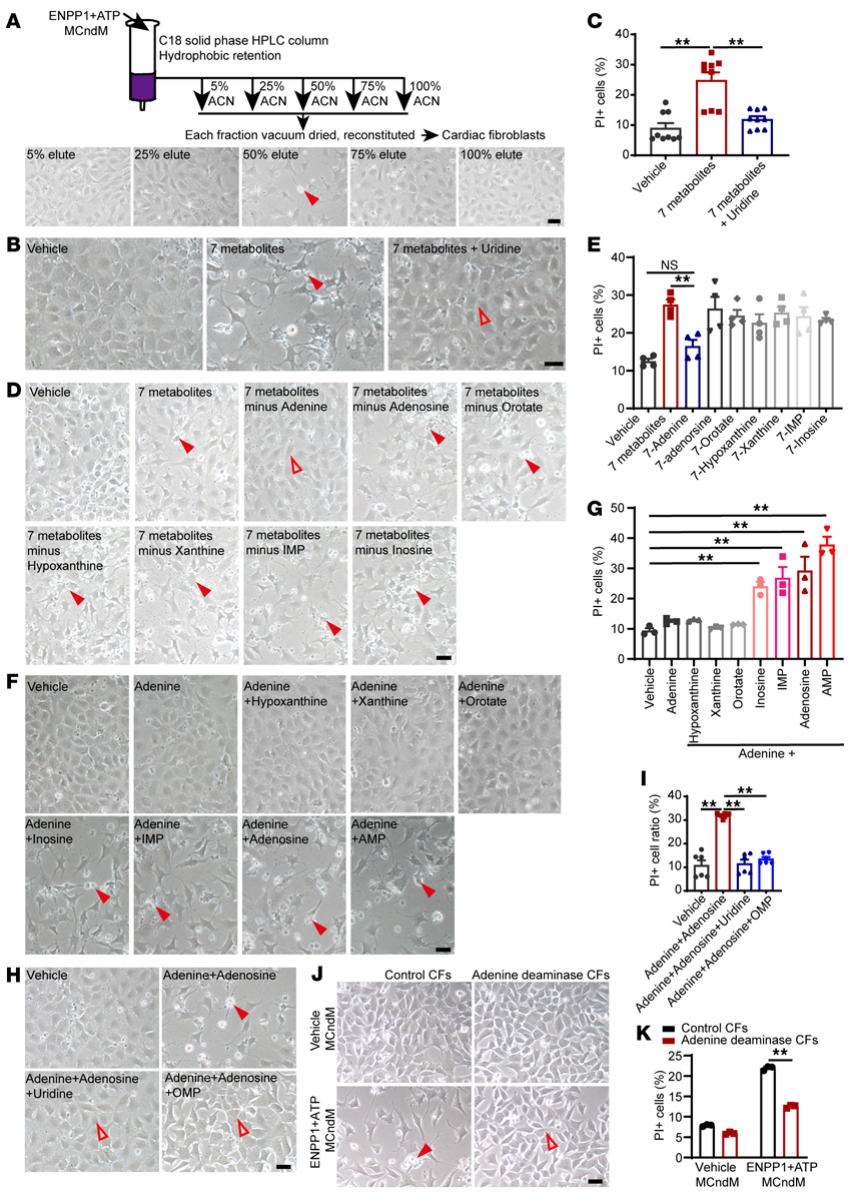
Adenine and specific purine ribonucleosides are myocyte-derived proapoptotic molecules that in combination cause cell death of nonmyocytes. (**A**) Schematic shows ENPP1+ATP MCndM pass through the C18 HPLC column. Effects of eluted fractions of ENPP1+ATM MCndM on cell death (arrowheads) of CFs. Scale bar: 50 μm. (**B**) Effect of 7 metabolites on cell death (filled arrowheads) of CFs and its rescue (unfilled arrowheads) by uridine and (**C**) quantitation of cell death (*n =* 9). Scale bar: 50 μm. (**D**) CFs treated with 7 compounds together and following subtraction of each one from the combined solution show absence of cell death (unfilled arrowheads) when adenine is removed. Cell death, filled arrowheads. Scale bar: 50 μm. (**E**) Quantitation of cell death (*n =* 4). (**F**) Effects of cell death (filled arrowheads) following addition of adenine alone or adenine combined with specific purine nucleosides or orotate and (**G**) quantitation of cell death (*n =* 3). Scale bar: 50 μm. (**H**) Effect of OMP or uridine in rescuing cell death following addition of adenine and adenosine to CFs (filled and unfilled arrowheads) and (**I**) quantitation of cell death (*n =* 6). Scale bar: 50 μm. (**J**) CFs overexpressing yeast adenine deaminase treated with vehicle MCndM or ENPP1+ATP MCndM showing decreased cell death of CFs overexpressing adenine deaminase (filled and unfilled arrowheads). (**K**) Quantitation of cell death (*n =* 3). Scale bar: 50 μm. Data are represented as mean ± SEM. **P < 0.01; *P < 0.05, 2-tailed Student’s *t* test (**K**) or ordinary 1-way ANOVA with Tukey’s multiple comparison test (**C**, **E**, **G**, and **I**).

We returned to the central question of the identity of cardiomyocyte-derived proapoptotic metabolites. As the metabolites inhibited pyrimidine biosynthesis, we hypothesized that the metabolites were likely nucleotides or their derivatives. We performed HPLC to determine physicochemical properties of the candidates. We joined 2 reverse-phase HPLC columns in series, loaded ENPP1+ATP MCndM onto the columns, and used a linear gradient of acetonitrile (ACN) to collect 80 fractions (Supplemental Figure 6A). Pools of 10 fractions were vacuum dried, reconstituted, and added to CFs, and we observed that fractions 41 to 50 from ENPP1+ATP MCndM (corresponding to ACN concentrations of 40% to 50%) reliably resulted in CF cell death, while those of control MCndM did not (Supplemental Figure 6, B and C). To confirm these observations, we loaded a C18 stationary phase column with ENPP1+ATP MCndM, eluted fractions in a similar manner, and observed cell death only with the 50% ACN fraction (Figure 9A and Supplemental Figure 6D). We subjected the 50% ACN eluates of ENPP1+ATP and control MCndM to LC-MS analysis, focusing on nucleosides, nucleotides, and their derivatives. We crosschecked this list with the MS data on the unfractionated ENPP1+ATP-conditioned medium to ensure that the compounds were present in the unfractionated ENPP1+ATP MCndM. We chose 7 compounds that were highly enriched in the 50% ACN eluates of the ENPP1+ATP MCndM (Table 1). Addition of all 7 compounds to CFs caused severe cell death (Figure 9, B and C), but addition of uridine together with the 7 compounds rescued cell death (Figure 9, B and C). This suggested that the mechanism of cell death following addition of the 7 selected compounds was similar to that mediated by ENPP1+ATP MCndM. Next, we subtracted each compound from the set of 7 compounds to determine which compound was necessary for cell death (Figure 9D). We observed that adenine was the only compound critically necessary for cell death, as removal of adenine resulted in rescue of cell death (Figure 9, D and E). However, when CFs were treated with adenine alone, no cell death was observed, demonstrating that adenine, though necessary, was not sufficient for cell death (Figure 9, F and G). We next added adenine plus one of the other 6 compounds and observed that addition of adenine with either adenosine, inosine, inosine monophosphate (IMP), or AMP was sufficient to cause death (Figure 9, F and G). Combinations of adenine with either hypoxanthine, xanthine, or orotate did not cause cell death (Figure 9, F and G). Finally, addition of OMP or uridine rescued cell death of CFs treated with adenine and adenosine (Figure 9, H and I). Taken together, these observations demonstrate that the combination of adenine and a purine nucleoside is sufficient to cause disruption of pyrimidine biosynthesis and induce cell death of nonmyocytes and that such cell death can be rescued with pyrimidine supplementation. Addition of adenine and adenosine to macrophages, endothelial cells, and smooth muscle cells also induced cell death, demonstrating that a combination of adenine and adenosine could induce cell death on a wide variety of nonmyocyte cells (Supplemental Figure 6, E and F).

Next, we wanted to determine whether adenine was a key critical component of the ENPP1+ATP MCndM that induced cell death of nonmyocytes. We adopted a loss-of-function approach to determine whether catabolic removal of adenine would rescue ENPP1+ATP MCndM from causing CF cell death. There is no mammalian enzyme that catabolizes adenine, but plants and microorganisms express adenine deaminase, which converts adenine to hypoxanthine (28). We lentivirally expressed yeast adenine deaminase in CFs and immortalized them to create a stable cell line. CFs expressing adenine deaminase were resistant to ENPP1+ATP MCndM–induced cell death (Figure 9, J and K). These experiments thus conclusively demonstrate that adenine is the key molecule present in ENPP1+ATP MCndM that causes cell death of nonmyocytes.

Our data suggest that pyrimidine synthesis is disrupted at the OMP synthesis step. We next investigated potential reasons for the inhibition of OMP synthesis. PRPP is the donor of phospho-ribose groups for OMP synthesis from orotate as well as in the purine salvage pathway to synthesize AMP from adenine. PRPP synthesis by PRPP synthetase is potently inhibited by AMP and ADP (29). The model illustrated in our findings suggests that the toxicity of the combination of adenine and adenosine could be related to inhibition of PRPP synthetase by AMP, with concomitant consumption of PRPP by adenine catalyzed by adenine phosphoribosyl transferase (APRT). If so, PRPP levels should be significantly reduced, and we observed significantly decreased PRPP levels in CFs treated with ENPP1+ATP MCndM (Supplemental Figure 6G) along with decreased levels of metabolites that are generated using PRPP as a substrate with corresponding increase in nicotinamide (Supplemental Figure 6H).

### AMP and not adenosine induces cardiomyocytes to generate adenine.

AMP, a metabolite generated by the hydrolytic activity of ENPP1 on ATP, was able to induce the myocyte to secrete proapoptotic metabolites. Having identified that adenine is a critical mediator of nonmyocyte cell death, we next investigated whether AMP or its metabolite adenosine is needed for the myocyte to secrete proapoptotic molecules. Extracellular AMP is hydrolyzed by CD73, a membrane-bound protein, to form adenosine. We added AMP to cardiomyocytes in the presence or absence of a CD73 inhibitor, collected the MCndM, and subsequently added it to CFs (Supplemental Figure 7A). We observed that inhibition of CD73 (using 2 different inhibitors, AB680 and AMP-CP) significantly increased CF cell death, strongly suggesting that AMP and not adenosine is necessary for the cardiomyocytes to secrete proapoptotic molecules (Supplemental Figure 7, A and B). Conditioned medium from adenosine-treated cardiomyocytes (adenosine MCndM) was also unable to induce cell death of CFs (Supplemental Figure 7, C and D). Moreover, MCndM collected after the addition of the adenosine receptor agonist 5′-*N*-ethylcarboxamide adenosine (NECA) did not cause fibroblast cell death (Supplemental Figure 7E). Next, we added ENPP1+ATP to cardiomyocytes in the presence of adenosine receptor antagonists and did not observe any change in the ability of ENPP1+ATP MCndM to cause death of CFs (Supplemental Figure 7F), corroborating the observation that adenosine did not induce cardiomyocytes to secrete proapoptotic molecules. Adenosine receptor antagonists also did not affect the ability of cardiomyocytes to remain immune to the ENPP1+ATP MCndM or a combination of adenine and adenosine (Supplemental Figure 3, D–G). Finally, we added an adenosine kinase inhibitor (ABT-702) or AMP deaminase inhibitor (cpd3) to cardiomyocytes at the time of addition of AMP. Adenosine kinase inhibitors would be expected to decrease the generation of AMP from adenosine, while AMP deaminase inhibitors would be expected to increase AMP concentrations by inhibiting AMP deamination. We observed that adenosine kinase inhibitors attenuated cell death of CFs (Supplemental Figure 7, G and H), while AMP deaminase inhibitors worsened cell death (Supplemental Figure 7, I and J), demonstrating that AMP was critically required by the cardiomyocytes to generate proapoptotic molecules.

As cardiomyocyte-derived adenine was necessary for nonmyocyte cell death, we investigated whether AMP was directly utilized by the cardiac muscle cell for adenine synthesis. For this purpose, we added ENPP1^+^ N15-labeled ATP (Supplemental Figure 8A) to cardiomyocytes and treated CFs with the collected MCndM (Supplemental Figure 8B). We then harvested cardiomyocytes treated with ENPP1+N15-labeled ATP and collected the conditioned medium as well as CFs to determine the fraction of adenine and other key metabolites that bear the isotope label (Supplemental Figure 8B). The fraction of N15-labeled adenine in the cardiomyocytes was 77% of the total adenine present, while 98% of the adenine in the conditioned medium and 82% of the adenine in the CFs were labeled (Supplemental Figure 8C). The labeled adenine in the cardiomyocytes contained five 15N atoms, demonstrating direct conversion of 15N5 AMP to adenine by the cardiomyocyte (Supplemental Figure 8D). Also, the majority of the adenine in the conditioned medium had all 5 nitrogen atoms labeled, demonstrating that adenine synthesized by cardiomyocytes directly from AMP is the predominant source of adenine in conditioned medium (Supplemental Figure 8E). Similarly, the majority of the adenosine, IMP, inosine, and AMP in the cardiomyocytes, cardiomyocyte-conditioned medium, and the CFs was labeled (Supplemental Figure 8F). The fraction of labeled nucleoside guanosine was much lower in cardiomyocytes, while in the CFs, almost 60% of guanosine was labeled (Supplemental Figure 8G). All 4 nitrogens in the purine ring of guanosine were 15N, suggesting it was derived from the 15N5 AMP (Supplemental Figure 8H). Labeling of unrelated metabolites, such as glutamate, not typically derived from adenosine derivatives was expectedly low, demonstrating the fidelity of our system as a negative labeling example (Supplemental Figure 8I). Taken together, these observations demonstrate that AMP formed by ENPP1-mediated hydrolysis of ATP is directly metabolized by cardiomyocytes to generate adenine and other nucleosides that are then released extracellularly to exert biological effects.

### Uridine administration after heart injury augments cardiac repair and function in vivo.

**Figure 10:**
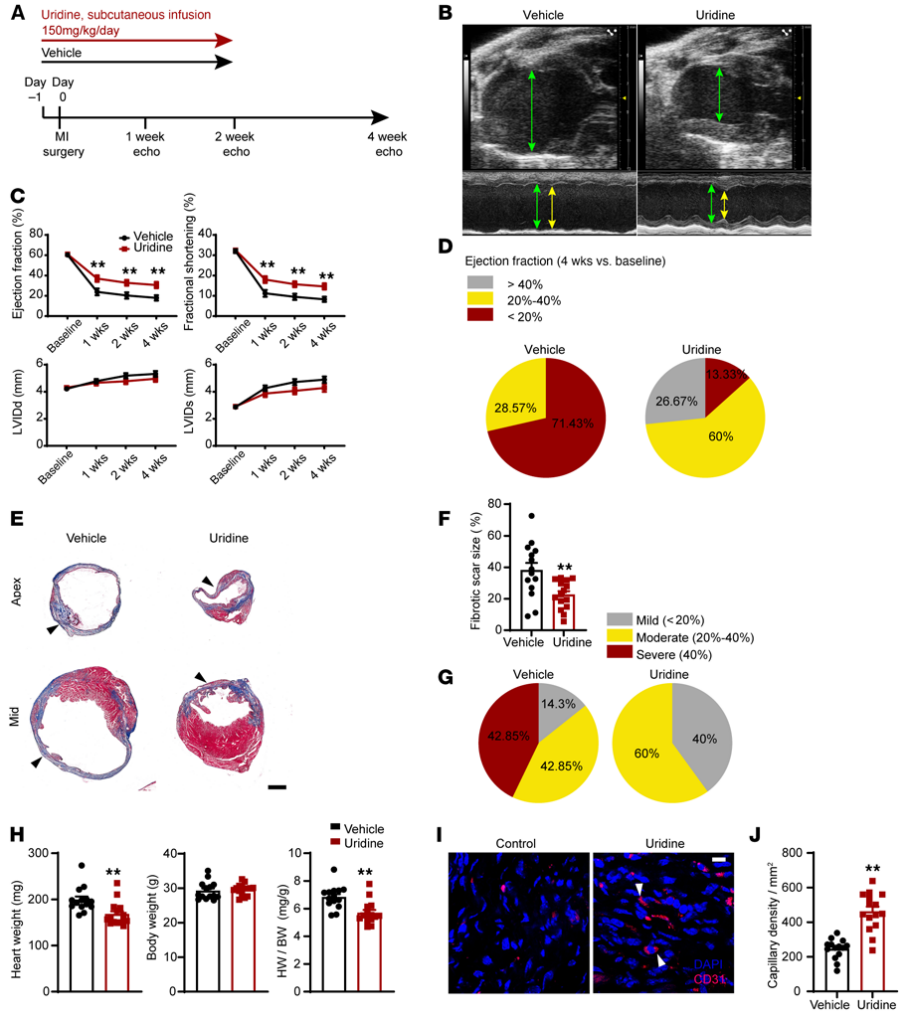
Pyrimidine supplementation with systemic uridine administration is associated with significantly better cardiac repair and postinjury heart function. (**A**) Schematic of continuous uridine administration by a subcutaneous pump starting 1 day prior to injury and continuing for 14 days after. (**B**) B and M mode echocardiogram demonstrating better preservation of contractile function during diastole (green arrows) and systole (yellow arrows) in uridine-injected animals. (**C**) EF and fractional shortening and LV internal diameter in systole and diastole following uridine administration. *n =* 15 (vehicle) and *n =* 15 (uridine) at basal, 1 week, 2 weeks, and 3 weeks; *n =* 14 (vehicle) and *n =* 15 (uridine) at 4 weeks. (**D**) Pie chart demonstrating fraction of animals with mild, moderate, and severe reductions in EF following vehicle or uridine administration. (**E**) Masson trichrome staining demonstrating scar size (blue) at apex and midventricles of vehicle- or uridine-injected animals and (**F**) quantitation of differences in scar size as a fraction of the LV surface area. *n =* 14 (vehicle) and 15 (uridine). Scale bar: 1 mm. (**G**) Pie chart demonstrating fraction of animals demonstrating mild, moderate, and severe fibrosis following vehicle or uridine administration, (**H**) Heart weight, body weight, and HW/BW ratio in vehicle- versus uridine-treated animals. *n =* 14 (vehicle) and *n =* 15 (uridine). (**I**) Histology demonstrating capillaries (CD31 staining) in injured regions of hearts 4 weeks after injury in vehicle- or uridine-treated animals and (**J**) quantitation of capillaries. *n =* 14 (vehicle) and 15 (uridine). Scale bar: 10 μm. Data are represented as mean ± SEM. **P < 0.01, ordinary 2-way ANOVA with Šidák’s multiple comparisons test (**C**) or 2-tailed Student’s *t* test (**F**, **H**, and **J**).

Uridine supplementation to nonmyocytes treated with ENPP1+ATP MCndM rescued cell death. We hypothesized that if ENPP1 worsened cardiac repair by inducing defects in pyrimidine biosynthesis in vivo, then administration of uridine should rescue pyrimidine biosynthesis and lead to better preservation of postinjury cardiac function. We subjected WT C57BL/6 animals to ischemic cardiac injury and administered uridine by continuous infusion for 14 days starting from the day of injury (Figure 10A). Echocardiography demonstrated that uridine significantly preserved postinjury cardiac contractile function, with a trend toward better preservation of ventricular diameters (Figure 10, B and C). We observed that 71% of the vehicle-injected animals exhibited severe depression in EF compared with only 13% of the animals that received uridine (Figure 10D and Supplemental Table 1A). Histology demonstrated decreased fibrosis in uridine-injected animals (Figure 10, E and F). Approximately 43% of the animals in the vehicle group exhibited severe postinfarct fibrosis, but none of the animals that received uridine exhibited severe fibrosis (Figure 10G and Supplemental Table 1B). Hearts of uridine-injected animals exhibited decreased heart weight/body weight ratios (Figure 10H) and significantly increased capillary density (Figure 10, I and J). These data taken together provide proof of concept that rescue of pyrimidine biosynthesis with pyrimidine supplementation in the injured heart represents a therapeutic strategy for heart repair.

### ENPP1 inhibitors as therapeutic agents to enhance wound healing after cardiac injury.

As ENPP1 was the principal nucleotidase in the injured heart, we hypothesized that ENPP1 could serve as a therapeutic target for augmenting cardiac wound healing following ischemic injury. We established a luminescent assay to screen a large library of 200,000 compounds at our institution to identify small molecule inhibitors of ENPP1. Myricetin, a polyphenolic flavonoid (30), was the leading hit, with almost 90% inhibition of ENPP1 enzymatic activity and an IC_50_ of 4.8 μM (Supplemental Figure 9, A and B). Addition of ATP induced cell death of ENPP1-overexpressing CFs cocultured with NRVM, but concomitant addition of myricetin (10 μM) significantly attenuated cell death (Supplemental Figure 9, C and D). Next, we treated cardiomyocytes with ENPP1+ATP and myricetin, collected the MCndM, added it to CFs, and observed a significant reduction in cell death compared with using ENPP1+ATP MCndM (Supplemental Figure 9, E and F).

**Figure 11:**
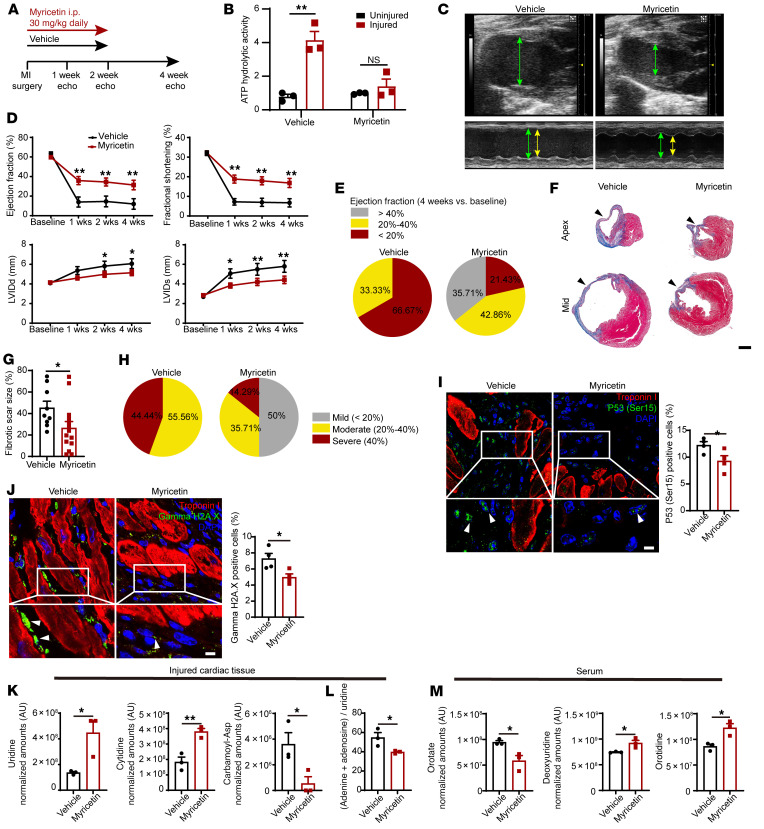
Animals treated with ENPP1 inhibitor myricetin demonstrate significant improvement in heart function after injury. (**A**) Strategy for using myricetin. (**B**) Extracellular ATP hydrolytic activity in injured and uninjured hearts of animals treated with myricetin (*n =* 3). (**C**) B and M mode echocardiogram demonstrating contractile function in diastole (green arrows) and systole (yellow arrows) in hearts of myricetin-treated animals. (**D**) EF, fractional shortening, and LV chamber size in systole and diastole in vehicle- or myricetin-treated animals. *n =* 12 (vehicle) and *n =* 15 (myricetin) at basal, 1 week, and 2 weeks; *n =* 9 (vehicle) and *n =* 14 (myricetin) at 3 weeks and 4 weeks. (**E**) Fractions of animals with mild, moderate, and severe reduction in EF at 4 weeks after injury. (**F**) Scar size as a fraction of LV surface area. Scale bar: 1 mm. (**G**) Quantitation of scar surface area. *n =* 9 (vehicle); *n =* 4 (myricetin). (**H**) Fractions of animals with mild, moderate, and severe fibrosis following myricetin administration. (**I** and **J**) Immunostaining demonstrating (**I**) p53 (Ser15 phosphorylation) expression (arrowhead) in nonmyocytes in the injured region of vehicle- versus myricetin–injected animals and under higher magnification (myocytes are stained by troponin) and quantification (*n =* 4). (**J**) pH2AX staining in nonmyocyte cells (arrowheads) in vehicle- or myricetin-injected animals at 7 days following injury, under higher magnification and quantitation (*n =* 4, counts normalized to number of nonmyocyte nuclei for **I** and **J**). Scale bars: 5 μm (high magnification). Low magnification, ×40. (**K**) Metabolomic analysis of the hearts of myricetin-injected animals. (**L**) Decreased adenine+adenosine/uridine ratios in myricetin-injected animals (*n =* 3). (**M**) Metabolomic analysis of serum demonstrating decreased orotate and increased deoxyuridine (day 3) and increased orotidine (day 7) in myricetin-injected versus vehicle-injected animals (*n =* 3). Data are represented as mean ± SEM. **P < 0.01; *P < 0.05, ordinary 2-way ANOVA with Šidák’s multiple comparisons test (**D**) or 2-tailed Student’s *t* test (**B**, **G**, and **I**–**M**).

Subsequently, we determined whether administration of myricetin can augment cardiac wound healing in vivo. We subjected C57BL/6 animals to ischemic cardiac injury and administered vehicle or 30 mg/kg myricetin intraperitoneally to the animals starting on the day of injury and continuing daily for 14 days after injury (Figure 11A). We harvested the injured hearts following 7 days of myricetin administration and observed significant suppression of ATP hydrolytic activity in the myricetin-injected animals (Figure 11B). Echocardiography demonstrated significantly better preservation of EF/fractional shortening (FS) and significantly decreased chamber size in the myricetin-treated group (Figure 11, C and D). More than 66% of the animals in the vehicle-injected group had severe heart failure, while only 23% of the animals receiving myricetin developed severe heart failure (Figure 11E and Supplemental Table 1A). The area of scarring was also significantly reduced in myricetin-treated animals (Figure 11, F and G). Only 15% of myricetin-treated animals developed severe fibrosis, in contrast with 56% in the vehicle-injected group (Figure 11H and Supplemental Table 1B). Myricetin-treated animals exhibited significantly lower heart weight/body weight ratios (Supplemental Figure 9G) and significantly increased capillary density (Supplemental Figure 9, H and I). However, the amount of surviving myocardium 24 hours after injury did not show any significant differences between vehicle- and myricetin-treated groups (Supplemental Figure 9, J and K), suggesting that the effects of ENPP1 inhibition are likely secondary to enhanced wound healing after cardiac injury rather than enhanced primary myocyte survival.

Arrest in pyrimidine biosynthesis in nonmyocyte cells initiated a DNA damage response with expression of γH2A.X and p53Ser15 phosphorylation. We performed immunostaining and observed attenuated expression of γH2A.X and p53Ser15 phosphorylation in nonmyocyte cells in hearts of myricetin-treated animals (Figure 11, I and J). We confirmed this observation in ENPP1CKO hearts as well. Following injury, a significantly lower number of CFs exhibited γH2A.X in ENPP1CKO hearts (Supplemental Figure 4, O and P).

Finally, as inhibition of ENPP1 should rescue pyrimidine biosynthesis, we performed metabolomic analysis of injured hearts of myricetin-treated animals. Myricetin administration resulted in significant increases in levels of the pyrimidines uridine and cytidine in the injured regions of the heart and exhibited decreases in carbamoyl phosphate levels compared with those in vehicle-treated injured hearts (Figure 11K). As uridine rescues the toxic effects of the combination of adenine and a purine nucleoside, we measured the adenine+adenosine/uridine ratio in cardiac tissue as a metric of cytotoxicity and observed that hearts of myricetin-treated animals exhibited a decreased cytotoxicity ratio (Figure 11L). We also performed metabolomic analysis of the serum and observed orotate to decrease in the serum of myricetin-treated animals, while deoxyuridine and orotidine levels increased, consistent with rescue of pyrimidine biosynthesis (Figure 11M). These in vivo data remarkably mirror our in vitro experiments demonstrating an arrest in pyrimidine biosynthesis at the OMP synthesis step and suggesting the potential use of serum orotidine as a biomarker to monitor wound healing in the heart.

## Discussion

Our observations demonstrate what we believe is a hitherto unappreciated role of cardiomyocytes in regulating the cardiac repair response by modulating pyrimidine biosynthesis and fates of nonmuscle cells. Myocardial necrosis leads to release of extracellular ATP that serves as a DAMP signal. AMP formed by ENPP1-mediated hydrolysis is directly metabolized by cardiomyocytes to form adenine and other purine nucleosides that are released into the extracellular space. Our isotope-labeling experiments suggest that AMP is directly used by the myocyte to generate adenine, and pathways of adenine generation could be related to direct nucleotide phosphorolysis mediated by APRT. The combination of adenine and specific ribonucleosides, such as adenosine or inosine, exerts cytotoxic effects on proliferating nonmyocytes by disrupting their pyrimidine biosynthesis. The imbalance of purines or pyrimidines is a key event initiating the cell-cycle arrest/apoptotic cascade as supplementation of uridine to correct decreased pyrimidine levels rescues cell death. Myocytes are nonproliferative, and although an optimal purine/pyrimidine balance is required for DNA repair in the long term, the nonproliferative state protects the myocyte against cell death resulting from a sudden imbalance of purine or pyrimidine bases after cardiac injury.

Our data suggest an inherent defect in cardiac repair based on the ability of nonproliferative cardiomyocytes to mount a “metabolic attack” and a DNA damage response in proliferative nonmyocyte cells that play a critical role in wound healing. Apoptosis of nonmyocytes is known to occur after cardiac injury and has been confirmed even in human heart samples (31, 32). However, the physiological significance of nonmyocyte apoptosis is unclear. In this regard, our data suggest that there is an optimal balance of fibroblasts, endothelial cells, and macrophages required for cardiac repair. Decreased apoptosis of CFs and endothelial cells in the ENPP1CKO animals was associated with decreased inflammation and led to decreased myofibroblast formation, ECM gene expression, and lesser scarring. These data suggest that myofibroblast activation could be regulated by nonmyocyte cell death after cardiac injury. The teleological reasons that a cardiac muscle cell would mount such a repair response that ultimately is counterproductive for cardiac healing remain unclear, but such mechanisms of metabolic control could have evolved as a defense response against rapidly proliferating noncardiac cells as in an invading cancer or microbes.

We identified myricetin as a potent inhibitor of ENPP1. Pharmacological targeting of ENPP1 with myricetin in vivo results in rescue of pyrimidine biosynthesis, with increased serum orotidine levels suggesting a rescue of OMP synthesis and the potential of using serum orotidine as a biomarker for monitoring disruption in pyrimidine biosynthesis. Although myricetin possesses antioxidant and antiinflammatory properties (33), its ability to inhibit ENPP1 activity in the injured heart along with its rescue of pyrimidine biosynthesis strongly favor a mechanism of benefit based on potent ENPP1 inhibition. In summary, our observations demonstrate a myocyte-dependent model of cardiac repair in which the cardiac muscle cell, by altering the extracellular metabolome, regulates biological functions of nonmyocyte cells and plays a pivotal role in determining how the heart heals itself.

## Methods

Detailed methods are provided in the Supplemental Methods.

### Data set availability.

The bulk RNA-Seq and single-cell RNA-Seq data in this paper are available in the NCBI’s Gene Expression Omnibus database (GEO GSE185060).

### Statistics.

All data are presented as mean ± SEM, and the value of *n* represents biological replicates. Statistical analysis was performed using GraphPad (Prism) software, version 9, using Student’s *t* test (2 tailed), ordinary 1-way ANOVA with Tukey’s multiple comparison test or ordinary 2-way ANOVA with Šidák’s multiple comparisons test as appropriate. The χ^2^ test was used for statistical analysis of contingency tables. *P* < 0.05 was considered statistically significant.

### Study approval.

All animal studies were approved by the Animal Research Committee, UCLA. All animals were maintained at the UCLA vivarium according to the policies instituted by the American Association for Accreditation of Laboratory Animal Care.

## Author contributions

SL performed the majority of experiments and data analysis. TY performed echocardiography imaging and analysis in a blinded manner. PW performed all surgical procedures. RW, YW, MN, and YZ performed bench experiments related to cellular and molecular assays of ENPP1. FM and MP analyzed single-cell and bulk RNA-Seq data. RD and MJ helped in high-throughput ENPP1 assay development and drug discovery. IG and KF helped in HPLC and analysis. JTH, TGG, UB, and M Sharpley helped in LC-MS runs and analysis. AJL and M Seldin performed HMDP analysis. CGR, ERA, and TML performed nucleotide measurements on samples with LC-MS. CGR also helped design metabolic experiments. SJB and HM assisted in cell-cycle analysis. DTB and VEM provided critical reagents, and AR maintained colonies and assisted with experiments. DH and MT performed QPM experiments. YJL and PYC performed traction forced microscopy experiments. AD conceptualized the project, designed experiments, supervised data collection and analysis, and wrote the manuscript.

## Supplementary Material

Supplemental data

Supplemental video 1

## Figures and Tables

**Table 1:**
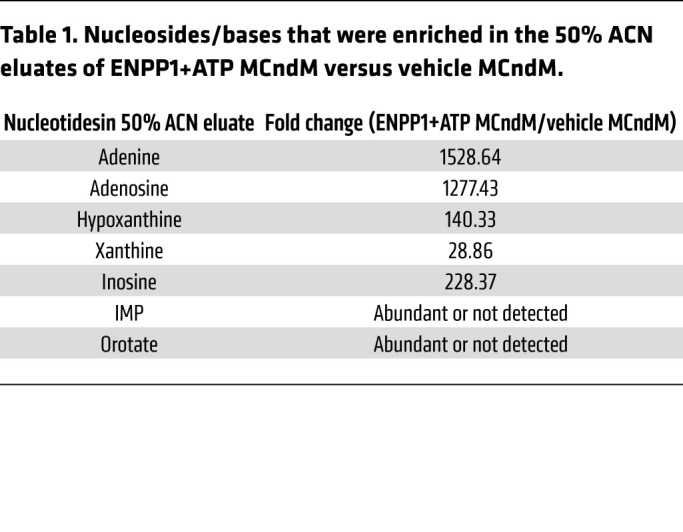
Nucleosides/bases that were enriched in the 50% ACN eluates of ENPP1+ATP MCndM versus vehicle MCndM.

## Version 1 11/23/2021

In-Press Preview

## Version 2 01/18/2022

Electronic publication
