# Supplementary material for: Cardiomyocytes disrupt pyrimidine biosynthesis in nonmyocytes to regulate heart repair
Source: J Clin Invest. 2022 Jan 18;132(2):e149711. doi: 10.1172/JCI149711 (PMC8759793; doi:10.1172/JCI149711)
Supplement: Supplemental data [file jci-132-149711-s021.pdf]

Supplemental Materials for

The cardiomyocyte disrupts pyrimidine biosynthesis in non-myocytes to regulate heart repair

Shen Li[1,2,3,4,5,6], Tomohiro Yokota[1,2,3,4,5,6], Ping Wang[1,2,3,4,5,6], Johanna ten Hoeve[7], Feiyang Ma[3,4,5], Thuc M. Le[7,13,17], Evan R. Abt[7,13,17], Yonggang Zhou[1,2,3,4,5,6], Rimao Wu[1,2,3,4,5,6], Maxine Nathavongdouangsy[1,2,3,4,5,6], Abraham Rodriguez[1,2,3,4,5,6], Yijie Wang[1,2,3,4,5,6], Yen-Ju Lin[6,14,20], Hayato Muranaka[16,17], Mark Sharpley[3,4,5], Demetrios T. Braddock[8], Vicky E MacRae[9], Utpal Banerjee[3,4,5,10], Pei-Yu Chiou[6,14,20], Marcus Seldin[11], Dian Huang[4,6,12,13], Michael Teitell[4,6,12,13], Ilya Gertsman[19], Michael Jung[15], Steven J Bensinger[16,17], Robert Damoiseaux[6,13,14,17], Kym Faull[18], Matteo Pellegrini[3,4,5], Aldons J Lusis[1,2,16], Thomas Graeber[7,13,17], Caius G Radu[7,13,17] & Arjun Deb*[1,2,3,4,5,6].

Correspondence to: adeb@mednet.ucla.edu

This PDF file includes:

Supplemental Methods and References
Figs. S1 to S9 with accompanying figure legends
Table S1 with accompanying table legends
Captions for Movies S1

Other Supplemental Materials for this manuscript include the following:
Movies S1

Supplemental Methods

Generation of animals with genetically labeled cardiac fibroblasts and fibroblast specific deletion of ENPP1.

Col1a2:CreERT or the TCF21MerCreMer mice were crossed with the lineage reporter Rosa26R:^{tdtomato} mice to generate Col1a2CreERT:R26R^{tdTomato} and TCF21MerCreMer:R26R^{tdTomato} mice. Tamoxifen (1mg i.p. daily, Sigma, T5648) was injected for 10 days prior to ischemic cardiac injury to determine whether tdTomato labeled cardiac fibroblasts express ENPP1. For generation of ENPP1CKO mice, Col1a2CreERT mice were crossed with the ENPP1 floxed mice (Bl/6 background) [1] to generate Col1a2CreERT:Enpp1$^{fl/fl}$ progeny mice. Tamoxifen (1mg i.p. daily,) was administered to these animals starting 5 days prior to ischemic cardiac injury and continued for 7 days after injury to conditionally delete ENPP1 in Col1a2 expressing cells to generate ENPP1CKO mice.

Murine models of acute ischemic cardiac injury

Myocardial infarction was performed by ligating the left anterior descending (LAD) coronary artery following open thoracotomy as described [2] . For ENPP1CKO mice, adult mice (both male and female), 10-14 weeks old were used. Tamoxifen (1mg i.p. daily) was injected for 5 days prior to myocardial infaction and continued for 7 days following injury. Cre negative (Cre-) littermates were used as controls.

To determine effects of uridine, male C57Bl/6 mice, 12-16 weeks old were used. Uridine (Sigma, U3003) dissolved in saline (Hanna Pharmaceutical, 0409488810), was infused continuously (150mg/kg/day) via osmotic pump (Alzet, 1002) for 14 days. Control animals only received saline. The osmotic pump was implanted subcutaneously 24 hours prior to ischemic cardiac injury.

To determine effects of myricetin, male C57Bl/6 mice, 12-16 weeks old were used. Myricetin (TCI, M2131) (30mg/kg/day) dissolved in 4% DMSO (Sigma, D2620) +30% PEG400(Sigma, 06855)+66% saline, was administrated intraperitoneally for 14 days starting from the day of myocardial infarction. Vehicle animals only received 4%DMSO+30% PEG400+66% saline.

Preparation of ENPP1+ATP myocyte conditioned medium (MCndM) and control MCndM.

Neonatal rat ventricular myocytes (NRVMs) were isolated from post natal P1-P3 day old Sprague-Dawley rat pups of mixed gender as previously described[3]. Fresh isolated NRVMs (20,000 cells/cm^2) were seeded in 0.2% gelatin (Sigma, G2500) pre-coated 12-well cell culture plates (Corning, 3513) in high glucose DMEM (Gibco, 1195073) supplemented with 10%FBS (Gibco, 16140071) and 1% penicillin and streptomycin (Gibco, 15140122) and cultured for 24 hours (1mL medium/well). Cells were then washed with PBS and maintained for 24 hours in serum-free DMEM/ITS medium which was made from high glucose DMEM (Gibco, 1195073) supplemented with 0.1% Insulin-Transferrin-Selenium (ITS) (Corning, 354351) and 1% penicillin and streptomycin. After 24 hours, NRVMs were then incubated with fresh serum-free DMEM/ITS medium mixed with 1nM human recombinant ENPP1 (Prospec, ENZ-729) and 100 μM ATP (R1441, Thermo Scientific) for 24 hours. Conditioned medium (ENPP1+ATP MCndM) was then harvested, centrifuged at 500xg for 5min to remove cell debris, and then supenatant conditioned medium was collected for treating cardiac fibroblasts (CFs) or other cells.

For control MCndM, vehicle MCndM was collected by incubating NRVMs with identical serum-free DMEM/ITS medium for 24 hours but without adding ENPP1 or ATP. Control ENPP1 MCndM was collected by incubating NRVMs with serum-free

DMEM/ITS medium for 24 hours mixed with 1nM ENPP1 protein but without any added ATP. Control ATP MCndM was collected by incubating NRVMs with identical DMEM/ITS medium for 24 hours with ATP but without any ENPP1. PPi MCndM was made by incubating NRVMs with serum-free DMEM/ITS medium mixed with 100 μM pyrophosphate (Sigma, 71501); AMP MCndM was made by incubating NRVMs with serum-free DMEM/ITS medium mixed with 100 μM AMP (Acros, 102790050).

For testing adenosine receptor agonist effects on NRVMs, NECA MCndM was collected by inbubating NRVMs with serum-free DMEM/ITS medium mixed with 10 μM NECA (Sigma, E2387).

Treatment of CFs with ENPP1+ATP MCndM.

Immortalized CFs were seeded at low density (10,000 cells/cm^2) at approximately 20-30% confluency in 24 well plates (Corning, 3524) in high glucose DMEM (Gibco, 1195073) supplemented with 10%FBS (Gibco, 16140071) and 1% penicillin and streptomycin (Gibco, 15140122) and cultured for 24 hours (500 μL/well). Cells were then washed with PBS and maintained for another 24 hours in serum-free DMEM/ITS medium which was made with high glucose DMEM supplemented with 0.1% Insulin-Transferrin-Selenium (ITS) and 1% penicillin and streptomycin. After 24 hours, DMEM/ITS medium was replaced by ENPP1+ATP MCndM or control MCndM medium and then cells incubated for 48 hours prior to analysis of cell death by flow cytometry using Propidium Iodide/Annexin V assay.

Co-culture of cardiomyocytes (CMs) with cardiac fibroblast.

NRVMs (CM) (20,000 cells/cm^2) and immortalized ENPP1 overexpressing CFs (ENPP1 CFs) or immortalized control eGFP expressing cardiac fibroblasts (Control-CF) (10,000 cells/cm^2) were seeded in 0.2% gelatin (Sigma, G2500) pre-coated 12-well cell culture plates (Corning, 3513) in high glucose DMEM (Gibco, 1195073)

supplemented with 10%FBS (Gibco, 16140071) and 1% penicillin and streptomycin (Gibco, 15140122) for 24 hours. After 24 hours, cells were washed with PBS and maintained in serum-free DMEM/ITS medium which was made by high glucose DMEM supplemented with 0.1% Insulin-Transferrin-Selenium (ITS) (Corning, 354351) and 1% penicillin and streptomycin for 24 hours. Medium was then replaced by fresh serum-free DMEM/ITS medium mixed with 100μM ATP. After 48 hours of incubation, CM and CFs cell numbers were counted. The NRVM and CF co-culture incubated with fresh serum-free DMEM/ITS but without adding ATP was used as control .

In vitro treatment of CFs with metabolites or compounds.

Immortalized CFs were seeded at low density (10,000 cells/cm^2) at 20-30% confluency in 24 well plates (Corning, 3524) in high glucose DMEM +10%FBS + 1% penicillin and streptomycin for 24 hours. Cells were then washed with PBS and maintained in serum-free DMEM/ITS medium for another 24 hours. Subsequently, cells were cultured with various compounds or metabolites mixed in fresh serum-free DMEM/ITS medium for 48 hours. For the DHODH inhibitor experiment, 100 nM brequinar or 100nM brequinar + 25 μM uridine mixed in fresh serum-free DMEM/ITS medium was added to CFs. For the effect of individual metabolites on CFs, 100 μM AMP, 100 μM adenine (Sigma, A2786), 100 μM adenosine (Acros, 164040050), 100 μM hypoxanthine (Sigma, H9636), 100 μM xanthine (Sigma, X3627), 100 μM inosine (Sigma, I4125), 100 μM IMP (Sigma, 57510) or 100 μM orotate (Sigma, O2750) individually mixed in fresh serum-free DMEM/ITS medium was added to CFs; all metabolites were dissolved in serum-free DMEM/ITS medium at 1M stock and sonicated for 30 minutes to create a homogeneous solution.

For testing effects of 7 metabolites in combination, 100 μM of adenine,

adenosine, hypoxanthine, xanthine, inosine, IMP and orotate were mixed together in serum-free DMEM/ITS medium and then added to CFs. 25 µM uridine was used in 7 metabolites + uridine group to determine rescue of cell death.

For experiments where each compound was substracted one by one from the combination of 7 metabolites, we mixed 100 µM of 6 of 7 metabolites in fresh DMEM/ITS serum-free medium. For the addition of adenine with each of other 6 metabolites, 100 µM adenine was added to 100 µM each of hypoxanthine, xanthine, orotate, inosine, IMP, adenosine or AMP. Fresh serum-free DMEM/ITS medium without any added metabolites served as vehicle control in these experiments.

Echocardiographic studies

Echocardiogram was performed at baseline prior to injury and then at 1 week, 2 weeks, and 4 weeks post myocardial infarction. For echocardiography, animals were anesthetized with a mixture of 1.5% isoflurane and 95% O_2. Vevo-2100 imaging system and a 30-mHz transducer (VisualSonics, Vevo2100) were used to acquire short/long axis B-mode and M-mode images. All measurements and analysis were used by Vevo Lab software.

Isolation of primary adult cardiac fibroblasts

5-6 hearts were harvested from wild-type C57Bl/6 mice or P53 floxed mice. Valves and atriums were removed, and the ventricle rinsed in ice-cold HBSS. The hearts were digested by 0.1µg/ml liberase TH (Sigma, 5401151001) in Tyrodes buffer (136mM NaCl, 5.4mM KCl, 0.33mM NaH2PO4, 1mM MgCl2, 10mM HEPES, 0.18% Glucose), and resuspended cells were seeded onto a 100mm^2 dish. After 2 hours, medium was changed to F12K (Gibco, 21127022) medium contained 20% FBS, human basic FGF (10ng/ml, Millipore, GF003) and 1% penicillin and streptomycin. Isolated cardiac fibroblasts between 2nd and 3rd passage were typically used for

experiments.

Generation of immortalized cardiac fibroblasts

Primary cardiac fibroblasts (CFs) were infected with Lentivirus-Large T antigen in presence of polybrene (8µg/ml) for 16 h, then replaced by regular medium for 48 hours. Following that, medium containing puromycin (2µg/ml) was added to aid selection of immortalized cells. Immortalized cardiac fibroblasts were then cultured with high glucose DMEM containing 10% FBS[4].

For generation of ENPP1 overexpressing CFs (ENPP1 CFs), wild-type C57Bl/6 immortalized CFs were infected with letivirus-mouse ENPP1 or lentivirus-eGFP to generate mouse ENPP1 CFs or control CFs respectively.

For generation of mutant ENPP1 CFs, wild-type C57Bl/6 immortalized CFs were infected with a lentivirus encoding mutant ENPP1. For construction of mutant mouse ENPP1, mutant ENPP1(T737A) was made with QuikChange II Site-Directed Mutagenesis Kit (Agilent Technologies, Catalog #200523). Two mutagenesis primers used TCGACACAGGACTTTATCCGGAATCC (F), TGCTGTAATGATTGGGAAACG(R) were used. Briefly Mutant Strand Synthesis Reaction for mutant ENPP1(T737A) was done according to manufacturer instruictions for QuickChange II site -Directed Mutagenesis kit. A PCR program of: 1. 95°C for 30 second, 2. 95°C for 30 second, 3. 55°C for one minute, 4. 68°C for 11minutes, 5. followed by repeat of step 2-3 for 12 cycles was used. The amplification production was digested with DpnI, and then, the digestion product was transformed into XL-1 competent cells. The mutagenesis clone was confirmed by sequencing.

For generation of P53CKO CFs, cardiac fibroblasts isolated from p53 floxed mice were immortalized and then infected with Lentivirus-eGFP-Cre recombinase for

16 hours, and cultured for 2-3 passages to generate sufficient number of P53CKO CFs. Cells were purified by flow cytometry sorting of eGFP positive cells.

For generation of adenine deaminase over-expressing CFs, wild-type C57Bl/6 immortalized CFs were infected with letivirus-Adenine deaminase (pLV[Exp]-EGFP:T2A:Puro-EF1A>sce_AAH1[NM_001182979.1]) for 16 hours, then replaced by regular medium and cultured for 2-3 passages to generate adenine deaminase CFs. Cells were purified by flow cytometry sorting of eGFP positive cells.

Compounds used to treatment CFs when adding ENPP1+ATP MCndM

Various compounds were added to CFs at the time of adding ENPP1+ATP MCndM. Uridine 25 μM, deoxycytidine 10 μM (Sigma, D3897), (R)-DI-87 2 μM, Oritidine monophosphate 50 μM (Biosynth, NO71879), AB680 100 nM (MCE, HY-125286), AMP-CP 100 μM (Sigma, M3763), DPCPX 500 nM (Sigma, C101), 8-3-caffeine 10 μM (Sigma, C197), Alloxazine 10 μM (Sigma, A28651), Cpd3 50 μM (EMD Millipore, 533642), ABT-702 1 μM (Tocris, 2372), Myricetin 10 μM were the compunds added to cardiac fibroblasts.

RNA extraction and Q-PCR

Total RNA from cells was extracted using RNA mini Kit (BioRad, 7326830). For heart tissue RNA extraction, the uninjured and injured regions of the heart were harvested and homogenized in lysis buffer and then extracted using RNA mini kit. cDNA was synthesized by using iScript cDNA Synthesis Kit (BioRad, 1708890) and qPCR performed.

RNA sequencing

Total RNA was used to generate RNA-seq libraries followed by sequencing using Illumina Highseq 4000 platform (single end, 50bp). The reads were mapped with STAR 2.5.3a[5] to the mouse genome (mm10). The counts for each gene were

obtained by using –quantMode GeneCounts commands in STAR, and the other parameters during alignment were set to default. Differential expression analyses were carried out using DESeq2[6]. Sequencing depth normalized counts were obtained from the differential expression analysis and were used for principal component analysis. Enrichr[7] was used to perform enrichment analysis, pathways with adjusted p value < 0.05 were considered significantly enriched.

Single cell RNA sequencing

For sc-RNA-seq, 7 days post MI ENPP1CKO and control littermate mice hearts were harvested and digested by 0.1μg/ml liberase TH (Sigma, 5401151001). After digestion, cells were incubated with 10μM Calcein AM (Abcam, ab141420) and flow sorted to identify live cells followed by library preparation. Equal number of cells from 3 mouse hearts were mixed for each group. For the generation of single-cell gel beads in emulsion, cells were loaded on a Chromium single cell instrument (10x Genomics) with an estimated targeted cell recovery of ~5,000 cells as per manufacturer's protocol. In brief, single-cell suspension of cells in 0.4% BSA-PBS were added to each channel on the 10x chip. Cells were partitioned with Gel Beads into emulsion in the Chromium instrument where cell lysis and barcoded reverse transcription of RNA occurred following amplification. Single cell RNA-Seq libraries were prepared by using the Chromium single cell 3′ library and gel bead kit v2 (10x Genomics). Sequencing was performed on Illumina Highseq 4000.

Single cell RNA sequencing data analysis

Raw reads were aligned to the mouse genome (mm10) and the digital expression matrix was generated using cellranger count. Individual samples were merged to generate the digital expression matrix using cellranger aggr. To identify different cell types and find signature genes for each cell type, the R package Seurat[8] was used

to analyze the digital expression matrix. Cells with less than 100 genes and greater than 10% mitochondrial expression were removed from further analysis. Seurat function NormalizeData was used to normalize the raw counts. Variable genes were identified using the FindVariableGenes function. The Seurat ScaleData function was used to scale and center expression values in the dataset for dimensional reduction. Principal component analysis (PCA), t-distributed stochastic neighbor embedding (t-SNE), and uniform manifold approximation and projection (UMAP) were used to reduce the dimensions of the data, and the first two dimensions were used in plots. The FindClusters function was later used to cluster the cells. The FindAllMarkers function was used to determine the marker genes for each cluster, which were then used to define cell types. The FindMarkers function was used to determine the differentially expressed genes between two group of cells. Genes with adjusted p value < 0.05 was considered significantly differentially expressed. Enrichr[7] was used to perform enrichment analysis, pathways with adjusted p value < 0.05 were considered significantly enriched.

Immunoblotting

Immunoblotting was performed using lysates from cell pellets or homogenized tissue samples in RIPA buffer (Thermo Scientific, 89900) containing protease and phosphatase inhibitor cocktails (Thermo Scientific, 78442). Protein was quantified by BCA assay (Thermo Scientific, 78442) and equal amount of proteins were loaded on 4% to 12% Tris-Glycine precast gels (Thermo Scientific, XP04125BOX) for Western blot analysis. Gels were then subjected to semi-dry transfer onto PVDF membranes, which were then probed overnight with primary antibodies. Membranes were then incubated with appropriate horseradish peroxidase–conjugated secondary antibody, followed by incubation with ECL western blotting sbustrate (Thermo

Scientific, 32209), and chemoluminescent signal detected by western blot imaging system (Cytiva, IQ600).

Heart tissue ATP hydrolytic activity assay

Uninjured and injured regions of hearts from wild type C57BL6 mice, ENPP1(asj/asj) , ENPP1 (wt/wt) littlemates, or myricetin treated mice and control vehicle mice were harvested at day 7 post MI and homogenized by assay buffer (50mM Tris + 2mM $MgCl_2$ +0.005% Tween 20 + 0.1% BSA) with protease and phosphatase inhibitor cocktails. Lysate was kept on ice for 30 minutes, and then centrifuged at 3000xg, 4°C to remove debris. Lysate was then diluted and passed through 3 kD Spin Column (Millipore, UFC900324) to retain the protein fraction. Protein rich lysate was normalized to protein concentration, subsequently incubated with ATP (2μM) for 2 hours at room temperature and residual ATP level was measured by Cell titer-Glo luminescent Assay (Promega, G7572).

Histological studies

Animals were euthanized before necropsy, hearts were harvested and fixed in 4% paraformaldehyde in PBS at 4˚C overnight, and subjected to dehydration in 10% and 30% sucrose solution. Subsequently hearts were snap frozen in Tissue-Tek O.C.T compound (SAKURA Finetek, 4583) and sectioned at 10μm-thickness.

For immunofluorescence staining, tissue sections were post fixed in 4% paraformaldehyde for 10 minutes, and then permeabilized in 0.1% triton X100 for 15 minutes. For TUNEL staining of tissue section, sections were first subjected to antigen retrieval. Slides were placed in 0.1M citrate buffer, pH 6.0 and treated with microwave irradiation for 1 min. Sections were blocked in 10% species-specific normal serum in 1% BSA/PBS for 1 h, and incubated with primary antibodies diluted in 1% BSA/PBS at 4˚C overnight. Secondary antibodies were diluted in PBS and

incubated with the sections for 1 h. Samples were counterstained with DAPI (Invitrogen, D3571) and mounted with antifade media (Invitrogen, S36936). Images were taken using Nikon Eclipse Ti2 confocal microscopy (Nikon,USA) and analyzed in NIS Element AR software (Nikon) or image J.

For determing myocyte size in ENPP1CKO mice post injury, the cross sectional area of 100 cardiac myocytes in the injured region was measured in each mouse. CD31 was used for capillary density measurement, CD68 was used for macrophage cell ratio measurement, pH2AX and p53 (Ser15) were used for non myocytes DNA damage evaluation, alpha smooth muscle actin was used to identify myofibroblasts, Ki67 was used to determine proliferation, and positive cells in 6 images were measured in each animal. TUNEL *in Situ* Cell detection kit (Sigma, 11684795910) was used for apoptotic cell measurement, positive cells in 5 images were measured in each sample.

For immunohistochemical staining, sections were stained with hematoxylin and eosin (Fisher chemical, SE23-500D) or Masson Trichrome Staining (Thermo Scientific, 87019). Sections were scaned using Aperio AT2 (Leica, Germany) and fibrotic area analyzed in heart sections from apex to mid-ventricle. Scar tissue area was calculated as the fraction of left ventricular surface area occupied by the scar tissue.

For TTC staining to determine viable and dead myocardium in ENPP1CKO or myricetin treated mice post injury, hearts were harvested 24 hours after ischemic cardiac injury and cut into 1mm slices. Slices were incubated in 1% 2,3,5-Triphenyltetrazolium chloride (Sigma, T8877) solution for 30 minutes at 37˚C in the dark.

Immunocytochemistry staining and analysis for co-culture of cardiomyocytes

and cardiac fibroblasts.

After 48 hours of co-culture of cardiac myocytes and cardiac fibroblasts (i.e. after adding ATP), cells were rinsed with PBS and fixed in 4% paraformaldehyde for 10 minutes. All cells were stained with fluorescent labeled wheat germ agglutinin (Thermo Scientific, W11261) and cardiomyocytes were identified by Troponin I staining. Images were taken using Nikon Eclipse Ti2 confocal microscopy (Nikon,USA) and analyzed by image J. Four images were captured in each well, and cardiac fibroblasts and cardiomyocytes counted.

TUNEL and cleaved-caspase 3 staining were used for quantification of CF apoptosis. After 48 hours of MCndM incubation, CFs were rinsed by PBS and fixed in 4% paraformaldehyde for 10 minutes. Cells were then subjected to TUNEL staining or cleaved-caspase 3 immunostaining and cell nuclei were counterstained with DAPI. Images were taken using Nikon Eclipse Ti2 confocal microscopy (Nikon,USA) and analyzed by image J. Four images were captured in each well for counting TUNEL and cleaved-caspase 3 positive cells.

Propidium Iodide / Annexin V assay.

To detect cell death, Propidium Iodide (PI)/Annexin V staining were used for flow cytometry analyses. After incubation with myocyte conditioned medium, cells were suspended in 100μL annexin V binding buffer (BioLegend, 422201), to which 5μL of Annexin V(Biolegend, 640941) and 10μL of PI (BD, 51-66211E) were added, then incubated for 15 minutes at room temperature in the dark. Subsequently cells were subjected to flow cytometry (BD, LSRII) and data was analyzed by FlowJo V10 software to determine the fraction of PI+ or Annexin+PI negative cells.

Treatment of other types of cell with ENPP1+ATP MCndM.

Primary CFs were seeded at low density (10,000 cells/cm^2) with about 20-30%

confluency in 24 well plates in Ham's F12K medium (Gibco, 2127022) supplemented with 20%FBS, 1% penicillin and streptomycin and human basic FGF (10ng/ml, Millipore, GF003) and cultured for 24 hours (500 µL/well). Cells were then washed with PBS and maintained in serum-free DMEM/ITS medium for another 24 hours. After 24 hours, DMEM/ITS medium was replaced by ENPP1+ATP MCndM or control MCndM medium and then cells incubated for 48 hours prior to analysis.

Bone marrow derived macrophages were seeded at 20-30% confluency in (DMEM + 10% FBS + 5% M-CSF conditioned medium + 1% glutamine + 0.5% sodium pyruvate) for 24 hours (500 µL/well). Cells were then washed with PBS and maintained in serum-free DMEM/ITS medium for another 24 hours. After 24 hours, DMEM/ITS serum-free medium was replaced by ENPP1+ATP MCndM or control MCndM medium and then cells incubated for 48 hours prior to analysis.

Human primary aortic vascular smooth muscle cells (HVSMC, ATCC, PCS-100-012) were seeded at 20-30% confluency in vascular cell basal medium (ATCC, PCS-100-030) supplemented with vascular smooth muscle cell growth factor kit (ATCC, PCS-100-042) for 24 hours (500 µL/well). Cells were then washed with PBS and maintained in DMEM/ITS serum-free medium for another 24 hours. After 24 hours, DMEM/ITS serum-free medium was then aspirated and replaced by ENPP1+ATP MCndM or control MCndM medium and then cells incubated for 48 hours prior to analysis.

Human umbilical vein endothelial cells (HUVEC, Lonza, C2519A) were seeded at 20-30% confluency in Endothelial Cell Growth Medium-2 (Lonza, CC-3162) for 24 hours (500 µL/well). Cells were then washed with PBS and replaced by ENPP1+ATP MCndM or control MCndM medium and then cells incubated for 48 hours prior to analysis.

Induction of cell cycle arrest of cardiac fibroblasts with irradiation or mitomycin C

γ-irradiation or cell cycle inhibitor mitomycin C were used to induce fibroblast cell cycle arrest. For γ-irradiation, primary cardiac fibroblast and mouse embryonic fibroblasts (mEFs) (ATCC, SCRC-1040) were seeded in 0.2% gelatin pre-coated dishes, and then γ-irradiated with 6,000 rads. Irradiated cells were then ready for use. For mitomycin C, primary cardiac fibroblasts and MEFs were treated with 10 μg/mL mitomycin C (Tocris, 32-581-0) for 2.5 hours, then cells re-seeded in 0.2% gelatin pre-coated dishes for further use. Following irradiation or mitomycin C treatment, cells were seeded at low density (10,000 cells/cm^2) at approximately 20-30% confluency in 0.2% gelatin pre-coated 24 well plates in high glucose DMEM supplemented with 10%FBS and 1% penicillin and streptomycin and cultured for 24 hours (500 μL/well). Cells were then washed with PBS and maintained in serum-free DMEM/ITS medium for another 24 hours. After 24 hours, DMEM/ITS medium was replaced by ENPP1+ATP MCndM or control MCndM medium and then cells incubated for 48 hours prior to analysis.

Cell Cycle Analysis

Following treatment with ENPP1+ATP myocyte conditioned medium, cells were trypsinized and washed with FACS buffer (PBS with 2% FBS and 1 mM EDTA). Next, cells were incubated with 50 μg/ml propidium iodide (CALBIOCHEM, #537059) and 0.1% triton X-100 (Fisher Scientific, BP151) in 0.1% sodium citrate (Sigma, S1804) for 15 min on ice. PI fluorescence was analyzed using a flow cytometer (Attune NxT). Percentages of cells in each phase were calculated using FlowJo V10.

Heat inactivation or fractionation of myocyte conditioned medium into protein poor and rich fractions.

To determine whether the pro-apoptotic molecules were proteins or metabolites, we heat treated conditioned medium to denature heat sensitive proteins. The conditioned medium was subjected to 95 °C for 15 minutes and then cooled to room temperature prior to adding it to CFs. To separate the myocyte conditioned medium into protein rich and poor fractions, we passed the conditioned medium through a protein fractionation column with a 3 kD filter (Millipore, UFC900324), and then treated CFs with the protein rich (>3kD) or protein poor fractions (<3kD) of the conditioned medium.

Metabolomics Analysis

<u>Sample preparation</u>

To determine the identity of the metabolites, myocyte conditioned medium was collected for LC/MS. Briefly, 20 µL MCndM was mixed in 500 µL cold 80% methanol (pre-cooled with dry ice over 30 minutes) and incubated for 20 minutes at -80 °C, then spun for 10 minutes at 15,000x g at 4°C. After that, supernatant was transferred to a glass vial and vacuum dried for LC-MS.

To determine metabolites in pyrimidine biosynthesis in CF, we treated CFs with ENPP1+ATP MCndM or control MCndM for 24 hours and measured the metabolites in the pyrimidine biosynthesis pathway. Briefly, after 24 hours incubation with MCndM, CFs (10,000 cells/cm^2 seeding density) in each well of 6 well plates was quickly rinsed with ice-cold 150mM NH_4AcO, pH7.3 and then 1 mL cold 80% methanol was added and the plate incubated for 20 minutes at -80 °C. The cells were scraped from the plate and the entire contents of the plate transferred to an

Eppendorf tube, vortexed and centrifuged at 15,000x g for 15 minutes at 4 °C. Subsequently, supernatant was transferred to a glass vial and vacuum dried for LC/MS.

For metabolomic analysis of the heart of wild type and myricetin treated animals after cardiac injury, 10.5mg injured tissue was homogenized in 1 ml 80% methanol and incubated for 30 minutes on dry ice. After that, homogenate was vortexed and centrifuged at 15,000x g for 15 minutes at 4 °C. Subsequently, supernatant was transferred to a glass vial and vacuum dried for LC/MS.

For metabolomic analysis of the serum of wild type and myricetin treated animals after cardiac injury, 50 µL serum was mixed with 50 µL H_2O and 400 µL 100% methanol and placed at -80°C for 20 minutes. After 10 seconds of vortexing, the mixture was centrifuged at 15,000x g for 10 minutes at 4 °C. Supernatant was then transferred to a new Eppendorf tube which included 300 µL H_20 and 400 µLchloroform and thoroughly homogenized for 1 minute. The mixture was then centrifuged at 15,000x g for 10 minutes at 4 °C. 600 µl of the top (aqueous) layer was transferred to a glass vial and vacuum dried for LC/MS.

To investigate whether AMP was directly utilized by CM for adenine synthesis. $^{15}N_5$ ATP (Sigma, 707783) and recombinant ENPP1 protein were added to CM. After 24 hours, the cardiomyocytes and 20 µL of the conditioned medium were harvested for LC/MS analysis. Cardiac fibroblasts treated for 24 hours with the conditioned medium collected was also harvested for LC/MS analysis.

<u>LC-MS analysis</u>

Vacuum dried samples prepared as described above was resuspended in 50% ACN:water and 1/10th was loaded onto a Luna 3um NH2 100A (150 × 2.0 mm)

column (Phenomenex). The chromatographic separation was performed on a Vanquish Flex (Thermo Scientific) with mobile phases A (5 mM NH4AcO pH 9.9) and B (ACN) and a flow rate of 200 µl/min. A linear gradient from 15% A to 95% A over 18 min was followed by 9 min isocratic flow at 95% A and reequilibration to 15% A. Metabolites were detection with a Thermo Scientific Q Exactive mass spectrometer run with polarity switching (+3.5 kV/− 3.5 kV) in full scan mode with an m/z range of 70-975 and 70.000 resolution. TraceFinder 4.1 (Thermo Scientific) was used to quantify the targeted metabolites by area under the curve using expected retention time and accurate mass measurements (< 5 ppm). Relative amounts of metabolites were calculated by summing up the values for all isotopologues of a given metabolite [9]. Metabolite isotopologue distributions were corrected for natural C13 abundance[10, 11]. Data analysis was performed using in-house R scripts.

CN or C18 HPLC column elution assay

To filter potential candidates differentially present in the ENPP1+ATP MCndM, we performed hydrophilic or hydrophobic column elution assays to determine physico-chemical properties of the candidates, we passed the ENPP1+ATP MCndM through a Strata CN hydrophilic column (Phenomenex, 8B-S007-HCH), the hydrophobic elute as well as the hydrophilic retentate were vacuum dried, reconstituted and added to CFs. We also passed the ENPP1+ATP MCndM through a Strata C18-E hydrophobic column (Phenomenex, 8B-S001-HCH), the hydrophilic elute as well as the hydrophobic retentate were vacuum dried, reconstituted and added to CFs.

For the ENPP1+ATP MCndM gradient eluted from a C18-E column by various concentrations of acetonitrile, we passed the ENPP1+ATP MCndM through a Strata C18-E hydrophobic column and eluted the column at 5, 25, 50, 75 and 100% of ACN. The fractional elutes were vacuum dried, reconstituted and added to CFs.

HPLC with fractional separation of myocyte conditioned medium into 80 eluates using linear graded increase of acetonitrile

10 mL Vehicle or ENPP1+ATP conditioned medium [followed by three washes of 3 mL each of eluant A (water/formic acid, 100/0.1, v/v)] were loaded (1.5 mL/min) onto two reversed phase HPLC columns connected in series (Keystone Scientific C18 Aquasil, 10 x 250 mm, 5 µm particle size, 100 Å pore diameter, followed by an Altex Ultrasphere ODS column, 5 µm particle size, 10 x 250 mm) and equilibrated in 95% eluant A and 5% eluant B (acetonitrile/formic acid, 100/0.1, v/v). There was no significant change in the UV spectrum (200-600 nm) of the eluate recorded with a diode array detector during the sample loading process, indicating that the columns had not been saturated with sample during the loading process. The separation process was initiated with an injection of eluant A (250 uL) and the columns were eluted (1.5 mL/min) with an increasing concentration of eluant B (min/% B: 0/5, 5/5, 65/100, 70/5, 80/5). Recordings of absorption of the eluate were made at 215 and 280 nm following which fractions (2 min) were collected in glass test tubes. For the testing aliquots (3 µL each) of the fractions were pooled (1-10, 11-20, 21-30, 31-40, 41-50, 51-60, 61-70, 71-80), vacuum dried and reconstituted in serum-free DMEM+ITS medium. The effect of the eluates on CF viability was measured by cell counting kit-8 assay. Blank injections of water (250 uL) loaded between the Vehicle and ENPP1+ATP conditioned medium samples showed negligable carry-over of UV absorbing material in the eluate, suggesting minimal carryover between chromatographic runs.

Plasmid construction and Lentivirus preparation

pLenti-Largen T antigen (Cat#18922), Lenti-pHIV-eGFP (Cat#21373), PSPAX2 (Cat#12260), PMD.G2 (Cat#12259), pLV-eGFP-Cre (Cat#86805) were purchased

from Addgene. pLV[Exp]-EGFP:T2A:Puro-EF1A>sce_AAH1[NM_001182979.1] (VB200228-1791ejw, Vectorbuilder) was purchased from vectorbuilder. Murine ENPP1 was cloned into Lenti-pHIV-EGFP with Gibson Assembly Protocol (NEB, E5510) by using restriction enzymes BbaI and BamHI. The clone was confirmed by sequencing. For lentivirus preparation, a total of 13ug pasmids (transgene, PSPAX2, PMD.G2) was co-transfected into 75cm^2 flask with 293T cells in 10 ml DMEM Medium. 7ml fresh DMEM medium was added after 10 hours of co-transfection of plasmids. The medium was collected and centrifuged at 4°C in 500x g for 10 min after transfected 72 h. The medium with virus was aliquoted and stored at -80˚C.

Isolation of Adult Mouse Cardiomyocytes

Cardiac myocytes were isolated from 10 to 14 week-old C57BL6 mice, as described[12]. In brief, mice were injected with heparin (200 USP units i.p.) and the heart was quickly excised and arrested in ice-cold Ca free Tyrodes solution. Hearts were digested by liberase TH perfused in the Langendorff equipment for 18 minutes with gentle shaking to liberate the cardiomyocytes. Cells were centrifuged at 50x g to harvest cardiac myocytes.

hPSC derived cardiomyocyte differentiation.

Cardiomyocytes were derived from hESC line H9 as described[13]. Briefly, hPSCs were maintained in mTeSR1 medium (stemcell technology, 85850). RPMI 1640 (Gibco, 22400-089) with B27 Supplement minus insulin (Thermo Scientific, A1895601) was used as differentiation medium. On day 0-1, 6 μM CHIR-99021 (Selleckchem, CT99021) was added into differentiation medium. 5 μM IWR1 (Sigma, I0161) was added to refreshed differentiation medium between day 3-5. After 7 days, medium was replaced with RPMI 1640 plus B27 CM maintain medium. From day 10-11, RPMI 1640 without D-glucose (Gibco, 11879-020) supplemented with B27 was

transiently used for metabolic purification of CMs.

Quantitative phase microscopy (QPM) measurements of projected single cell area and biomass changes in cardiac fibroblasts.

Quantitative phase imaging (QPI). Cells seeded on a 4-well ibidi plastic bottom slide (ibidi) were imaged once every 30 m for 24 h using a 20x, 0.4 numerical aperture objective lens on an Axio-vision Observer Z1 (Zeiss) microscope. The microscope was equipped with a SID-4 Bio (Phasics) camera to acquire QPM data by quadriwave lateral shearing interferometry[14]. Cell illumination was 660 nm with a center wavelength collimated LED (Thorlabs). For each experiment, cell imaging exceeded 60 unique positions per well, with wells containing either control or treatment media, and enough spacing in between cells for automated image processing and biomass segmentation.

Image processing. All image processing used custom MATLAB (MathWorks) scripts. Cells were identified and segmented using a local adaptive threshold based on Otsu's method[15] and tracked using particle tracking code based on Grier et al.[15, 16].Particle tracking data from segmented cell images provided the dry cell biomass based on the projected area of each cell at specific imaging times. This conversion relied upon an experimentally determined cell-average specific refractive index, with measured quantitative phase shifts translated into the dry biomass of the imaged cells[17, 18].

Statistical analyses. Cell dry biomass and projected cell area was binned into 1 h intervals and segregated based on either control or media treated conditions. Statistical assessments utilized an unpaired two-way analysis of variance and covariance for media conditions and time. Statistical analyses were performed using built-in MATLAB functions anovan and multcompare. The center circles within each

dry cell biomass and cell area boxplot indicates the population median value, with the triangles indicating comparison intervals, and the box edges representing 25 and 75 percentile population values, respectively.

Hybrid mouse diversity panel

For Enpp1 expression and trait analysis in the Hybrid Mouse Diversity Panel (HMDP), RPKM expression values of Enpp1 and echocardiographic traits were analyzed as described [19, 20]. Specifically, the abundance of Enpp1 in the left ventricle variation under both control and isoproteronol conditions was visualized for variation using R open source software and the package ggplot2. Midweight bicorrelation coefficients and corresponding pvalues (students pvalue, based on regression) between expression values and echocardiographic traits were generated using the package WGCNA, adjusted for false discovery rate using the package qvalue and plotted in ggplot2.

ENPP1 inhibitor high throughput screening

To identify small molecule inhibitors of ENPP1, we established a cell free luciferase based luminescent assay and screened a large small molecule library over 200,000 compounds available at our institution. Small molecules were dispensed by automated workstation (Beckman, Biomek FX) in 384 well plates (Corning, 4513). Every 384 well plate included 32 wells each for negative and for positive internal controls. Negative controls included human recombinant ENPP1 protein and ATP while positive controls included ATP without ENPP1. Human ENPP1 recombinant protein was added to all experimental wells with an automated washer dispenser (BioTek, EL406) and incubated at room temperature. After 30 minutes, ATP was added to all the wells in the plate by washer dispenser and incubated at room temperature for 120 minutes. Cell titer-Glo was then added to whole plate and

incubated for 10 minutes. The degree of luminescence in each well were detected by plate reader (PerkinElmer, 2105).

Antibodies

The following primary antibodies were used for immunostaining, immunoblotting or flow cytometry: For immunostaining, ENPP1 (1:250, Abcam, ab40003); Vimentin (1:100, Abcam, ab45939); cardiac Troponin I (1:100, Abcam, ab47003); cardiac Troponin I (1:200, Abcam, ab56357); CD31 (1:100, Abcam, ab7388); CD68 (1:50, Abcam, ab125212); gamma H2A.X(phospho S139) (1:100, Abcam, ab2893); Phosphp P53(Ser15) (1:100, Abcam, ab1431); cleaved-caspase 3 (1:200, Abcam, ab13847); Ki67 (1:100, Abcam, ab16667); alpha smooth muscle actin (1:100, Invitrogen, MA106110). For immunoblotting, ENPP1 (1:1000, CST, 2061S); gamma H2A.X(phospho S139) (1:1000, Abcam, ab26350); Phospho-Chk1(Ser345) (1:1000, CST, 2348); PhosphoP53(Ser15) (1:1000, Abcam, ab1431); P53 (1:500, Abcam, ab31333); GAPDH (1:1000, Millipore, ABS16). For flowcytometry: ENPP1-APC (1:200, eBiolegend, 149205); ENPP1-PE (1:200, eBiolegend, 149203); CD90.2-APC (1:200, eBioscience, 17-0902-81); MEFSK4-APC (1:20, Miltenyl Biotec, 130-102-302); CD31-APC (1:100, eBioscience, 17-0311-80); CD102-FITC (1:50, Biolegend, 105606); CD68-APC (1:50, Biolegend, 137007); CD14-APC (1:100, eBioscience, 17-0141-81); F4/80-APC (1:100, Biolegend, 123115).

Flow cytometry of the non-myocyte fraction from the injured heart.

Uninjured and injured regions of hearts from wild type C57BL6 mice or TCF21-MerCreMer:R26R^{tdTomato} mice were harvested at day 7 post MI. Tissues were rinsed in ice cold HBSS and then chopped into 1mm square pieces, digested in 0.1μg/ml liberase TH for 30 min at 37˚C. Digested hearts were filtered with a 40μm cell strainer (Fisher, 22363547), centrifuged at 200g for 5 min to pellet non-myocytes that

were resuspended in 1% BSA. $1x10^6$ non-myocytes from wild type or TCF21MCM: $R26R^{tdTomato}$ mice suspended in 100 µL 1%BSA were incubated with ENPP1 antibody and appropriate antibodies for fibroblasts (CD90.2, MEFSK4), endothelial cells (CD102 and CD31) or macrophages (CD68, CD14 and F4/80) for 30 minutes. After incubation, cells were resuspended in 1% BSA for flow cytometry. BD LSRII flow cytometer was used for all flow cytometry experiments. Data was analyzed using Flowjo software.

Traction force microscopy (TFM) measurements of cardiomyocyte contractility

The bio-sensor devices were fabricated by first spin coating a 75µm-thick polydimethylsiloxane (PDMS) onto a glass slide (200 RPM, 60s followed by 300 RPM, 60s) and baked at 60 Celsius for 12 hours. Before cell seeding, dopamine (0.01% ,w/v in 1M Tris-HCl buffer) and Geltrex (at concentration of 83 µL/mL) were coated for 1 hour on the surface of the PDMS devices respectively. NRVMs were seeded at 60-80% confluence on device. After treating the cells with the Vehicle or ENPP1+ATP conditioned medium for 24 hours, the cells were imaged under dark field microscopy (Zeiss AxioScope A1, EC Epiplan-Neofluar, 20x, N.A.=0.5) and contractility measurements determined as decribed by us[4,18].

Supplemental References

1. Pillai, I.C.L., et al., *Cardiac Fibroblasts Adopt Osteogenic Fates and Can Be Targeted to Attenuate Pathological Heart Calcification.* Cell Stem Cell, 2017. **20**(2): p. 218-+.
2. Ubil, E., et al., *Mesenchymal-endothelial transition contributes to cardiac neovascularization.* Nature, 2014. **514**(7524): p. 585-90.
3. Wang, Z., et al., *The long noncoding RNA Chaer defines an epigenetic checkpoint in cardiac hypertrophy.* Nat Med, 2016. **22**(10): p. 1131-1139.
4. Yokota, T., et al., *Type V Collagen in Scar Tissue Regulates the Size of Scar after Heart Injury.* Cell, 2020. **182**(3): p. 545-562 e23.
5. Dobin, A., et al., *STAR: ultrafast universal RNA-seq aligner.* Bioinformatics, 2013. **29**(1): p. 15-21.
6. Love, M.I., W. Huber, and S. Anders, *Moderated estimation of fold change and dispersion for RNA-seq data with DESeq2.* Genome Biol, 2014. **15**(12): p. 550.
7. Kuleshov, M.V., et al., *Enrichr: a comprehensive gene set enrichment analysis web server 2016 update.* Nucleic Acids Res, 2016. **44**(W1): p. W90-7.
8. Stuart, T., et al., *Comprehensive Integration of Single-Cell Data.* Cell, 2019. **177**(7): p. 1888-1902 e21.
9. Buescher, J.M., et al., *A roadmap for interpreting (13)C metabolite labeling patterns from cells.* Curr Opin Biotechnol, 2015. **34**: p. 189-201.
10. Carreer, W.J., R.M. Flight, and H.N. Moseley, *A Computational Framework for High-Throughput Isotopic Natural Abundance Correction of Omics-Level Ultra-High Resolution FT-MS Datasets.* Metabolites, 2013. **3**(4).
11. Moseley, H.N., *Correcting for the effects of natural abundance in stable isotope resolved metabolomics experiments involving ultra-high resolution mass spectrometry.* BMC Bioinformatics, 2010. **11**: p. 139.
12. Pinz, I., et al., *An improved isolation procedure for adult mouse cardiomyocytes.* Cell Biochem Biophys, 2011. **61**(1): p. 93-101.
13. Lian, X., et al., *Robust cardiomyocyte differentiation from human pluripotent stem cells via temporal modulation of canonical Wnt signaling.* Proc Natl Acad Sci U S A, 2012. **109**(27): p. E1848-57.
14. Bon, P., et al., *Quadriwave lateral shearing interferometry for quantitative phase microscopy of living cells.* Opt Express, 2009. **17**(15): p. 13080-94.
15. Zangle, T.A., et al., *Quantifying Biomass Changes of Single CD8+T Cells during Antigen Specific Cytotoxicity.* Plos One, 2013. **8**(7).
16. Crocker, J.C. and D.G. Grier, *Methods of digital video microscopy for colloidal studies.* Journal of Colloid and Interface Science, 1996. **179**(1): p. 298-310.
17. Davies, H.G. and M.H.F. Wilkins, *Interference Microscopy and Mass Determination.* Nature, 1952. **169**(4300): p. 541-541.
18. Barer, R., *Interference Microscopy and Mass Determination.* Nature, 1952. **169**(4296): p. 366-367.
19. Wang, J., et al., *Isoproterenol-Induced Cardiac Diastolic Dysfunction in Mice: A Systems Genetics Analysis.* Front Cardiovasc Med, 2019. **6**: p. 100.
20. Rau, C.D., et al., *Systems Genetics Approach Identifies Gene Pathways and Adamts2 as Drivers of Isoproterenol-Induced Cardiac Hypertrophy and Cardiomyopathy in Mice.* Cell Syst, 2017. **4**(1): p. 121-128 e4.

Figure S1

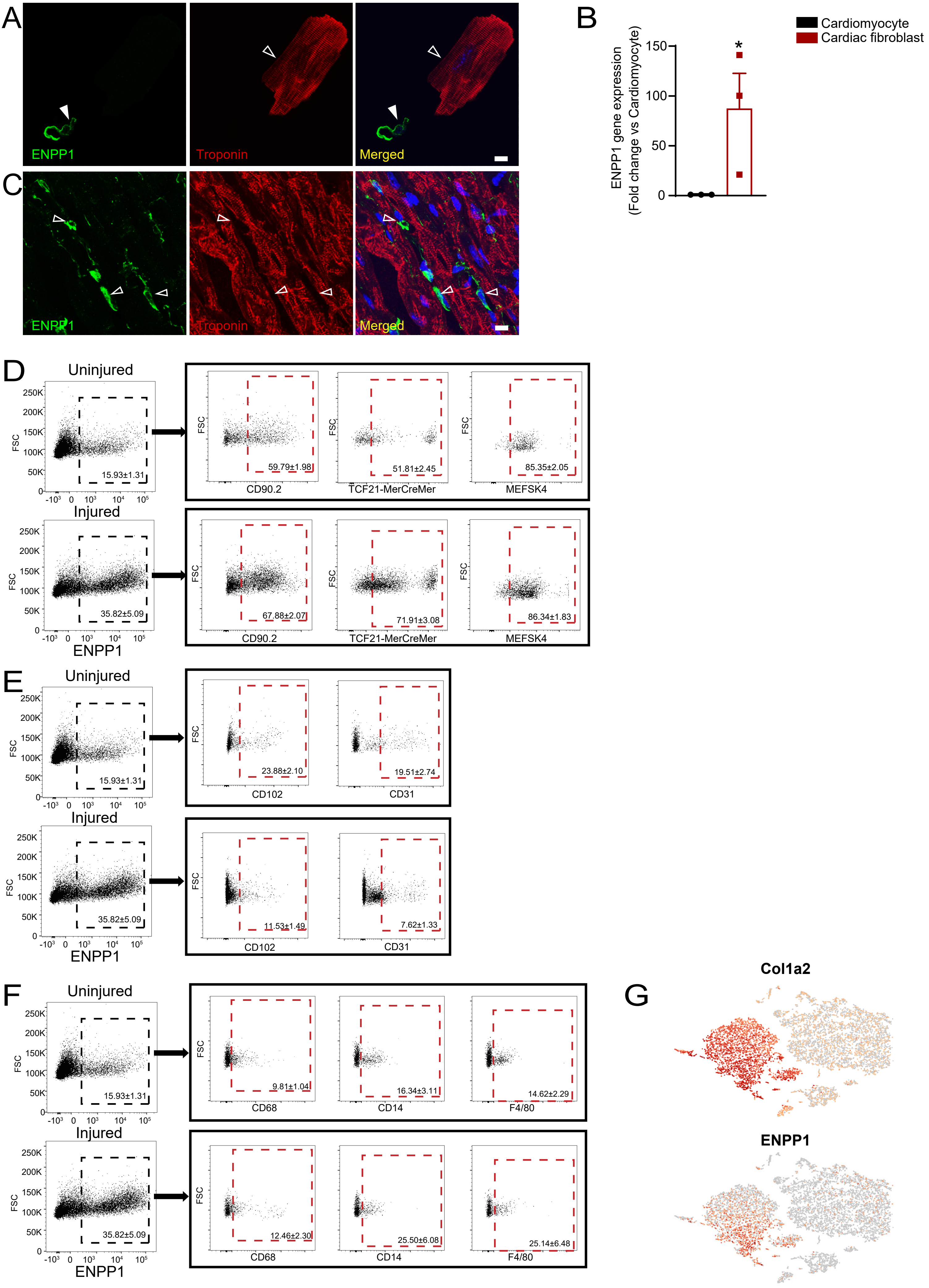
A
ENPP1
Troponin
Merged
B
ENPP1 gene expression
(Fold change vs Cardiomyocyte)
Cardiomyocyte
Cardiac fibroblast
*
C
ENPP1
Troponin
Merged
D
Uninjured
Injured
FSC
ENPP1
15.93±1.31
35.82±5.09
59.79±1.98
51.81±2.45
85.35±2.05
67.88±2.07
71.91±3.08
86.34±1.83
CD90.2
TCF21-MerCreMer
MEFSK4
E
Uninjured
Injured
23.88±2.10
19.51±2.74
11.53±1.49
7.62±1.33
CD102
CD31
F
Uninjured
Injured
9.81±1.04
16.34±3.11
14.62±2.29
12.46±2.30
25.50±6.08
25.14±6.48
CD68
CD14
F4/80
G
Col1a2
ENPP1

Figure S1. Expression of ENPP1 by non-myocyte cells in the injured heart. (A) Injured hearts were digested and myocytes isolated and stained with ENPP1 demonstrating lack of ENPP1 expression (green) by myocyte (stained red with troponin) Scale Bar: 10 μm. **(B)** qPCR demonstrating ENPP1 gene expression in non-myocytes and myocytes isolated from the same heart following injury (n=3, *p<0.05, Student's t-test, 2 tailed) **(C)** Immunostaining of hearts of injured animals (Day 7) demonstrating lack of colocalization of cardiac troponin (red, arrows) with ENPP1 expression (green, arrows) localized to interstitial cells (representative images, n=3) Scale Bar: 10 μm. **(D)** Flow cytometry of non-myocyte cells demonstrating increased number of ENPP1 expressing cells with injury and expression of ENPP1 cells primarily by cardiac fibroblasts (n=7/CD90.2, n=6/TCF21MerCreMer and n=6/MEFSK4). **(E)** Flow cytometry demonstrating expression of ENPP1 by endothelial cells in the uninjured and injured heart (n=8/CD102, n=5/CD31) and **(F)** expression of ENPP1 by macrophages (n=5/CD68, n=3/CD14 and n=3/F4/80). **(G)** Single cell RNA-seq demonstrating clusters of non-myocyte cells and overlapping expression of ENPP1 predominantly with the Col1a2 expressing cardiac fibroblasts (n=3). Data are expressed as mean ± S.E.M.

Figure S2

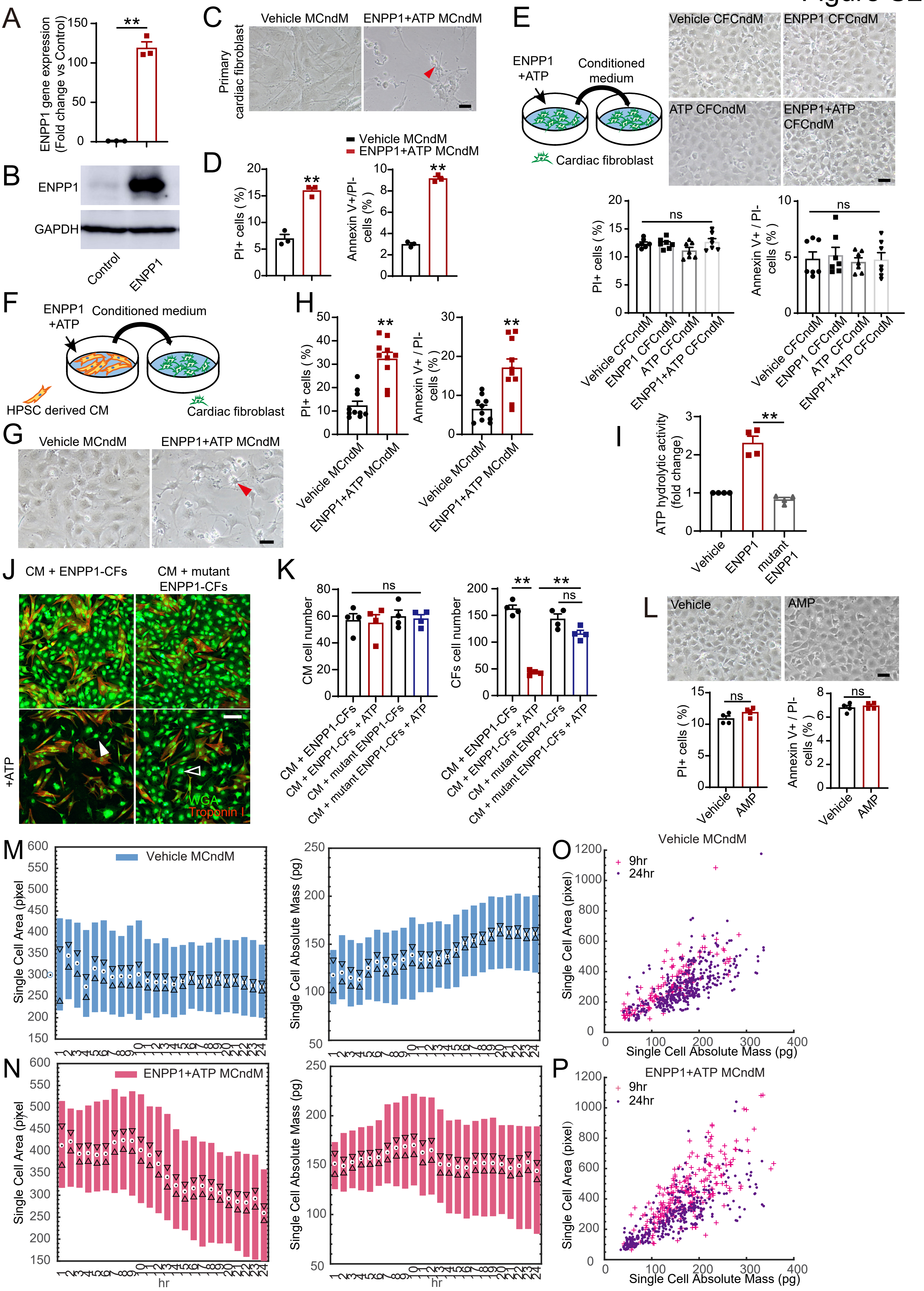

A
ENPP1 gene expression (Fold change vs Control)
**
B
ENPP1
GAPDH
Control
ENPP1
C
Vehicle MCndM
ENPP1+ATP MCndM
Primary cardiac fibroblast
D
Vehicle MCndM
ENPP1+ATP MCndM
PI+ cells (%)
Annexin V+/PI- cells (%)
E
ENPP1 +ATP
Conditioned medium
Cardiac fibroblast
Vehicle CFCndM
ENPP1 CFCndM
ATP CFCndM
ENPP1+ATP CFCndM
ns
F
ENPP1 +ATP
Conditioned medium
HPSC derived CM
Cardiac fibroblast
G
Vehicle MCndM
ENPP1+ATP MCndM
H
PI+ cells (%)
Annexin V+ / PI- cells (%)
Vehicle MCndM
ENPP1+ATP MCndM
I
ATP hydrolytic activity (fold change)
Vehicle
ENPP1
mutant ENPP1
J
CM + ENPP1-CFs
CM + mutant ENPP1-CFs
+ATP
WGA
Troponin I
K
CM cell number
CFs cell number
CM + ENPP1-CFs
CM + ENPP1-CFs + ATP
CM + mutant ENPP1-CFs
CM + mutant ENPP1-CFs + ATP
L
Vehicle
AMP
PI+ cells (%)
Annexin V+ / PI- cells (%)
M
Vehicle MCndM
Single Cell Area (pixel)
Single Cell Absolute Mass (pg)
hr
N
ENPP1+ATP MCndM
O
Vehicle MCndM
9hr
24hr
Single Cell Absolute Mass (pg)
P
ENPP1+ATP MCndM

Figure S2. The myocyte secretes pro-apoptotic factors in response to ENPP1+ATP. **(A)** qPCR (n=3) and **(B)** western blot (n=3) demonstrating ENPP1 expression in immortalized mouse CFs stably expressing ENPP1 compared to control GFP expressing CFs **(C)** Cell death of primary mouse CFs (arrows) treated with vehicle myocyte conditioned medium (MCndM) or ENPP1+ATP MCndM and **(D)** quantitation of cell death by flow cytometry (n=3). Scale Bar: 50 µm. **(E)** Conditioned medium collected from CFs following addition of vehicle, ENPP1, ATP or ENPP1+ATP, added to cardiac fibroblasts grown separately and photomicrographs taken 48 hours later and quantitation of cell death (n=7). Scale Bar: 50 µm. **(F)** Human pluripotent stem cell derived cardiomyocytes treated with ENPP1+ATP followed by harvesting of conditioned medium to treat CFs demonstrating **(G)** cell death (red arrows) and **(H)** quantitation of cell death (n=10). Scale Bar: 50 µm. **(I)** Creation of a mutant ENPP1 construct that is unable to hydrolyze ATP (n=4) and **(J)** Co-culture of cardiomyocytes (CM) (red) with ENPP1-CF (green) or mutant ENPP1 over-expressing cardiac fibroblasts (mutant ENPP1-CF) (green) demonstrating attenuation of fibroblast death with mutant ENPP1-CF upon addition of ATP (filled and unfilled arrows) and **(K)** quantitation of cell death (n=4). Scale Bar: 100 µm. **(L)** Effect on CFs treated with AMP and quantitation of cell death (n=4). Scale Bar: 50 µm. **(M-P)** Quantitative phase microscopy (QPM) measurements of projected single cell area and biomass changes in CFs over 24 hours of treatment with **(M)** control MCndM or **(N)** ENPP1+ATP MCndM. **(O,P)** Quantitation of single cell area and cell biomass showing the change in cell area and size over time in **(O)** vehicle MCndM or **(P)** ENPP1+ATP MCndM treated CFs ($p<0.05$ for reduction in cell size (area) over time in P versus O). Data are expressed as mean ± S.E.M. **$p<0.01$, ns: not significant, Statistics was determined using Ordinary one-way ANOVA with Tukey's multiple comparison test **(E, I, K),** or Student's t-test, 2 tailed **(A, D, H, L).**

Figure S3

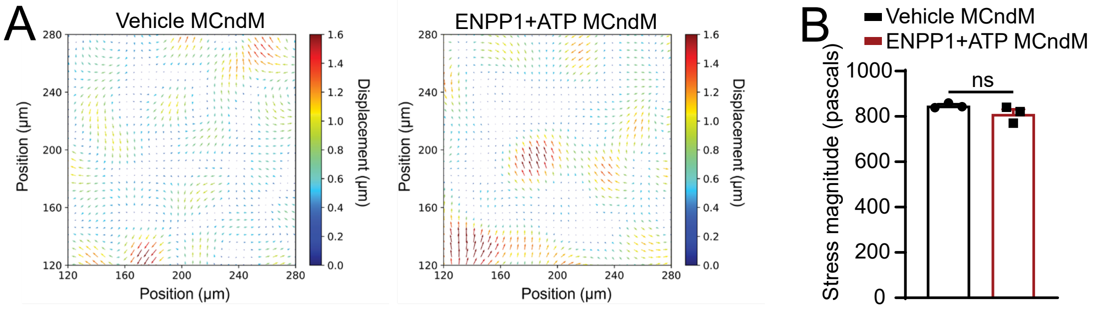

C

Term	Overlap	P- value
Citrate cycle (TCA cycle)	11/32	6.61E-05
Oxidative phosphorylation	15/134	6.39E-04
Parkinson disease	14/144	6.31E-04
Non- alcoholic fatty liver disease (NAFLD)	14/151	1.06E-04
Thermogenesis	18/231	1.14E-04
Glyoxylate and dicarboxylate metabolism	6/31	2.05E-04
p53 signaling pathway	1/71	0.871164

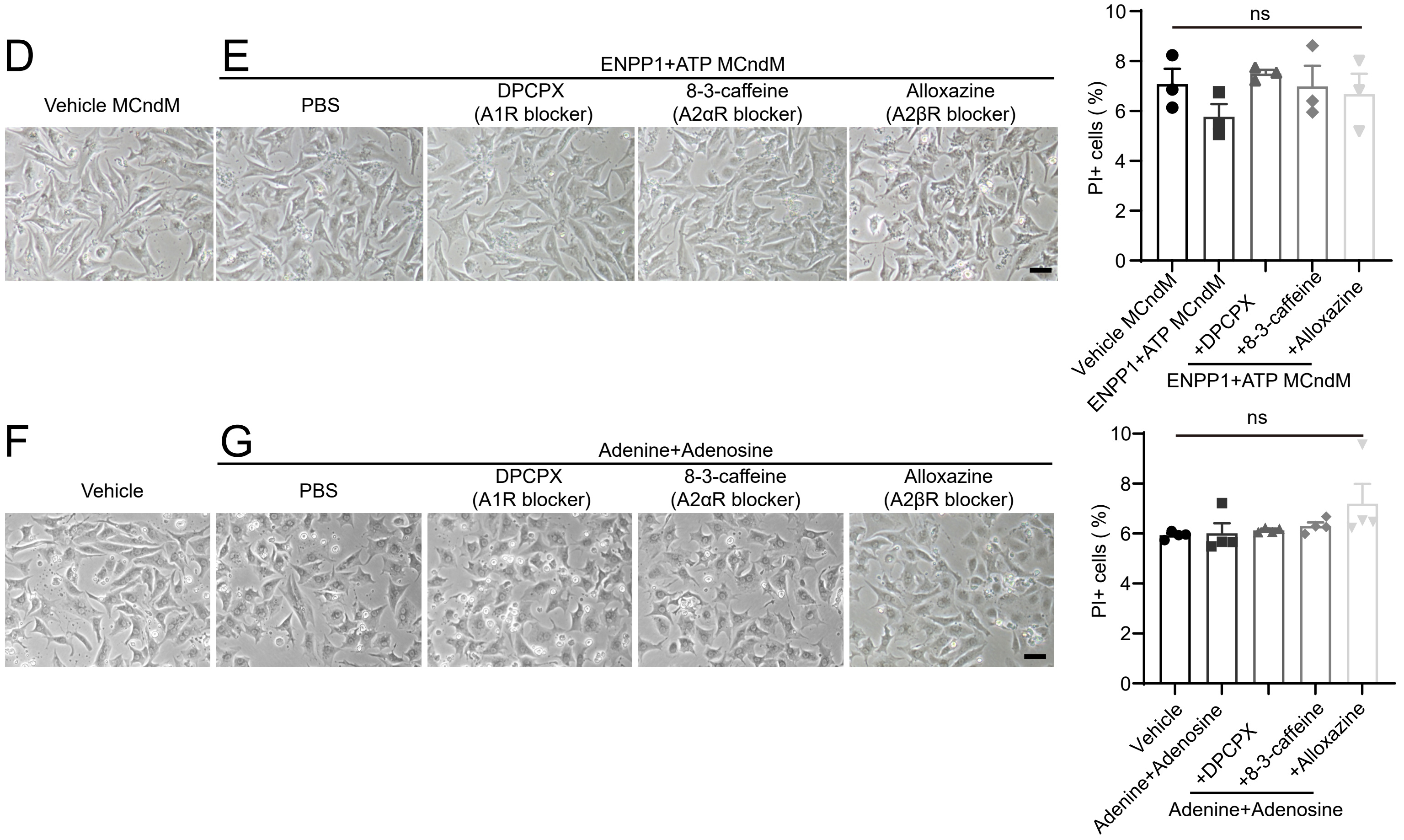

Figure S3. Effects of ENPP1+ATP MCndM on myocyte function. **(A,B)** Traction force microscopy demonstrating **(A)** Heat maps of myocyte contractility following treatment of cardiomyocytes with Vehicle or ENPP1+ATP MCnDM and **(B)** quantitation of force of contraction between 2 groups (n=3). **(C)** Cardiomyocytes were treated with ENPP1+ATP MCndM or vehicle MCndM and harvested 24 hours later for gene expression analysis. GO analysis of differentially expressed genes in ENPP1+ATP MCndM treated versus vehicle MCndM cardiomyocytes demonstrates pathways linked to metabolism but p53 driven apoptosis pathways were not significantly different. (**D,E**) Effect of adenosine receptor blockers on myocytes treated with ENPP1+ATP MCndM. Scale Bar: 50 μm. Cardiomyocytes were treated with **(D)** Vehicle MCndM or **(E)** ENPP1+ATP MCndM in the presence of PBS or three different adenosine receptor blockers followed by flow cytometry (PI) at 48 hours to determine cell death (n=3) **(F,G)** Effect of adenosine receptor blockers following treatment of cardiomyocytes with a combination of adenine and adenosine. Scale Bar: 50 μm. Cardiomyocytes were treated with **(F)** vehicle or **(G)** adenine and adenosine in the presence of PBS or three different adenosine receptor blockers followed by flow cytometry (PI) at 48 hours to determine cell death (n=4). Data are expressed as mean ± S.E.M. $^{**}p<0.01$, $^{*}p<0.05$, ns: not significant, Statistics was determined using Ordinary one-way ANOVA with Tukey's multiple comparison test **(E,G),** or Student's t-test, 2 tailed **(B).**

Figure S4

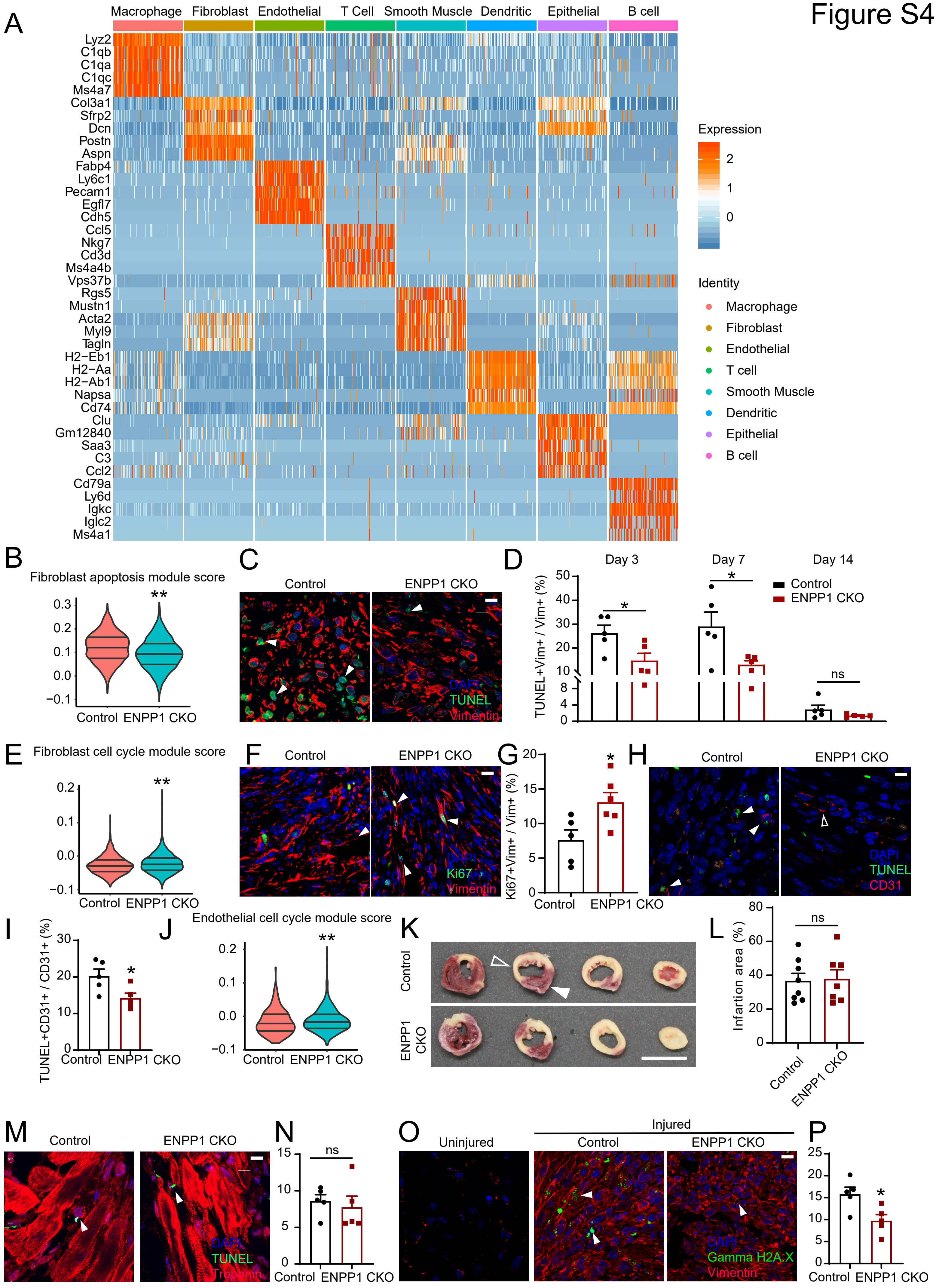
A
Macrophage
Fibroblast
Endothelial
T Cell
Smooth Muscle
Dendritic
Epithelial
B cell
Lyz2
C1qb
C1qa
C1qc
Ms4a7
Col3a1
Sfrp2
Dcn
Postn
Aspn
Fabp4
Ly6c1
Pecam1
Egfl7
Cdh5
Ccl5
Nkg7
Cd3d
Ms4a4b
Vps37b
Rgs5
Mustn1
Acta2
Myl9
Tagln
H2−Eb1
H2−Aa
H2−Ab1
Napsa
Cd74
Clu
Gm12840
Saa3
C3
Ccl2
Cd79a
Ly6d
Igkc
Iglc2
Ms4a1
Expression
2
1
0
Identity
Macrophage
Fibroblast
Endothelial
T cell
Smooth Muscle
Dendritic
Epithelial
B cell
B
Fibroblast apoptosis module score
**
Control ENPP1 CKO
C
Control
ENPP1 CKO
DAPI
TUNEL
Vimentin
D
Day 3
Day 7
Day 14
Control
ENPP1 CKO
TUNEL+Vim+ / Vim+ (%)
ns
E
Fibroblast cell cycle module score
**
Control ENPP1 CKO
F
Control
ENPP1 CKO
DAPI
Ki67
Vimentin
G
Ki67+Vim+ / Vim+ (%)
Control ENPP1 CKO
H
Control
ENPP1 CKO
DAPI
TUNEL
CD31
I
TUNEL+CD31+ / CD31+ (%)
Control ENPP1 CKO
J
Endothelial cell cycle module score
**
Control ENPP1 CKO
K
Control
ENPP1 CKO
L
Infartion area (%)
ns
Control
ENPP1 CKO
M
Control
ENPP1 CKO
DAPI
TUNEL
Troponin
N
ns
Control ENPP1 CKO
O
Injured
Uninjured
Control
ENPP1 CKO
DAPI
Gamma H2A.X
Vimentin
P
Control ENPP1 CKO

Figure S4. Cell death and cycling of non-myocytes and myocytes in control and ENPP1 CKO animals after ischemic cardiac injury. (A) Canonical genes used to characterize various non-myocyte cell phenotypes on single cell RNA-seq of non-myocytes at 7 days post injury. **(B)** Analysis of expression of apoptotic genes (apoptosis module score) from single cell RNA-seq in control and ENPP1CKO animals at 7 days after injury shows lower apoptotic signatures in ENPP1CKO CFs (module of 124 genes, **p=2.2e-16) **(C)**TUNEL staining showing TUNEL+ apoptotic CFs in control and ENPP1CKO hearts at 7 days after injury (arrows, representative images) and **(D)** Quantitation of apoptotic CFs at 3,7 and 14 days after cardiac injury in control and ENPP1CKO hearts (n=5). Scale bar: 5 µm. **(E)** Analysis of expression of cell cycling genes in CFs used to calculate a cell cycling module score in cardiac fibroblasts of control and ENPP1CKO hearts at 7 days after injury shows higher cell cycling score in ENPP1CKO cardiac fibroblasts (module of 97 genes, *p=2.093e-10) (**F,G**) Immunostaining for **(F)** Ki67 and vimentin (fibroblast marker) in control and ENPP1CKO hearts at 7 days after cardiac injury and **(G)** quantitation of Ki67+ CFs (n=5/6). Scale bar: 10 µm. **(H,I)** TUNEL staining showing **(H)** TUNEL+ apoptotic endothelial cells in control and ENPP1CKO hearts at 7 days after injury (arrows, representative images) and **(I)** quantitation (n=5). Scale bar: 5 µm. **(J)** Analysis of expression of cell cycling genes in endothelial cells used to calculate a cell cycling module score in cardiac endothelial cells of control and ENPP1CKO hearts at 7 days after injury shows higher cell cycling score in ENPP1CKO endothelial cells (module of 97 genes, **p=0.032). **(K)** TTC staining demonstrating viable (solid arrow) and dead myocardium (unfilled arrow) in control and ENPP1CKO animals 24 hours after ischemic cardiac injury (sections taken from base to apex of infarct) and **(L)** quantitation of infarct area from TTC staining (n=8/7). **(M)** TUNEL staining demonstrating apoptotic myocytes (identified by troponin) in hearts of control and ENPP1CKO animals at 72 hours after injury (representative images, arrows) and **(N)** quantitation (n=5/group). Scale bar: 5 µm. **(O,P)** gamma H2A.X and vimentin immunostaining in **(O)** uninjured control, Injured control and injured ENPP1CKO hearts demonstrates increased number of vimentin + cells (CFs) expressing gamma H2A.X following injury and significant decrease in vimentin + cells (CFs) expressing gamma H2A.X in ENPP1CKO hearts and **(P)** quantitation of the number of gamma H2A.X expressing CFs (n=5). Scale bar: 5 µm. Data are expressed as mean ± S.E.M. **p<0.01, *p<0.05, ns: not significant, Statistics was determined using Ordinary one-way ANOVA with Tukey's multiple comparison test **(B),** or Student's t-test, 2 tailed **(G, I, J, K).**

Figure S5

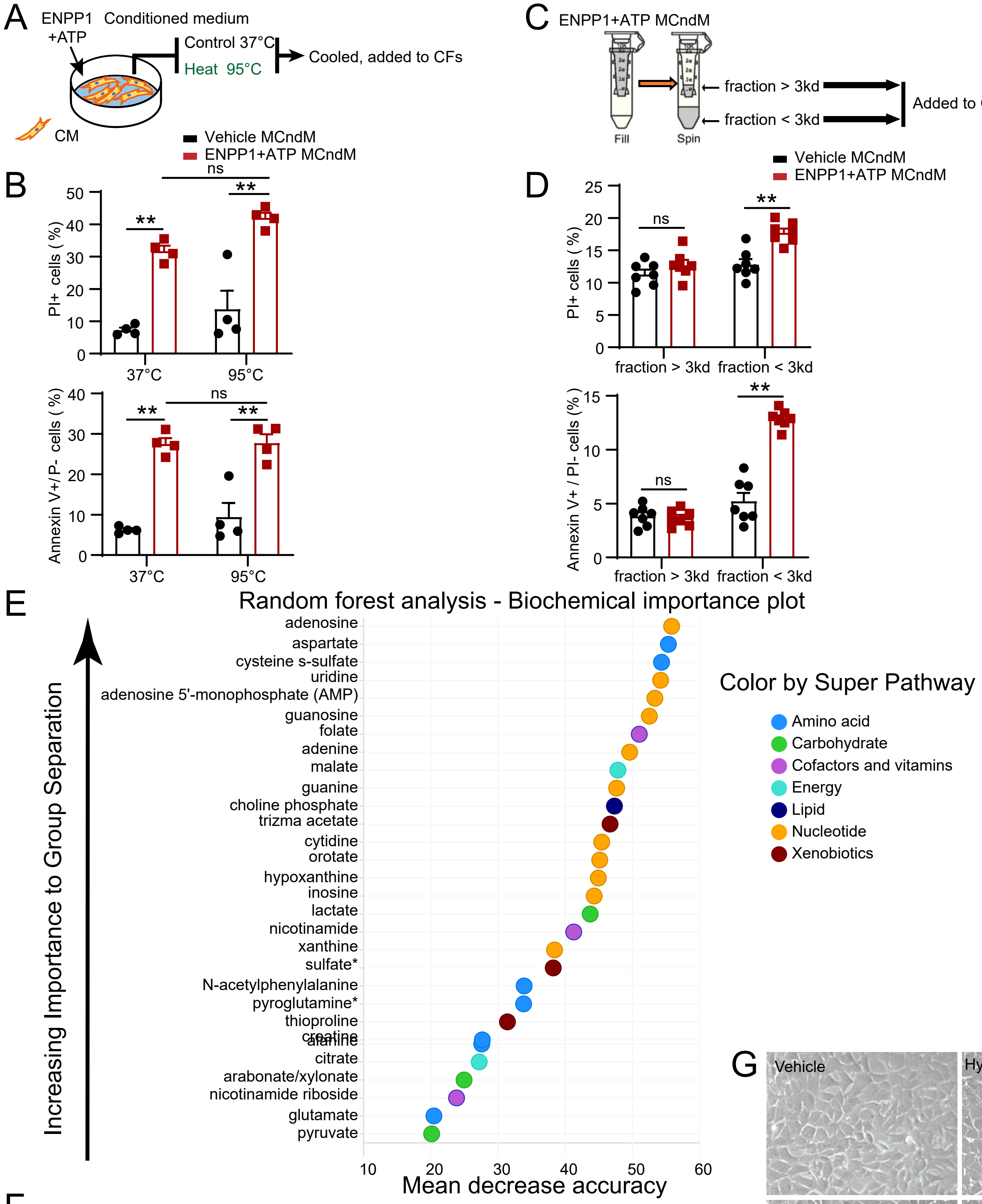

F

Super Pathway	Sub Pathway	Biochemical Name	Fold change (p<0.05) (ENPP1+ATP) / Vehicle	AMP/Vehicle
Nucleotide	Purine Metabolism, (Hypo)Xanthine/Inosine containing	inosine	33.28	34.13
		hypoxanthine	122.09	151.21
		xanthine	2.05	3.39
	Purine Metabolism, Adenine containing	adenosine 5'-monophosphate (AMP)	4.12	1.88
		adenosine	1081.44	1455.28
		adenine	66.66	93.63
	Purine Metabolism, Guanine containing	guanosine	1.64	1.11
		guanine	1.69	2.07
	Pyrimidine Metabolism, Orotate containing	orotate	30.10	31.06
	Pyrimidine Metabolism, Uracil containing	uridine	0.53	0.44
	Pyrimidine Metabolism, Cytidine containing	cytidine	0.17	0.17

G

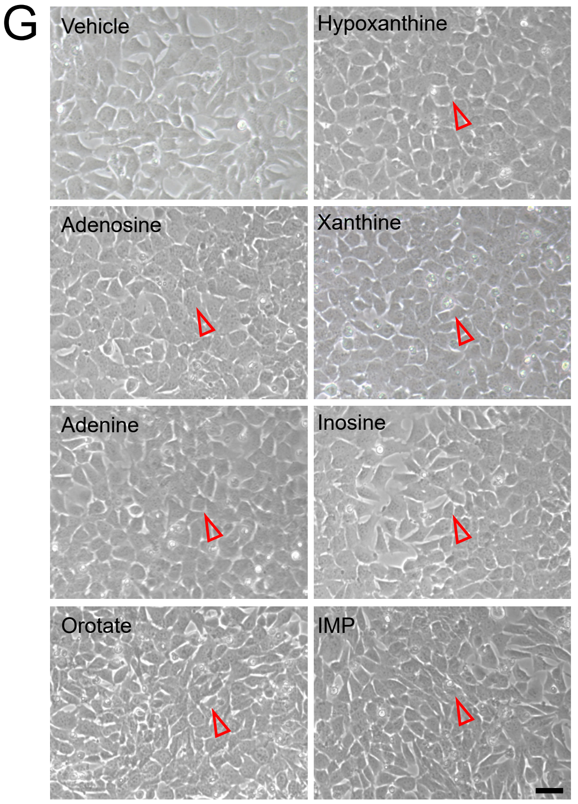

Figure S5. The pro-apoptotic molecules secreted by cardiomyocytes in response to ENPP1+ATP are likely to be metabolites rather than proteins. (A) ENPP1+ATP MCndM was subjected to high heat treatment (95ºC), cooled and then added to CFs **(B)** Quantitation of cell death caused by vehicle MCndM or ENPP1+ATP MCndM with/without heat inactivation (n=4). **(C)** ENPP1+ATP MCndM was subjected to a protein fractionation column and separated into protein poor (<3kD) and protein rich (>3kD) fractions and added to CFs and **(D)** quantitation of cell death of CFs demonstrating that the protein poor fraction retains cell death inducing biological activity (n=7). **(E)** LC/MS demonstrating the top differentially present metabolites versus predominant pathway implicated in ENPP1+ATP MCndM or AMP MCndM compared to vehicle MCndM (n=6 for each group). **(F)** Fold changes in the top differentially present metabolites in ENPP1+ATP or AMP MCndM compared to vehicle MCndM. **(G)** Effects of adding each of the top differentially present compounds on cardiac fibroblasts demonstrating no effect on cell viability (unfilled arrows). Scale Bar: 50 μm. Data are expressed as mean ± S.E.M. $^{**}p<0.01$, ns: not significant, Statistics was determined using Ordinary one-way ANOVA with Tukey's multiple comparison test **(B, D).**

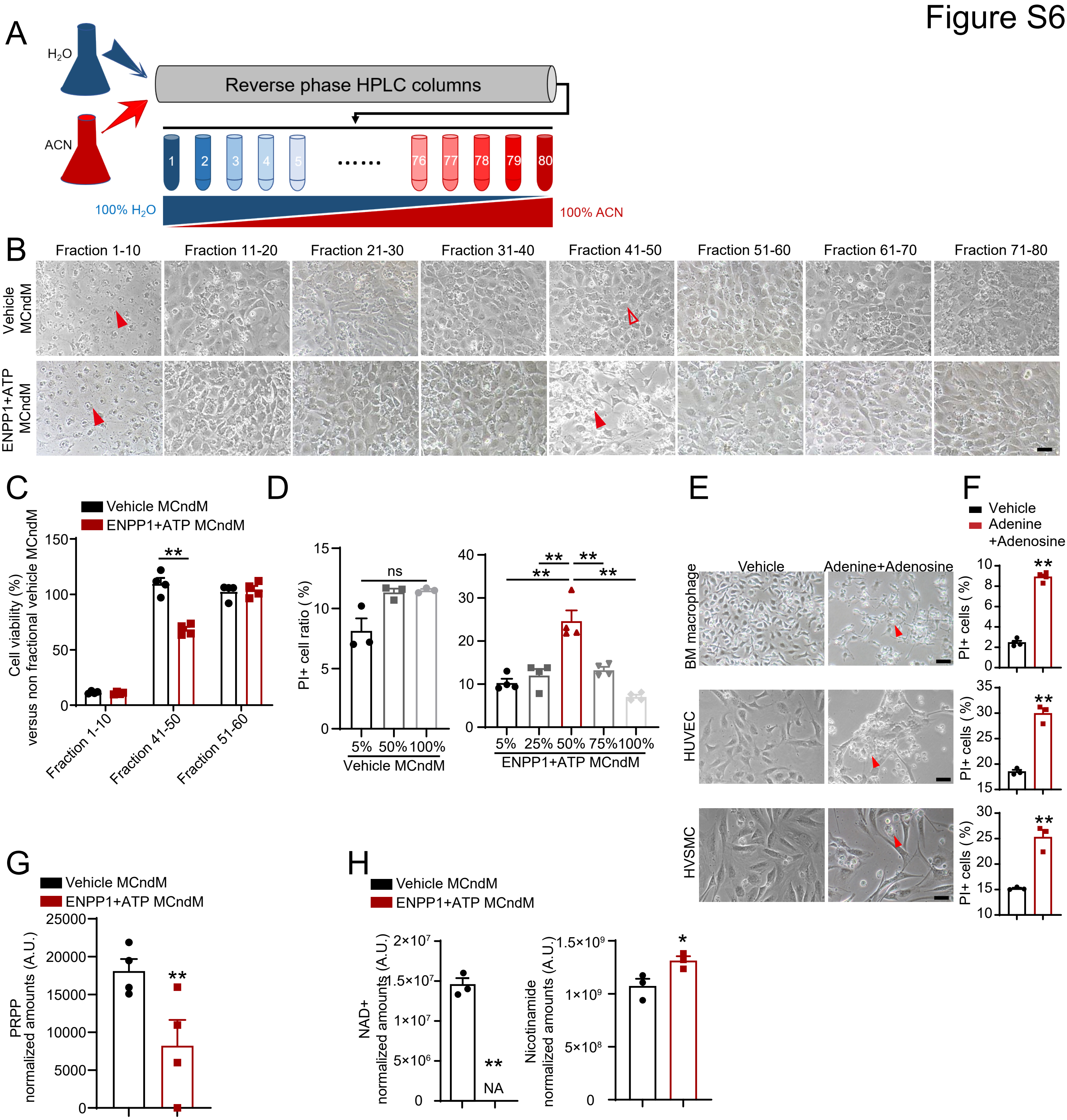
Figure S6
A
H2O
ACN
Reverse phase HPLC columns
100% H2O
100% ACN
B
Fraction 1-10
Fraction 11-20
Fraction 21-30
Fraction 31-40
Fraction 41-50
Fraction 51-60
Fraction 61-70
Fraction 71-80
Vehicle MCndM
ENPP1+ATP MCndM
C
Vehicle MCndM
ENPP1+ATP MCndM
Cell viability (%) versus non fractional vehicle MCndM
D
PI+ cell ratio (%)
ns
5% 50% 100%
Vehicle MCndM
5% 25% 50% 75% 100%
ENPP1+ATP MCndM
E
Vehicle
Adenine+Adenosine
BM macrophage
HUVEC
HVSMC
F
Vehicle
Adenine +Adenosine
PI+ cells (%)
G
Vehicle MCndM
ENPP1+ATP MCndM
PRPP normalized amounts (A.U.)
H
Vehicle MCndM
ENPP1+ATP MCndM
NAD+ normalized amounts (A.U.)
NA
Nicotinamide normalized amounts (A.U.)

Figure S6. High performance liquid chromatography (HPLC) with acetonitrile elutions to determine identity of pro-apoptotic compounds secreted by myocytes in response to ENPP1+ATP. (A) High performance liquid chromatography (HPLC) performed with two reversed phase HPLC columns connected in series. ENPP1+ATP MCndM or vehicle MCndM was loaded onto the columns with linear increase in acetonitrile as the eluting solvent and 80 fractions were collected for determining the eluates with cell death inducing biological activity. **(B)** Photomicrographs of CFs 48 hours following treatment with different fractions eluted above demonstrating cell death inducing properties of eluates 41-50 (arrows) with ENPP1+ATP MCndM versus no cell death with corresponding eluates with vehicle MCndM (unfilled arrows). Scale Bar: 50 µm. **(C)** Cell viability assay confirming cell death inducing properties of eluates 41-50 of MCndM compared to corresponding elutes of vehicle MCndM (n=4). Note eluates 1-10 of both vehicle and ENPP1+ATP MCndM induced cell death and so were ignored. **(D)** ENPP1+ATP MCndM or vehicle MCndM was loaded onto a C18 column and then fractions collected following elution with 5, 25, 50, 75 and 100% of ACN, vacuum dried, reconstituted and then added to CFs and cell death measured with PI staining on flow cytometry (n=4). **(E)** Effects of cell death on adding adenine + adenosine to BM macrophages, HUVECs and hVSMC and **(F)** quantitation of cell death (n=3) Scale Bar: 50 µm. **(G)** decreased PRPP levels in cardiac fibroblasts treated with ENPP1+ATP MCndM (n=4). **(H)** Metabolomic analysis demonstrates decreased intracellular NAD+ levels and increased nicotinamide levels in CFs treated with vehicle or ENPP1+ATP MCndM (n=3). Data are expressed as mean ± S.E.M. **p<0.01, *p<0.05, ns: not significant, Statistics was determined using Ordinary one-way ANOVA with Tukey's multiple comparison test **(D),** or Student's t-test, 2 tailed **(C, F, G, H).**

Figure S7

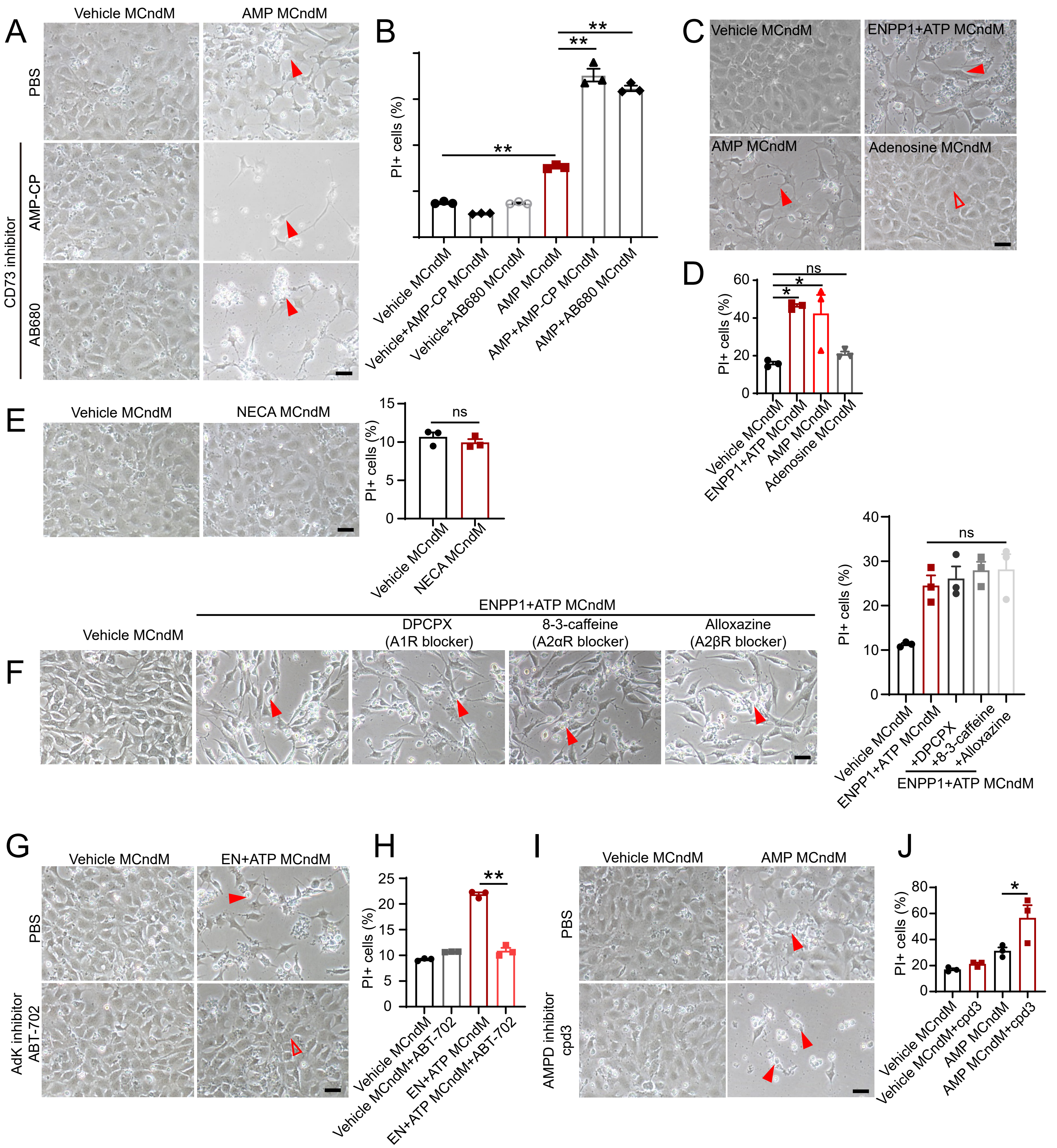
A
Vehicle MCndM
AMP MCndM
PBS
CD73 inhibitor
AMP-CP
AB680
B
PI+ cells (%)
Vehicle MCndM
Vehicle+AMP-CP MCndM
Vehicle+AB680 MCndM
AMP MCndM
AMP+AMP-CP MCndM
AMP+AB680 MCndM
C
Vehicle MCndM
ENPP1+ATP MCndM
AMP MCndM
Adenosine MCndM
D
PI+ cells (%)
ns
Vehicle MCndM
ENPP1+ATP MCndM
AMP MCndM
Adenosine MCndM
E
Vehicle MCndM
NECA MCndM
PI+ cells (%)
ns
F
ENPP1+ATP MCndM
Vehicle MCndM
DPCPX
(A1R blocker)
8-3-caffeine
(A2αR blocker)
Alloxazine
(A2βR blocker)
PI+ cells (%)
ns
+DPCPX
+8-3-caffeine
+Alloxazine
G
Vehicle MCndM
EN+ATP MCndM
PBS
AdK inhibitor
ABT-702
H
PI+ cells (%)
Vehicle MCndM+ABT-702
EN+ATP MCndM
EN+ATP MCndM+ABT-702
I
AMPD inhibitor
cpd3
J
Vehicle MCndM+cpd3
AMP MCndM+cpd3

Figure S7. AMP and not adenosine is critical for the myocyte to generate pro-apoptotic molecules. (A) CFs treated with vehicle MCndM or AMP MCndM in the absence or presence of CD73 inhibitors (AMP-CP and AB680) (the inhibitors were added to the cardiomyocytes at the time of addition of ENPP1+ATP and then the conditioned medium collected for treating cardiac fibroblasts) demonstrating increased cell death when CD73 inhibitor was added (arrows) **(B)** Quantitation of cell death (n=3). Scale Bar: 50 µm. (**C**) Cardiac fibroblasts treated with vehicle, ENPP1+ATP, AMP or adenosine MCndM and photomicrographs taken 48 hours later and **(D)** quantitation of cell death demonstrating an inability of adenosine MCndM (unfilled arrow) to cause cell death (n=3). Scale Bar: 50 µm. **(E)** Addition of adenosine receptor agonist NECA to cardiomyocytes followed by collection and treatment of CFs with NECA MCndM and quantitation of cell death in CFs treated with NECA MCndM (n=3). Scale Bar: 50 µm. **(F)** CFs treated with vehicle MCndM or ENPP1+ATP MCndM in the presence or absence of adenosine receptor antagonists (adenosine receptor antagonists were added to cardiomyocytes at the time of addition of ENPP1+ATP) and quantitation of cell death (n=3). Scale Bar: 50 µm. **(G)** CFs treated with vehicle or ENPP1+ATP MCndM in the presence of adenosine kinase (Adk) inhibitor (ABT-702) with attenuation of cell death in the presence of the inhibitor (filled and unfilled arrows) (Adk inhibitor was added to cardiomyocytes at the time of adding ENPP1+ATP) and **(H)** quantitation of cell death under these conditions (n=3). Scale Bar: 50 µm. **(I)** CFs treated with vehicle or ENPP1+ATP MCndM in the presence of AMP deaminase (AMPD) inhibitor (cpd3) (AMPD inhibitor was added to cardiomyocytes at the time of adding ENPP1+ATP), demonstrates increased cell death (arrows) and **(J)** quantitation of cell death (n=3). Scale Bar: 50 µm. Data are expressed as mean ± S.E.M. $^{**}p<0.01$, $^{*}p<0.05$, ns: not significant, Statistics was determined using Ordinary one-way ANOVA with Tukey's multiple comparison test **(B, D, F, H, J),** or Student's t-test, 2 tailed **(E).**

Figure S8

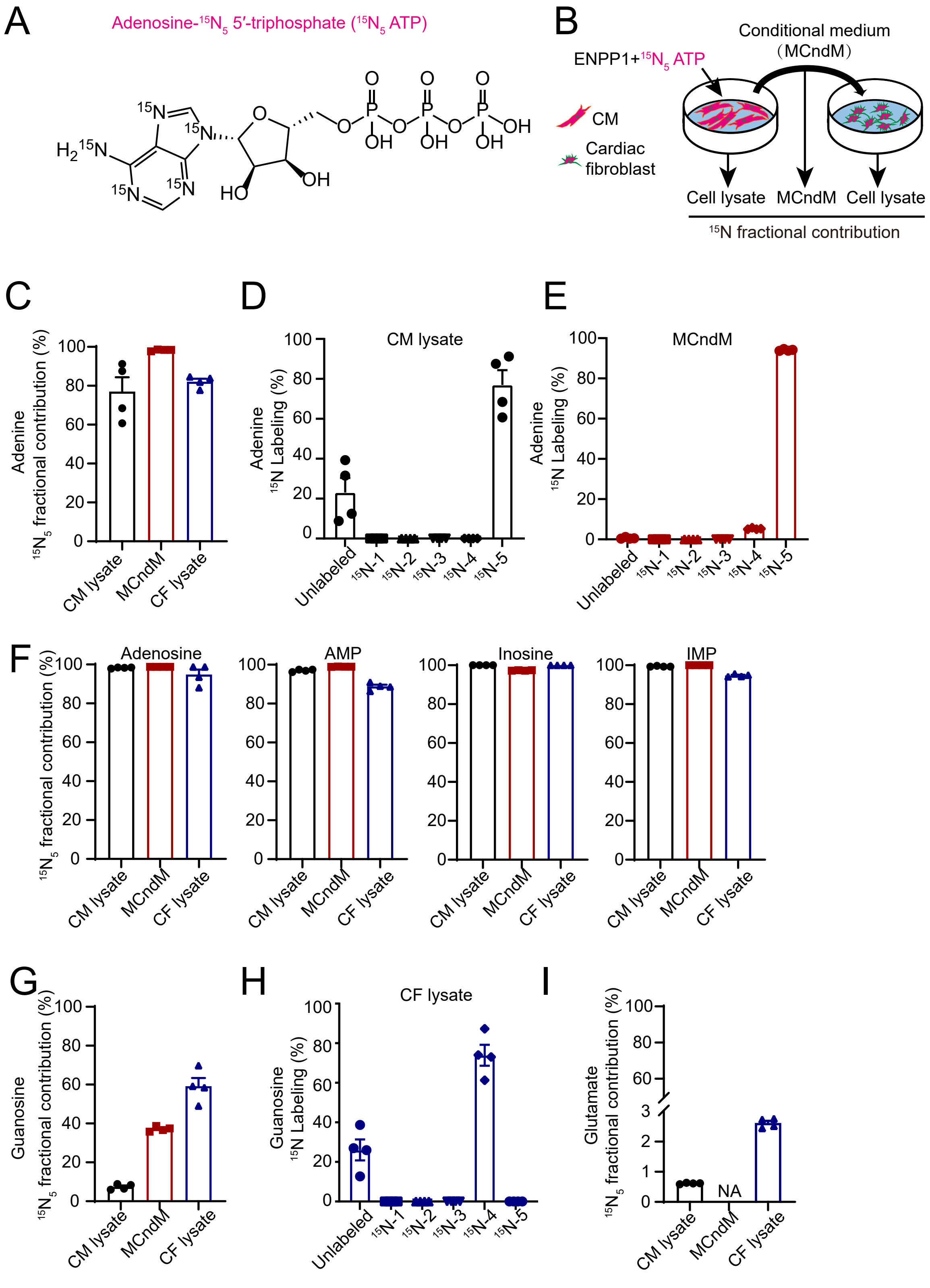
A
Adenosine-15N5 5′-triphosphate (15N5 ATP)
B
ENPP1+15N5 ATP
Conditional medium (MCndM)
CM
Cardiac fibroblast
Cell lysate MCndM Cell lysate
15N fractional contribution
C
Adenine 15N5 fractional contribution (%)
CM lysate
MCndM
CF lysate
D
CM lysate
Adenine 15N Labeling (%)
Unlabeled
15N-1
15N-2
15N-3
15N-4
15N-5
E
MCndM
Adenine 15N Labeling (%)
F
Adenosine
AMP
Inosine
IMP
15N5 fractional contribution (%)
G
Guanosine 15N5 fractional contribution (%)
H
CF lysate
Guanosine 15N Labeling (%)
I
Glutamate 15N5 fractional contribution (%)
NA

Figure S8. N15ATP labeling experiments demonstrate that AMP is directly used by the cardiomyocyte to generate adenine which is then taken up cardiac fibroblasts. (A) $15N_5$-labeled ATP with all 5 nitrogen atoms labeled ($^{15}N_5$) **(B)** Experimental design where ENPP1+$15N_5$ATP was added to cardiomyocytes and the conditioned medium collected and added to CFs. Cell lysates from cardiomyocytes, the conditioned medium and cell lysates from treated CFs were then subjected to LC/MS to determine the fraction of labeled metabolites (n=4). **(C)** Fraction of labeled adenine in the cardiomyocyte, conditioned medium and CFs (n=4) and **(D,E)** Isotopologue distribution (the relative intensities of isotopologues containing the indicated number of labeled nitrogen atoms) demonstrates that adenine both in the **(D)** cardiomyocytes and in the **(E)** conditioned media (MCndM) is predominantly highly labeled. **(F)** Fractional contribution of heavy nitrogen to adenosine, AMP, inosine and IMP in the myocyte, conditioned medium and CFs (n=4)**. (G)** Fractional contribution of heavy nitrogen to guanosine in cardiac myocyte, conditioned medium and CFs and **(H)** the corresponding isotopologue distribution demonstrates 4 heavy nitrogen atoms as the predominantly labeled form, as expected from four nitrogens being provided by XMP and one nitrogen being contributed from glutamine. **(I)** Low levels of labeling of glutamate, a metabolite not typically derived from adenosine precursors, as a negative labeling example. Data are expressed as mean ± S.E.M.

Figure S9

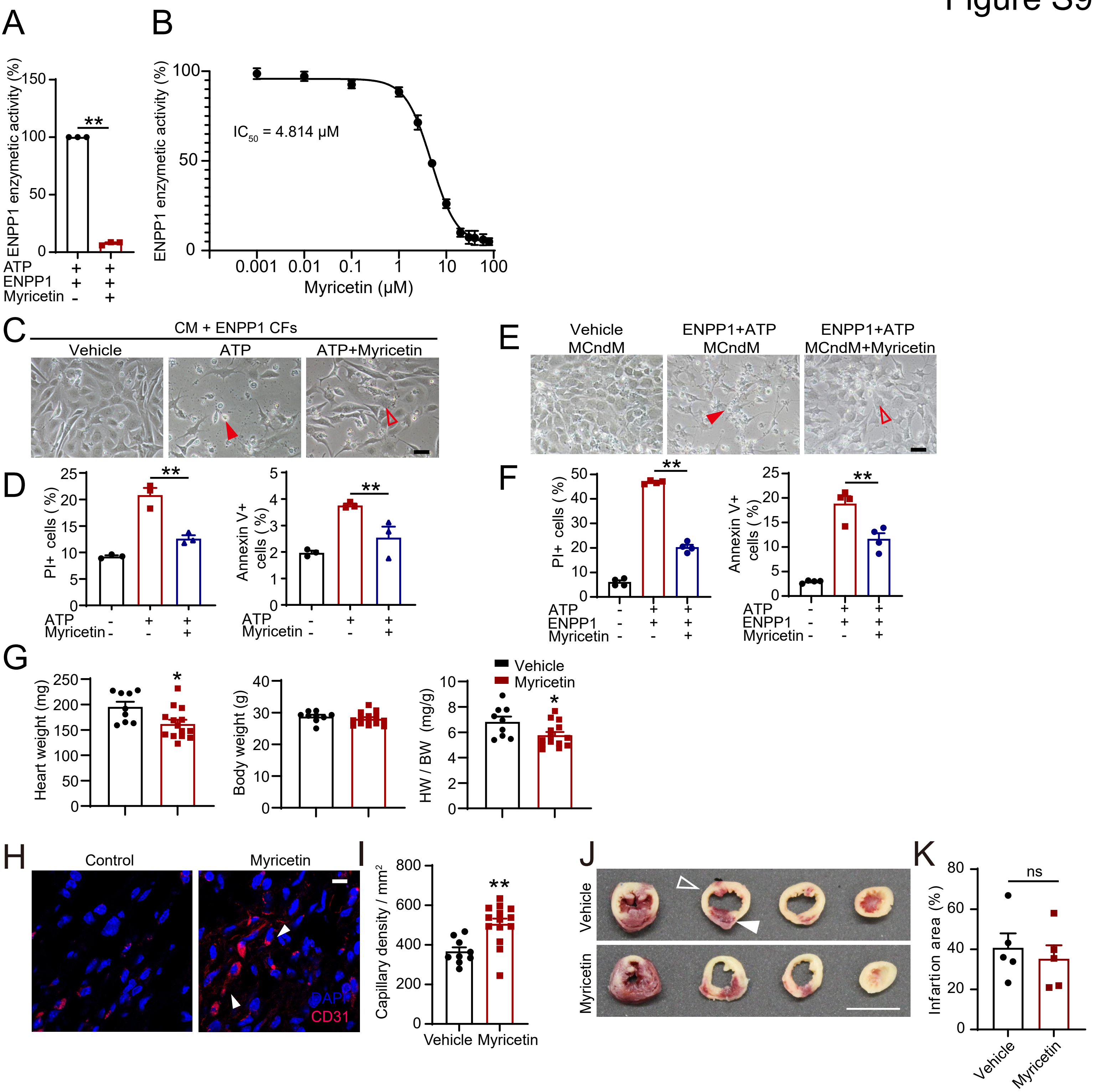

A
B
ENPP1 enzymetic activity (%)
IC50 = 4.814 μM
Myricetin (μM)
ATP
ENPP1
Myricetin
C
CM + ENPP1 CFs
Vehicle
ATP
ATP+Myricetin
D
PI+ cells (%)
Annexin V+ cells (%)
E
Vehicle MCndM
ENPP1+ATP MCndM
ENPP1+ATP MCndM+Myricetin
F
G
Heart weight (mg)
Body weight (g)
HW / BW (mg/g)
Vehicle
Myricetin
H
Control
Myricetin
DAPI
CD31
I
Capillary density / mm2
J
K
Infartion area (%)
ns

Figure S9. Identification of a small molecule that robustly inhibits ENPP1. (**A)** Myricetin robustly inhibits ENPP1 activity (n=3) **(B)** IC_{50} curve of myricetin (n=3) **(C)** Co-culture of ENPP1 overexpressing CFs and myocytes treated with vehicle, ATP or with myricetin (10uM) demonstrating attenuation of cell death with myricetin (arrows, unfilled arrows) and **(D)** Quantitation of effects of myricetin in attenuating cell death (n=3). Scale Bar: 50 µm. **(E)** CFs were treated with ENPP1+ATP MCndM in the absence or presence of myricetin (10uM) (Myricetin was added to the myocytes at the time of adding ENPP1 and ATP) and photomicrographs taken 48 hours later demonstrated greater viability of cardiac fibroblasts (filled and unfilled arrows) **(F)** quantitation of the effects of myricetin on cell death (n=4). Scale Bar: 50 µm. **(G)** Heart weight, body weight and HW/BW ratio in vehicle and myricetin treated animals at 4 weeks after heart injury (n=9/vehicle and 14/uridine). (**H**) Immunostaining demonstrating capillary counts (arrows) in vehicle or myricetin treated animals and **(I)** Quantitation (n=9/vehicle and 14/myricetin). Scale Bar: 10 µm. **(J)** TTC staining demonstrating viable (solid arrow) and dead myocardium (unfilled arrow) in vehicle and myricetin injected animals 24 hours after ischemic cardiac injury (sections taken from base to apex of infarct) and **(K)** quantitation of infarct area from TTC staining (n=5/group). Scale Bar: 500 µm. Data are expressed as mean ± S.E.M. $^{**}p<0.01$, $^{*}p<0.05$, ns: not significant, Statistics was determined using Ordinary one-way ANOVA with Tukey's multiple comparison test **(D, F),** or Student's t-test, 2 tailed **(A, G, I, K).**

Table S1. Contingency table demonstrating numbers of animals with mild, moderate or severe impairment of contractile cardiac function or fibrosis in ENPP1CKO versus control or following administration of uridine or myricetin. Contingency tables demonstrating numbers of animals with **(A)** mild, moderate or severe reduction in ejection fraction at 4 weeks after cardiac injury in ENPP1CKO versus control and uridine or myricetin versus vehicle groups and with **(B)** mild, moderate or severe fibrotic scar size in ENPP1 CKO versus control and uridine or myricetin treated groups versus vehicle.

A

	<20%	20%-40%	>40%	Total
Control	7	4	3	14
ENPP1 CKO	1	5	10	16

χ^2 =8.284, p value =0.016

	<20%	20%-40%	>40%	Total
Vehicle	10	4	0	14
Uridine	2	9	4	15

χ^2 =11.24, p value =0.004

	<20%	20%-40%	>40%	Total
Vehicle	6	3	0	9
Myricetin	3	6	5	14

χ^2 =6.206, p value =0.045

B

	Mild	Moderate	Severe	Total
Control	6	5	3	14
ENPP1 CKO	11	4	1	16

χ^2 =2.459, p value =0.292

	Mild	Moderate	Severe	Total
Vehicle	2	6	6	14
Uridine	6	9	0	15

χ^2 =8.576, p value =0.014

	Mild	Moderate	Severe	Total
Vehicle	0	5	4	9
Myricetin	7	5	2	14

χ^2 =6.906, p value =0.032

Movie S1. Representative QPM time lapse video of cardiac fibroblasts treated with ENPP1+ATP MCndM compared to vehicle MCndM. Video reveals a decrease in cell size and apoptosis of CFs within 24 hours of treatment ENPP1+ATP MCndM (treatment group) versus control MCndM (control group) (n=3).
